# TC-G 1008 facilitates epileptogenesis by acting selectively at the GPR39 receptor but non-selectively activates CREB in the hippocampus of pentylenetetrazole-kindled mice

**DOI:** 10.1007/s00018-023-04766-z

**Published:** 2023-04-25

**Authors:** Urszula Doboszewska, Katarzyna Socała, Mateusz Pieróg, Dorota Nieoczym, Jan Sawicki, Małgorzata Szafarz, Kinga Gawel, Anna Rafało-Ulińska, Adam Sajnóg, Elżbieta Wyska, Camila V. Esguerra, Bernadeta Szewczyk, Marzena Maćkowiak, Danuta Barałkiewicz, Katarzyna Mlyniec, Gabriel Nowak, Ireneusz Sowa, Piotr Wlaź

**Affiliations:** 1 Department of Animal Physiology and Pharmacology, Institute of Biological Sciences, Faculty of Biology and Biotechnology, Maria Curie-Sklodowska University, Akademicka 19, 20-033 Lublin, Poland; 2 Department of Analytical Chemistry, Medical University of Lublin, Chodźki 4A, 20-093 Lublin, Poland; 3 Department of Pharmacokinetics and Physical Pharmacy, Jagiellonian University Medical College, Medyczna 9, 30-688 Kraków, Poland; 4 Department of Experimental and Clinical Pharmacology, Medical University of Lublin, Jaczewskiego 8B, 20-090 Lublin, Poland; 5 Department of Neurobiology, Polish Academy of Sciences, Maj Institute of Pharmacology, Smętna 12, 31-343 Kraków, Poland; 6 Department of Trace Analysis, Adam Mickiewicz University, Uniwersytetu Poznańskiego 8, 61-614 Poznań, Poland; 7 Chemical Neuroscience Group, Centre for Molecular Medicine Norway, University of Oslo, Gaustadalléen 21, Forskningsparken, 0349 Oslo, Norway; 8 Laboratory of Pharmacology and Brain Biostructure, Department of Pharmacology, Maj Institute of Pharmacology, Polish Academy of Sciences, Smętna 12, 31-343 Kraków, Poland; 9 Department of Pharmacobiology, Jagiellonian University Medical College, Medyczna 9, 30-688 Kraków, Poland; 10 Present Address: Department of Pharmacobiology, Jagiellonian University Medical College, Medyczna 9, 30-688 Kraków, Poland

**Keywords:** Zinc chloride, Valproic acid, Maximal electroshock seizures, 6-Hz seizures, BDNF, TrkB

## Abstract

The pharmacological activation of the GPR39 receptor has been proposed as a novel strategy for treating seizures; however, this hypothesis has not been verified experimentally. TC-G 1008 is a small molecule agonist increasingly used to study GPR39 receptor function but has not been validated using gene knockout. Our aim was to assess whether TC-G 1008 produces anti-seizure/anti-epileptogenic effects in vivo and whether the effects are mediated by GPR39. To obtain this goal we utilized various animal models of seizures/epileptogenesis and GPR39 knockout mice model. Generally, TC-G 1008 exacerbated behavioral seizures. Furthermore, it increased the mean duration of local field potential recordings in response to pentylenetetrazole (PTZ) in zebrafish larvae. It facilitated the development of epileptogenesis in the PTZ-induced kindling model of epilepsy in mice. We demonstrated that TC-G 1008 aggravated PTZ-epileptogenesis by selectively acting at GPR39. However, a concomitant analysis of the downstream effects on the cyclic-AMP-response element binding protein in the hippocampus of GPR39 knockout mice suggested that the molecule also acts via other targets. Our data argue against GPR39 activation being a viable therapeutic strategy for treating epilepsy and suggest investigating whether TC-G 1008 is a selective agonist of the GPR39 receptor.

**Supplementary Information:**

The online version contains supplementary material available at 10.1007/s00018-023-04766-z.

## Introduction

In living organisms, zinc ions exist primarily as complexes with proteins. However, a small pool of intracellular and extracellular “free” or “labile” zinc is available for cell signaling [1]. The neuromodulatory function of extracellular zinc on numerous targets mediating neuronal excitation or inhibition was shown to be important in terms of seizures/epilepsy [2]. Moreover, extracellular zinc is thought to activate a G-protein-coupled receptor (GPCR), namely GPR39 [3, 4]. The receptor was cloned in 1997 [5] and has been linked to the peptide hormone obestatin, a supposed endogenous agonist [6]. Nevertheless, the findings have not been replicated [7, 8]. The existence of a GPCR activated by zinc ions has been postulated [9]; however, it is still debatable whether the physiological/pathophysiological concentrations of zinc are sufficient to activate GPR39 [10] and whether zinc is a genuine agonist or an allosteric effector [11].

Despite the controversies surrounding its endogenous ligand(s), as a receptor belonging to “druggable” GPCRs [12], GPR39 is gaining increasing attention as a target for future drugs in several therapeutic areas [13–16], including the central nervous system (CNS) [17–19]. Epilepsy is among the diseases that may find a cure by targeting the GPR39 protein. In vitro studies showed that GPR39 is critical for inhibitory neurotransmission [20, 21]. Ex vivo and in vivo data demonstrated the association between the expression/function of the receptor and acute seizures. Lithium chloride-pilocarpine-induced status epilepticus (SE) decreased the expression of the GPR39 protein in the hippocampus [22]. RNA sequencing revealed up-regulation of the *gpr39* gene in *stim2b* knockout zebrafish, which is hyperactive and more sensitive to treatment with pentylenetetrazole (PTZ) [23]. GPR39 knockout (KO) mice displayed enhanced susceptibility to seizures following a single intraperitoneal (i.p.) injection of kainic acid (KA), compared with wild-type (WT) littermates [21]. Based on this data, it was hypothesized that the activation of the GPR39 receptor represents a new strategy for inhibiting seizures [21, 24]. However, this strategy has not been verified experimentally to date. Furthermore, the role of the GPR39 gene in the development of epilepsy, i.e., epileptogenesis [25], has not been examined.

GPR39 is promiscuous in G-protein preferences, which means it interacts with more than one type of G-proteins [26], namely: G_s_, G_q_ and G_12/13_, in addition to binding β-arrestin [4, 27]. The small molecule agonist TC-G 1008 (synonyms: compound 3 [28], GPR39-C3 [29, 30]) activates cAMP production (downstream of G_s_), IP_3_ accumulation (downstream of G_q_), SRF-RE-dependent transcription (downstream of G_12/13_), and β-arrestin recruitment [28–30]. TC-G 1008 is the most widely used ligand in research on GPR39. It has, however, several off-targets [28], including the serotonin 5-HT_1A_ receptor [28, 29]. Like GPR39, 5-HT_1A_ is a promiscuous GPCR that signals via G_i_, G_s_, G_q_ and β-arrestin [31]. Consistent with the properties of promiscuous GPCRs [26, 32], the affinities of zinc and TC-G 1008 for GPR39 and 5-HT_1A_ vary depending on the signaling pathway measured [28, 29]. Moreover, zinc is a positive allosteric modulator for the activity of TC-G 1008 in β-arrestin, G_q_ and G_s_ pathway; the greatest dependency is observed in the G_s_ pathway [29]. Zinc is also an allosteric modulator of the responses mediated by 5-HT_1A_ [33]. These data strongly suggest that depending on local circumstances in tissues, TC-G 1008 can exert its action via GPR39 and/or off-targets, underscoring the difficulty in interpreting experimental data. Furthermore, it has not been validated in a GPR39 KO setting [34], raising the possibility that some effects observed after its administration may not be solely due to GPR39 activation. Importantly, 5-HT_1A_ is involved in the pathophysiology and treatment of epilepsy as shown with imaging studies of human brain and genetic ablation (KO) or pharmacological modulation of the receptor in animals [35, 36].

We addressed the pharmacological activation of GPR39 in the context of seizures/epileptogenesis and selectivity. First, we compared the effects induced by TC-G 1008 and the non-selective agonist, zinc chloride (ZnCl_2_), to a standard antiseizure drug, valproic acid (VPA), used to treat epilepsy, in a variety of acute seizure tests and a chronic epileptogenesis model (in vivo PTZ-kindling). We then assessed the response of GPR39 KO mice to seizure-inducing/epileptogenic agents and treatment with TC-G 1008, thus gaining more insight into the phenotype of GPR39 KO mice and the physiological role of the GPR39 gene. The experiment also allowed us to determine whether GPR39 is a target for TC-G 1008 in vivo.

Consistent with on-target mechanism, TC-G 1008 exacerbated PTZ-induced epileptogenesis by acting selectively at GPR39. This action contradicts the hypothesis that activation of GPR39 produces an anti-epileptogenic effect. However, it markedly increased the activation of the cyclic-AMP-response element binding (CREB) protein in the hippocampus of GPR39 KO mice in the PTZ-kindling model, thus showing non-selectivity. Our data demonstrated that TC-G 1008 acts at GPR39 and other targets. Furthermore, they imply that some of the earlier effects attributed to TC-G 1008 and the activation of GPR39 described in the literature may be related to its action at the 5-HT_1A_ receptor and/or other off-target(s).

## Materials and methods

### Reagents

TC-G 1008 was purchased from Adooq Bioscience LLC (Irvine, CA, USA). VPA (sodium salt), ZnCl_2_, PTZ, and KA were obtained from Sigma-Aldrich (St. Louis, MO, USA). For experiments in mice, ZnCl_2_, VPA, PTZ, and KA were dissolved in physiological saline (0.9% sodium chloride (NaCl) solution). TC-G 1008 was suspended in 1% Tween 80 solution in physiological saline. ZnCl_2_ and TC-G 1008 were dissolved in an embryo medium (Danieau’s buffer) for experiments in zebrafish. PTZ, 60 mM (3 × stock), was dissolved in the embryo medium. The materials used for biochemical analyses are listed in the sections on Liquid Chromatography-Tandem Mass Spectrometry (LC–MS/MS), Inductively Coupled Plasma Optical Emission Spectrometry (ICP-OES), Laser Ablation Inductively Coupled Plasma Mass Spectrometry (LA-ICP-MS), Zinpyr-1 (ZP-1) staining, and Western blot.

### Animals

The experiments were carried out with genetically unmodified mice, GPR39 KO mice, and zebrafish (*Danio rerio*) larvae. Housing and experimental procedures were in line with the European Union Directive of 22 September 2010 (2010/63/EU) and Polish and Norwegian legislative acts concerning animal experimentation. The experiments in mice were approved by the Local Ethics Committee in Lublin (experiments in genetically unmodified mice: approval numbers 38/2017, 48/2018, 110/2018, 36/2019; experiments in GPR39 KO mice: approval numbers 72/2019, 16/2020), and the I Local Ethics Committee in Warsaw (approval number 811/2019 regarding the generation of the GPR39 KO mouse line). The experiments using zebrafish were approved by the Norwegian Food Safety Authority experimental animal administration's supervisory and application system (“Forsøksdyrforvaltningen tilsyns- og søknadssystem”; FOTS ID 15469 and 23935). The animals were closely monitored by the animal caretakers and researchers, with regular inspection by a veterinarian, according to the standard health and animal welfare procedures of the local animal facility. All efforts were made to minimize animal suffering and the number of animals used in the study.

Experimentally naïve male Swiss Albino mice (n = 863) with a body weight range of 17–31 g were purchased from a licensed breeder (Laboratory Animals Breeding, Ilkowice, Poland). Animals were housed at the animal facility, Faculty of Biology and Biotechnology, Maria Curie Sklodowska University, Lublin, in groups of 7–8 in open Makrolon cages (37 cm × 21 cm × 14 cm) under strictly controlled laboratory conditions of light (12/12 h light–dark cycle), temperature (21–24 °C) and relative humidity (45–65%). Rodent chow diet (Murigran, Agropol S.J., Motycz, Poland) and tap water were provided ad libitum. The environment was enriched with nest material and paper tubes.

The GRP39 KO mouse model was generated by the Mouse Genome Engineering Facility (crisprmice.eu). A CRISPR/Cas 9 method was used to establish the model in a mixed genetic background (C57BL/6/Tar × CBA/Tar). A 44-bp deletion causing a p.Lys38fs*57X frameshift mutation was introduced. WT and KO mice were housed at the animal facility at the Experimental Medicine Center, Medical University of Lublin. Male WT mice (n = 35) and male KO mice (n = 35) weighing 16–29 g were used for experiments. The mice were housed in groups of 7–8 in open Makrolon cages (37 cm × 21 cm × 14 cm) under strictly controlled laboratory conditions of light (12/12 h light–dark cycle), temperature (21–24 °C), and relative humidity (45–65%). Diet (Altromin standard diet, Altromin, Lage, Germany) and tap water were provided ad libitum. The environment was enriched with nest material and paper tubes.

**Fig. 1:**
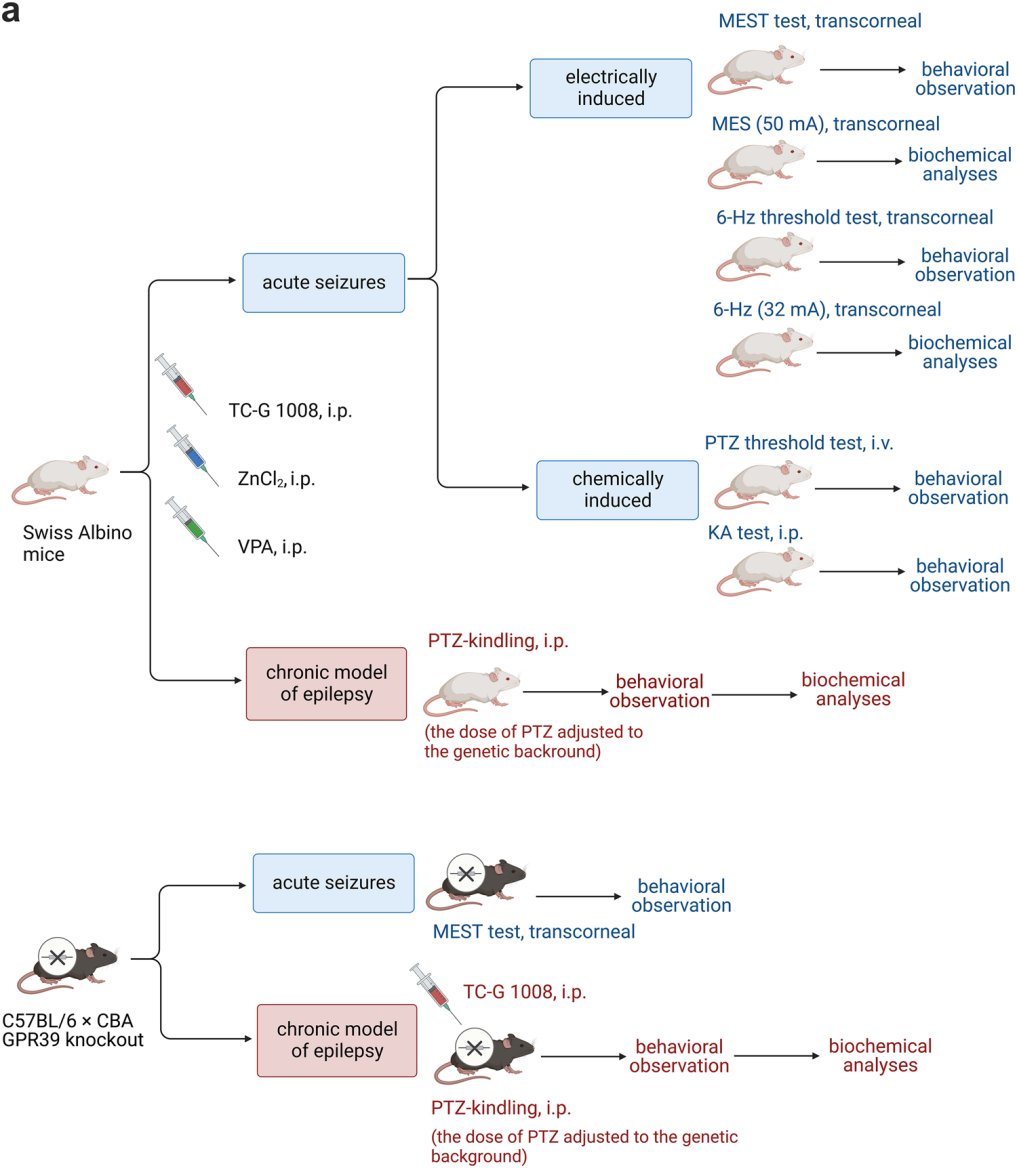

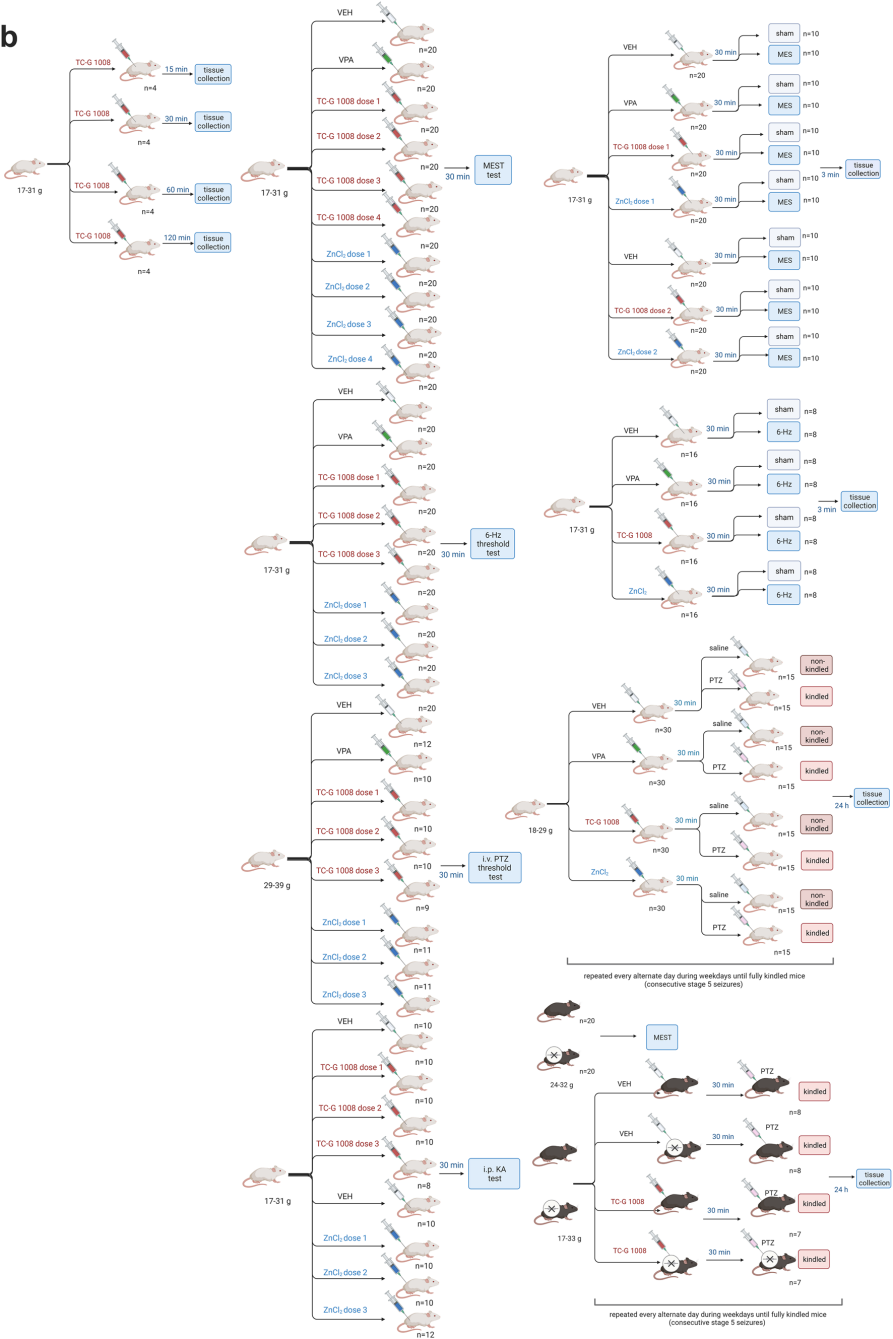
Experimental paradigm in mice. **a** Swiss Albino mice or C57BL/6/Tar × CBA/Tar GPR39 knockout and wild-type mice were subjected to acute models of electrically or chemically induced seizures or a chronic model of epileptogenesis [chemical kindling induced by pentylenetetrazole (PTZ)], followed by observation of seizure behavior. Animals were pre-treated with agonists of GPR39: the small molecule TC-G 1008 or ZnCl_2_ or a standard anti-seizure drug, valproic acid (VPA), i.p. **b** Detailed mouse allocation in each of the experimental procedures

The following procedures were performed in Swiss Albino mice: determination of serum and brain TC-G 1008 concentrations, maximal electroshock seizure (MES) threshold test (MEST), MES generated by supramaximal stimulus of 50 mA, 6-Hz-induced seizure threshold test, 6-Hz seizures generated by supramaximal current intensity of 32 mA, intravenous (i.v.) PTZ seizure threshold test, acute KA-induced seizures, PTZ kindling model, and biochemical analyses. The following procedures were performed in C57BL/6/Tar × CBA/Tar GPR39 KO and WT mice: MEST test, PTZ kindling, and biochemical analyses (Fig. 1). Animals were randomly assigned to the experimental groups. Blinding was not feasible during behavioral experiments due to the rotations of experimenters who either administered compounds or observed their behavioral effects. However, blinding was applied to the biochemical analyses.

**Fig. 2:**
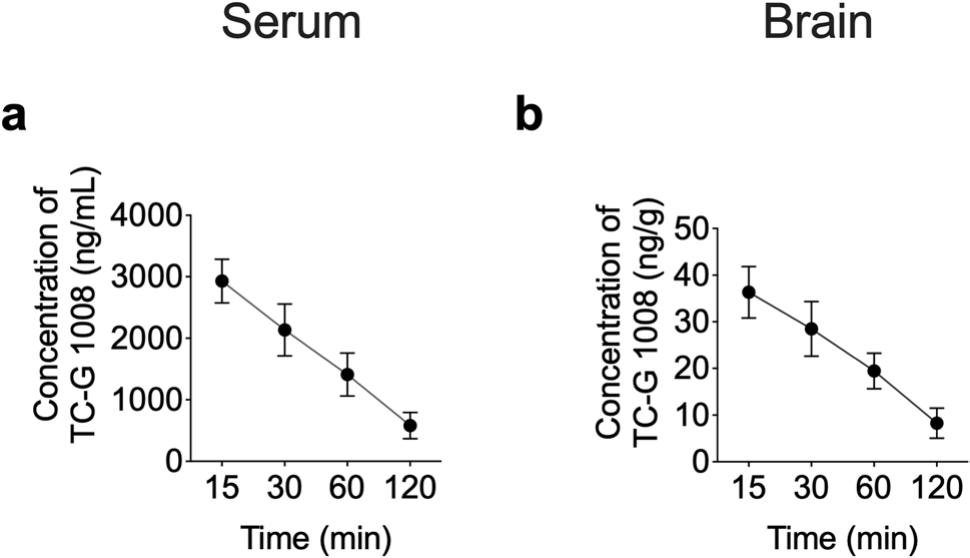
The concentrations of TC-G 1008 in the serum (**a**) and brain (**b**). Single dose of TC-G 1008 (20 mg/kg, i.p.) was administered in Swiss Albino mice. Data are expressed as means ± SEM of concentrations over time. n = 4 mice per time point

Before experimentation, animals were allowed to habituate to their new environment for at least one week. To minimize the impact of age on seizure susceptibility, young adult mice (1–3 months) were used in all experiments. To minimize possible interference of the estrous cycle on seizure susceptibility, only male Swiss Albino or C57BL/6/Tar × CBA/Tar mice were utilized [37]. Experiments were performed between 8:00 a.m. to 3:00 p.m., after a minimum 30-min adaptation period to the experimental room. Drug solutions/suspensions were prepared freshly and administered i.p. at a volume of 0.1 mL per 10 g of body weight. Control groups received vehicles (VEH) used to prepare drug solutions/suspensions. Drugs were administered 30 min before acute seizure tests, acute seizure models, or PTZ/KA injections. This pretreatment time was chosen after determining serum and brain TC-G 1008 concentrations (Fig. 2). Apart from using a 1% ophthalmic solution of tetracaine hydrochloride as a short-term topical ophthalmic anesthesia prior to determining seizure thresholds, performing MES, or 6-Hz seizure, no anesthetics or analgesics were used. This reduced the possibility of pharmacodynamic or pharmacokinetic interactions between the agents and compounds utilized. Each animal was used only once in the acute seizure test. Following acute seizure tests, surviving mice were euthanized using > 70% carbon dioxide (CO_2_) or cervical dislocation.

Stocks of the AB strain of adult zebrafish (*Danio rerio*) (Zebrafish International Resource Center, Eugene, OR, USA) were maintained following standard aquaculture conditions, i.e., 28.5 °C, 14/10 h light/dark cycle in 8.0 L tanks [27 cm long, 21 cm wide and 17 cm high]. Fertilized eggs were collected via natural spawning. Embryos were reared under constant light conditions in embryo medium [Danieau’s buffer: 1.5 mM Hepes, pH 7.6, 17.4 mM NaCl, 0.21 mM KCI, 0.12 mM MgSO_4_, and 0.18 mM Ca(NO_3_)_2_]. All embryos and larvae were maintained in a 28.5 °C incubator. The maximum tolerated concentration (MTC) was evaluated in n = 144 larvae 4 days post-fertilization (dpf). For local field potential (LFP) recordings, n = 79 larvae of 6 dpf were used.

### Maximal electroshock seizures

MES is a model of electrically induced, generalized tonic–clonic seizures [37]. Prior to MES, animals were administered a 1% ophthalmic solution of tetracaine hydrochloride as a short-term topical ophthalmic anesthesia followed by constant current stimuli (sine-wave pulses at 50 Hz for 200 ms) applied via saline-soaked transcorneal electrodes using a rodent shocker (type 221; Hugo Sachs Elektronik, Freiburg, Germany). During stimulation, mice were restrained manually, and immediately following stimulation, they were placed in a transparent box without bedding for behavioral observation for the presence or absence of seizure activity. Tonic hindlimb extension, defined as the rigid extension of the hindlimb that exceeds a 90° angle with the body, was considered as an endpoint. Two experimental approaches were used: (1) the MEST test that employed stimulation at varied current intensities (7.6–17.4 mA) and (2) MES that employed stimulation at a fixed current intensity (50 mA).

1. The mice were injected with a single dose of TC-G 1008, ZnCl_2_, VPA, or VEH (1% Tween 80 solution in physiological saline). 30 min later, the MEST test was performed. The threshold current was established according to an ‘up-and-down’ method described by Kimball et al. [38]. The current intensity was lowered or raised by 0.06-log intervals depending on whether the previously stimulated animal did or did not exert tonic hindlimb extension, respectively. The data obtained from groups of 20 animals were used to determine the threshold current causing an endpoint in 50% of mice (CS_50_ with confidence limits of 95% probability). In the MEST test, the dose–response relationship was assessed. An initial dose of TC-G 1008 or ZnCl_2_ was selected, and the dose was either increased or decreased in a subsequent group of mice, depending on whether the previous dose affected the seizure threshold. The dose of VPA has been established to increase the seizure threshold in this test [39]. Following MEST test, mice were euthanized with > 70% CO_2_.
2. Groups of mice (n = 10) were injected with a single dose of TC-G 1008, ZnCl_2_, VPA, or VEH. 30 min later, they were stimulated with a supramaximal MES stimulus of 50 mA. The doses of drugs applied before MES were based on the results of the MEST test—effective and ineffective doses of TC-G 1008 and ZnCl_2_ were administered. Control, non-stimulated (sham) animals received the respective doses of drugs or VEH but did not receive MES stimulus.

### Six hertz (6 Hz) seizures

The 6-Hz seizure is a model of electrically induced, focal seizures [40]. Before the 6-Hz seizure, animals were administered a 1% ophthalmic solution of tetracaine hydrochloride as a short-term topical ophthalmic anesthesia. Then, square-wave alternating current stimuli (0.2-ms duration pulses at 6 Hz for 3 s) was applied via saline-soaked transcorneal electrodes using a Grass model CCU1 constant current unit coupled to a Grass S48 stimulator (Grass Technologies, West Warwick, RI, USA). Mice were manually restrained during stimulation. Immediately following the stimulation, mice were placed in a transparent box without bedding for behavioral observation. The 6-Hz seizures were characterized by a stun (fixed) posture, rearing, forelimb clonus, twitching of the vibrissae, and Straub's tail. Absence of these characteristic features or the resumption of normal exploratory behavior within 10 s after stimulation was considered the absence of seizures. Two experimental approaches were used: (1) the 6-Hz seizure threshold test that employed stimulation at varied current intensities (10.0–20.0 mA) and (2) the 6-Hz seizures induced by supramaximal stimulation at a fixed current intensity (32 mA).

1. The mice were injected with a single dose of TC-G 1008, ZnCl_2_, VPA, or VEH (1% Tween 80 solution in physiological saline). 30 min later, the 6-Hz seizure threshold test was performed. The current intensity values were established using an ‘up-and-down’ method [38]. The animals were stimulated at a current intensity that was lowered or raised by 0.06-log intervals depending on whether the previously stimulated animal did or did not respond with seizures. The data obtained from groups of 20 animals were used to determine the threshold current causing 6-Hz-induced seizures in 50% of mice (CS_50_ with confidence limits of 95% probability). In the 6-Hz threshold test, the dose–response relationship was assessed. An initial dose of TC-G 1008 or ZnCl_2_ was selected, and the dose was either increased or decreased in a subsequent group of mice, depending on whether the previous dose affected the seizure threshold. The dose of VPA has been established to increase the seizure threshold in this test [39]. Following the 6-Hz seizure threshold test, the mice were euthanized with > 70% CO_2_.
2. Groups of mice (n = 8) were injected with a single dose of TC-G 1008, ZnCl_2_, or VPA, which was effective in the 6-Hz-seizure threshold test, or VEH. 30 min later, the mice were stimulated with a supramaximal current intensity of 32 mA. Control, non-stimulated (sham) animals received the respective doses of drugs or VEH but did not receive the supramaximal current.

### Kainic acid seizures

KA is an agonist for the KA subtype of ionotropic glutamate receptors. Administration of KA i.p. is a model of chemically induced, acute seizures. Groups of mice (n = 8–12) were injected i.p. with a single dose of TC-G 1008, ZnCl_2,_ or VEH (1% Tween 80 solution in physiological saline). 30 min later, the mice were injected i.p. with a single dose of KA (40 mg/kg) [41]. Immediately following the KA injection, mice were placed individually in a transparent box without bedding for 2 h for behavioral observation. The seizure severity of each mouse was scored according to the modified Racine’s scale: stage 0, no response; stage 1, immobility and staring; stage 2, scratching/myoclonic jerks; stage 3, forelimb clonus; stage 4, rearing; stage 5, rearing and falling; stage 6, jumping, circling, rolling; stage 7, SE/death [41–43]. The mean seizure severity scores were calculated, and the dose–response relationship was assessed. An initial dose of TC-G 1008 or ZnCl_2_ was selected and was either increased or decreased in a subsequent group of mice based on whether the previous dose exerted a response. The surviving animals were immediately euthanized with > 70% CO_2_.

### Intravenous pentylenetetrazole (PTZ) seizures

PTZ is an antagonist of the ionotropic GABA_A_ receptors. Infusion of PTZ in mice is a model of chemically induced, acute seizures. Groups of mice (n = 9–12) were injected i.p. with a single dose of TC-G 1008, ZnCl_2_, VPA, or VEH (1% Tween 80 solution in physiological saline). 30 min later, the mice were placed in a cylindrical plastic restrainer (12 cm long, 3 cm inner diameter). The lateral tail vein was catheterized with a 2-cm long 27-gauge needle attached by polyethylene tubing PE20RW (Plastics One Inc., Roanoke, VA, USA) to a 5-mL plastic syringe containing 1% aqueous solution of PTZ. The syringe was mounted on a syringe pump (model Physio 22, Hugo Sachs Elektronik–Harvard Apparatus GmbH, March-Hugstetten, Germany). The accuracy of needle placement in the vein was confirmed by the appearance of blood in the tubing. The needle was secured to the tail by adhesive tape. Following catheterization, mice were released from the restrainer and placed in a Plexiglas arena for behavioral observation. The PTZ solution was infused at a constant rate of 0.2 mL/min. The time intervals from the commencement of PTZ infusion to the onset of each of the three endpoints (myoclonic twitch, generalized clonus with loss of the righting reflex, and tonic forelimb extension) were recorded. The PTZ infusion was stopped at the beginning of tonic seizures, usually lethal for mice. All surviving animals were euthanized immediately by cervical dislocation. The seizure thresholds were calculated separately for each endpoint using the following formula: threshold dose of PTZ (mg/kg) = (infusion duration (s) × infusion rate (mL/s) × PTZ concentration (mg/mL))/body weight (kg) and were expressed as the dose of PTZ (in mg/kg) needed to produce the first apparent sign of each endpoint. The dose–response relationship was assessed. An initial dose of TC-G 1008 or ZnCl_2_ was selected and either increased or decreased in a subsequent group of mice, depending on whether the previous dose affected the seizure threshold. The dose of VPA has been established to increase the seizure threshold in this test [39]. Data obtained in the i.v. PTZ seizure threshold test are presented as the amount of PTZ (in mg/kg) ± SEM needed to produce the first apparent sign of each endpoint in each experimental group.

### PTZ kindling model of epilepsy

PTZ kindling in mice is a chemical kindling model resembling the chronic process of epilepsy development (epileptogenesis) [44]. In this model, a subthreshold dose of PTZ is administered repeatedly i.p. It induces focal seizures at the beginning of the procedure. The seizures evolve in severity and duration with repeated exposure to PTZ, and finally, generalized tonic–clonic seizures are observed.

**Fig. 3:**
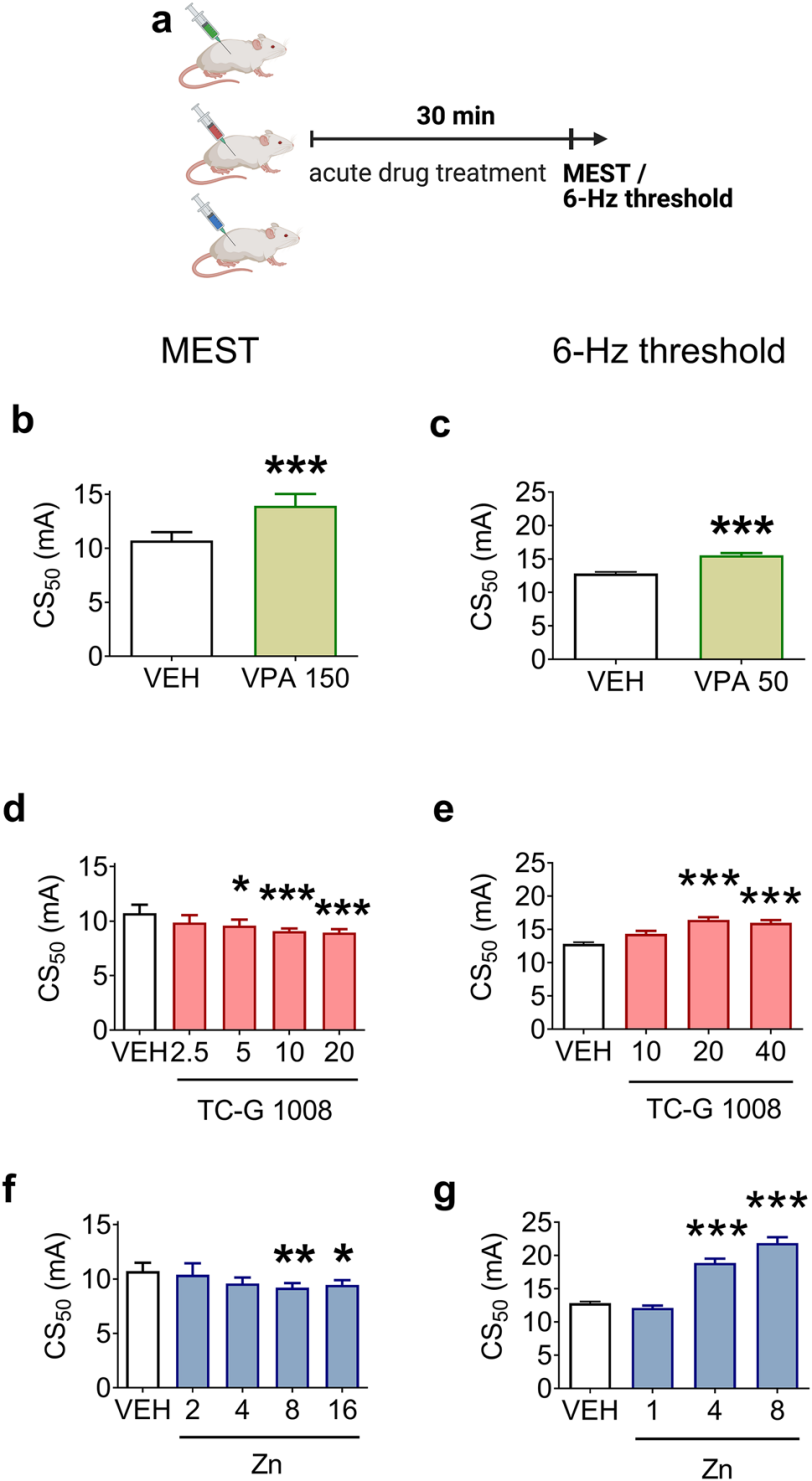
The effects of single doses of VPA (**b**, **c**), TC-G 1008 (**d**, **e**), or ZnCl_2_ (**f**, **g**) on the seizure threshold in the maximal electroshock seizure threshold (MEST) test and the seizure threshold in the 6-Hz seizure threshold test. **a** Drugs or VEH were administered i.p. in Swiss Albino mice. 30 min later, the MEST or 6-Hz seizure threshold test was performed. **b**–**g** The doses of drugs are shown on the x-axes in mg/kg. Data are presented as CS_50_ (in mA) values with upper 95% confidence limits. n = 20 in each group. p values were determined by the Student’s t-test or one-way ANOVA and Dunnett’s multiple comparison test. *p < 0.05, **p < 0.01, ***p < 0.001

The mice were injected i.p. with VEH (1% Tween 80 solution in physiological saline), TC-G 1008, ZnCl_2_, or VPA on every alternate day during weekdays. Because PTZ-kindling produces generalized tonic–clonic seizures, also observed after the application of MES, the doses of drugs were based on the results of the MEST test. The lower doses of TC-G 1008 (10 mg/kg) or ZnCl_2_ (8 mg Zn/kg), which were effective in this test (Fig. 3d, f), were selected for the chronic experiment. The dose of VPA (150 mg/kg) has been established to inhibit epileptogenesis in this model [45].

30 min after drug administration, the mice were injected i.p. with a subthreshold dose of PTZ, which was adjusted to the strain’s genetic background. The subthreshold doses of PTZ, which induce the kindling phenomenon, range from 25 to 45 mg/kg due to differences in responsiveness to the chemoconvulsant between strains [44]. In the case of kindling in Swiss Albino mice, the subthreshold dose of PTZ was 40 mg/kg, as determined in our previous study [45]. For C57BL/6/Tar × CBA/Tar mice, the subthreshold dose of PTZ was 25 mg/kg, as determined in a pilot experiment. Immediately following PTZ injection, mice were placed individually in a transparent box without bedding for 30 min for behavioral observation. The seizure severity of each mouse was scored using the modified Racine’s scale: stage 0, no response; stage 1, immobility, ear and facial twitching; stage 2, myoclonic jerks; stage 3, forelimb clonus; stage 4, clonic seizure with rearing and falling; stage 5, generalized clonic seizure with loss of righting reflex; stage 6, tonic fore- and hindlimb extension [45]. The mean seizure severity scores were calculated for all experimental groups after each PTZ injection. Control, non-kindled animals received the respective doses of drugs but were injected with physiological saline instead of PTZ solution. The PTZ kindling model was terminated to reduce mortality when the groups displayed consecutive stage 5 seizures and were considered fully kindled. The Swiss Albino and the WT group of C57BL/6/Tar × CBA/Tar mice that received TC-G 1008 displayed the highest seizure score during the respective PTZ kindling models. 15 Swiss Albino mice per group or 15 WT, and 15 GPR39 KO mice were used in these models.

### Grip-strength test

The effects of single doses of TC-G 1008, ZnCl_2_, or VPA or repeated doses of these compounds and PTZ kindling on skeletal and muscular strength were evaluated in Swiss Albino or C57BL/6/Tar × CBA/Tar mice using the grip-strength test [46]. The grip-strength apparatus (BioSeb, Chaville, France) consisted of a steel wire grid (8 × 8 cm) connected to an isometric force transducer. A mouse was lifted by its tail to grasp the grid with its forepaws. The mouse was then gently pulled back until it released the grid, and the maximal force in newtons (N) exerted by the mouse before losing grip was measured. The procedure was repeated three times, and the mean force exerted by each mouse before losing grip was recorded. The mean force was normalized to body weight and expressed in mN/g ± SEM.

### Chimney test

The effects of single doses of TC-G 1008, ZnCl_2_, or VPA or repeated doses of these compounds and PTZ kindling on motor deficits were evaluated in Swiss Albino or C57BL/6/Tar × CBA/Tar mice using the chimney test [46]. In this test, the inability of animals to climb backward up through a Plexiglas tube (3 cm inner diameter × 30 cm length) within 60 s depicted motor impairment.

### PTZ seizures in zebrafish

Application of PTZ to zebrafish larvae is a model of chemically induced acute seizures. In this model, the brain’s electrical activity is measured by LFPs. LFP (also called micro- or depth-electroencephalography) is recorded in the extracellular space in brain tissue using a microelectrode, in contrast to electroencephalogram (EEG), which is recorded at the surface of the scalp [47]. PTZ produces ictal-like discharges in zebrafish larvae which are monitored by LFPs [48, 49].

MTC was evaluated prior to experiments in zebrafish larvae. Groups (n = 12) of 4 dpf zebrafish larvae were incubated with a range of TC-G 1008 or ZnCl_2_ doses at 28.5˚C for 18 h. Six different doses of each compound (TC-G 1008 or ZnCl_2)_ were tested. The following parameters were scored after 2 and 18 h of exposure: touch response, posture, edema, morphology, signs of necrosis, swim bladder, and heartbeat. The doses of 65 µM Zn and 70 µM TC-G 1008 were chosen as MTCs and were used for LFP recordings.

A single 6 dpf zebrafish larvae were placed in a 48-well plate (one larva per well) filled with 200 µL of VEH (embryo medium, i.e., Danieau’s buffer), ZnCl_2_ or TC-G 1008 solution. Subsequently, larvae were incubated for 20 h at 28.5 °C. After incubation, larvae were exposed to VEH or 20 mM PTZ for 5 min. Thereafter, larvae were immobilized in a thin layer of 2% low-melting-point agarose. A glass electrode (resistance 1–5 MΩ) filled with artificial cerebrospinal fluid (124 mM NaCl, 2 mM KCl, 2 mM MgSO_4_, 2 Mm CaCl_2_, 1.25 mM KH_2_PO_4_, 26 mM NaHCO_3_, 10 mM glucose) was then placed into the optic tectum (MultiClamp 700B amplifier, Digidata 1550 digitizer, Axon Instruments, Inc., Burlingame, CA, USA) [50]. Single recordings for each larva were carried out for 20 min. The discharges were analyzed according to the duration of spiking paroxysms, and only those where the amplitude exceeded three times the background noise were considered. The data were analyzed using Clampfit 10.2 software (Molecular Devices, LLC, San Jose, CA, USA) and a custom-written program for R (Windows). For LFP recordings, n = 5–17 larvae per group were used.

### Tissue processing for biochemical analyses

The biochemical analyses were performed on mouse tissue. For LC–MS/MS analysis, mice were killed by rapid decapitation 15, 30, 60, or 120 min after i.p. injection of TC-G 1008. For Western blot, ZP-1 staining, and ICP-OES analysis, the mice were killed ca. 3 min after MES or 6-Hz seizure or 24 h after the completion of the kindling paradigm. For LA-ICP-MS analysis, mice were killed 24 h after the completion of the kindling paradigm. Mice brains were rapidly dissected and immersed in cold (2–8 °C) 0.9% NaCl solution. For LC–MS/MS analysis, the whole brains were frozen. For Western blot, ZP-1 staining, and LA-ICP-MS, the brains were rapidly dissected on a cold plate and separated into left and right hemispheres. Left hippocampi (dorsal and ventral) were dissected, frozen on dry ice, and stored at − 80 °C until Western blot analysis. The right hemispheres were flash-frozen in liquid nitrogen and stored at − 80 °C until ready for cryo-sectioning. 12 µm hippocampal coronal sections were prepared from right hemispheres using cryostat microtome Leica CM 1850, Germany, and were attached to glass slides (SuperFrost microscope slides, cut edges, Thermo Scientific Menzel Glaser). The glass slides were stored at − 80 °C until further analysis. Trunk blood was collected into tubes without anticoagulant. The blood was allowed to clot for 15–20 min and then centrifuged for 10 min at 2000×*g* at 4 °C. The resulting supernatant (serum) was transferred into tubes and stored at − 80 °C until LC–MS/MS or ICP-OES analysis. The biochemical analyses were performed by experimenters blinded to the treatment.

### Determination of TC-G 1008 concentrations by LC–MS/MS

Brain and serum concentrations of TC-G 1008 were determined in Swiss Albino mice 15, 30, 60, and 120 min after administration of the compound (20 mg/kg, i.p.) using the LC–MS/MS method. The brains were homogenized in distilled water (1:3, *w*/*v*) with a tissue homogenizer TH220 (Omni International, Inc., Warrenton, VA, USA). Purification of the samples was done using a protein precipitation procedure with 0.1% formic acid in acetonitrile containing pentoxifylline as an internal standard (2000 ng/mL; added to the samples at a ratio of 1:2 (*v/v*)). Then, the samples were shaken for 10 min (IKA Vibrax VXR, Germany) and centrifuged (Minispin, Eppendorf, Germany) for 10 min at 15,000×*g*. The supernatant was transferred into autosampler vials for high-performance liquid chromatography (HPLC). The HPLC system (Agilent 1100, Agilent Technologies, Waldbronn, Germany) consisted of a degasser, binary pump, column oven, and an autosampler. Chromatographic separation was carried out on an XBridge™ C18 analytical column (3 × 50 mm, 5 µm, Waters, Ireland) with the oven temperature set at 30 °C. The autosampler temperature was maintained at 10 °C, and a sample volume of 15 μL was injected into the LC–MS/MS system. The mobile phase containing 0.1% formic acid in acetonitrile (A) and 0.1% formic acid in water (B) was set at a flow rate of 0.4 mL/min. Initial mobile phase composition was 95% B with a linear gradient to 30% B in the first 5 min, then isocratic mode for 5 min with the subsequent rapid change back to 95% B in 0.1 min. The remaining time of elution was set at 95% B. The whole HPLC operation lasted 13 min. Mass spectrometric detection was performed on an Applied Biosystems MDS Sciex (Concord, Ontario, Canada) API 2000 triple quadrupole mass spectrometer equipped with an electrospray ionization (ESI) interface. ESI ionization in the positive ion mode was used for ion production. The tandem mass spectrometer was operated at unit resolution in the selected reaction monitoring mode (SRM), monitoring the transition of the protonated molecular ions *m/z* 419 to 305 and *m/z* 419 to 171 for compound TC-G 1008 (the first pair was used as a quantifier and the second pair was employed for the identity verification as a qualifier) and *m/z* 279 to 181 for the internal standard. The mass spectrometric conditions were optimized for TC-G 1008 by continuous infusion of the standard solution at a rate of 10 μL/min using a Harvard infusion pump. The ion source temperature was maintained at 400 °C. The ion spray voltage was set at 5500 V. The curtain gas (CUR) was set at 20 psi, and the collision gas (CAD) at 12 psi. The optimal collision energy (CE) was 45 V. The following parameters of the ion path were used as the most favorable ones: declustering potential (DP) at 31 V, focusing potential (FP) at 340 V and entrance potential (EP) at 6.5 V. Data acquisition and processing were accomplished using the Applied Biosystems Analyst version 1.6 software. The calibration curves were constructed by plotting the ratio of the peak areas of TC-G 1008 to the internal standard versus TC-G 1008 concentrations and generated by weighted (1/x) linear regression analysis. The validated quantitation ranges for this method were from 1 to 2000 ng/mL for serum and 3–1500 ng/g for brain tissue, with accuracy from 89.88 to 110.13% and 86.54–109.51% for serum and brain tissue, respectively. The assays were reproducible with low intra- and inter-day variation (coefficient of variation less than 15%). No significant matrix effect was observed, and there were no stability-related problems during the routine analysis of samples.

### Determination of total zinc concentration by ICP-OES

Total zinc concentration in serum was determined by ICP-OES. Serum samples of Swiss Albino or C57BL/6/Tar × CBA/Tar mice were defrosted. 200 µL of serum was transferred to digestion vessels (DigiTUBE SCP SCIENCE 50 mL class A) and mixed with 1.5 mL of 65% Suprapur^®^ nitric acid (Merck) and 5.0 mL of deionized water. Then the vessels were placed in heating blocks (DigiPREP SCP SCIENCE) and digested for 60 min at 120℃. After digestion, vessels were left to reach room temperature (RT) and filled with deionized water to 10 mL. Samples were analyzed using PlasmaQuant PQ 9000 Analytik Jena AG. The following operating conditions of ICP-OES were used: power 13,000 W, plasma gas 14.0 L/min, auxiliary gas 0.50 L/min, nebulizer gas 0.60 L/min, and the monitoring direction of the plasma flame was axial. The standard solution for calibration curves of zinc at the concentration of 200 µg/L was prepared by diluting the zinc standard (1000 mg/L; PlasmaCAL SCP SCIENCE) with 0.5% nitric acid in deionized water. The analysis line used for zinc quantification was 206.2 nm.

### Determination of total zinc concentration by LA-ICP-MS

Total zinc concentration in hippocampal sections was determined semi-quantitatively by LA-ICP-MS. 12 µm hippocampal coronal sections from C57BL/6/Tar × CBA/Tar mice were thawed and dried at RT in a desiccator and placed in an ablation chamber. The sections were analyzed using a laser ablation (LA) system (LSX-500, CETAC Technologies, Omaha, NE, USA) with a quadrupole inductively coupled plasma mass spectrometer (ICP-MS, Elan DRC II, PerkinElmer SCIEX, Toronto, ON, Canada). The instruments were optimized daily with the use of a certified reference material of NIST SRM 610 glass, which included the nebulizer gas flow, ion lens voltage, and power of the plasma generator, and were tuned until reaching the maximal intensity for ^24^Mg^+^, ^115^In^+^, ^238^U^+^, and the oxide ratios of ^232^Th^16^O^+^/^232^Th^+^  < 0.2%, as well as doubly charged ions ^42^Ca^2+^/^42^Ca^+^  < 0.2%. The following isotopes were monitored in all measurements: ^13^C, ^66^Zn. The laser parameters were optimized to ablate the thin sections completely and to obtain measurable signals for the analyzed elements, which required the optimization of the following laser parameters: laser energy, laser spot size, frequency of laser shots, and sample scan rate. The instrumental parameters of LA-ICP-MS were as follows: laser energy 2.7 mJ, spot size 100 µm, ablation frequency 2 Hz, scan rate 100 µm/s, nebulizer gas flow 0.9 L/min, plasma power 1250 W, pulse counting mode, dwell time 100 ms per isotope, measured isotopes ^13^C and ^66^Zn. The laser beam scanned a rectangular area of the sample line by line and always from left to right. The number and width of the ablation lines were set individually for each analyzed sample, with 18 lines on average. The detector recorded a time-resolved signal to create a two-dimensional matrix of data points for each sample. For the statistical evaluation of the measurement data, the region of interest (ROI) containing the hippocampus was marked on maps of the distribution of elements in thin brain sections. The ROIs were selected by drawing the shape in the imaging software tool based on the sample photograph taken before ablation. The signals contained in the ROI were averaged and evaluated statistically.

### Determination of free intracellular zinc concentration by Zinpyr-1 staining

Free intracellular zinc concentration ([Zn^2+^]_I_) was examined in hippocampal sections by staining with a cell-membrane permeable fluorescent probe for zinc, ZP-1. 12 µm hippocampal coronal sections from Swiss Albino or C57BL/6/Tar × CBA/Tar mice were incubated with 4% paraformaldehyde solution with 4% sucrose in phosphate-buffered saline (PBS) at RT for 15 min. Sections were then rinsed with 0.01 M PBS solution and incubated with a solution of ZP-1 (Santa Cruz Biotechnology, sc-213182) at a concentration of 5 μM for 1 h at RT. The sections were double stained with 4′,6-diamidino-2-phenylindole dihydrochloride (DAPI) (Sigma Aldrich, D9542) at a concentration of 300 nM. Adjacent sections from the same mouse brain were treated with the membrane-permeable zinc chelator N,N,N′,N′-tetrakis(2-pyridylmethyl)ethylenediamine (TPEN) (Santa Cruz Biotechnology, sc-200131), at a concentration of 10 μM, for 40 min, before staining with ZP-1 and DAPI [51]. The sections were imaged using a Leica DM6000 B microscope. The images of all compared sections were taken with the same exposure time, and so were the images of sections incubated with TPEN before staining with ZP-1. The low-magnification, grayscale images were analyzed for the mean ZP-1 intensities using Image J software. The following regions of the hippocampus were chosen for the analysis: dentate gyrus (DG), CA1, and CA3 regions.

### Western blot

Hippocampi of Swiss Albino or C57BL/6/Tar × CBA/Tar mice were homogenized in 2% sodium dodecyl-sulfate solution (SDS) (BioShop Canada Inc), denatured at 95 °C for 10 min and centrifuged at 11,000×*g* at 4 °C for 5 min. The total protein concentration was quantified in the supernatant using a Pierce BCA Protein Assay Kit (Thermo Fisher Scientific, Pierce Biotechnology, Rockford, IL, USA). The samples containing 10 µg of protein were prepared using Novex^®^ Tris–Glycine SDS Sample Buffer (Thermo Fisher Scientific, Carlsbad, CA, USA) and resolved on a 4–15% Mini-Protean TGX Precast gels (BIO-RAD Laboratories, Inc., Hercules, CA, USA). A molecular weight marker: Spectra Multicolor Broad Range Protein Ladder (Thermo Fisher Scientific Baltic, Vilnius, Lithuania) was used. The proteins were transferred onto a nitrocellulose membrane (BIO-RAD Laboratories, Inc., Hercules, CA, USA). The membranes were blocked for 60 min with 1% blocking reagent from the BM Chemiluminescence WB kit (Mouse/Rabbit) (Roche Diagnostic, Mannheim, Germany). The membranes were then incubated in rabbit polyclonal antibody targeting metal regulatory transcription factor 1 (MTF1) (Novus Cat# NBP1-86379, RRID:AB_11033871, at a concentration of 0.2 μg/mL) or mouse monoclonal antibody targeting phosphorylated CREB at Ser 133 (anti-phospho-CREB (Ser133) antibody, clone 10E9, Millipore Cat# 05-667, RRID:AB_309889, at a concentration of 0.5 µg/mL) or rabbit monoclonal antibody targeting CREB (anti-CREB antibody, Abcam Cat#ab32515, RRID:AB_2292301, at a dilution of 1:1000) or rabbit polyclonal antibody targeting brain derived neurotrophic factor (BDNF) (anti-BDNF antibody, Novus Cat# NB100-98682, RRID:AB_1290643, at a dilution of 1:1000), or rabbit polyclonal antibody targeting tyrosine-phosphorylated tropomyosin receptor kinase B (TrkB) (anti-Trk B phosphorylated (pTyr 816) antibody, Novus Cat# NBP1-03499, RRID:AB_1522601, at a concentration of 10 µg/mL), or rabbit monoclonal antibody targeting TrkB (anti-TrkB antibody, Abcam Cat#ab187041, RRID: AB_2892613, at a dilution of 1:5000), or rabbit polyclonal antibody targeting extracellular signal-regulated kinase 1/2 (ERK1/2) (Cell Signaling Technology Cat# 9102, RRID:AB_330744, at a dilution of 1:1000), or rabbit monoclonal antibody targeting phosphorylated (Thr202/Tyr204 Thr185/Tyr187) ERK1/2 (Millipore Cat# 05-797R, RRID:AB_1587016, at a dilution 1:1000) or rabbit polyclonal antibody targeting sirtuin 1 (SIRT1) (BT Lab Cat# BT-AP08324, at a dilution 1:1000) or β-actin (β-actin antibody, mouse monoclonal clone AC-15, purified from hybridoma cell culture**,** Sigma-Aldrich Cat# A1978, RRID:AB_476692, at a concentration of 0.5 µg/mL**)** at 2–8 °C overnight. The dilutions of primary antibodies were prepared using 0.5% blocking solution from the BM Chemiluminescence WB kit (Mouse/Rabbit) (Roche Diagnostic, Mannheim, Germany). The dilutions of primary antibodies were stored at 2–8 °C and reused up to two times. The next day, membranes were washed in TBST (3 × 10 min) and incubated for 30 min in horseradish peroxidase-linked (HRP-linked) secondary antibody from the BM Chemiluminescence WB kit (Mouse/Rabbit), at a concentration of 40 mU/mL, or the anti-mouse IgG, HRP-linked, Cell Signaling Cat# 7076, RRID:AB_330924, at a dilution of 1:1000 at RT. The dilutions of secondary antibodies were always freshly made. After incubation with secondary antibodies, the membranes were washed in TBST 3 × 10 min. Protein bands on the membranes were detected using a BM Chemiluminescence WB kit (Mouse/Rabbit) and visualized with the Fuji-LAS 4000 System (Fuji, Tokyo, Japan) or the ChemiDoc Imaging System (BIO-RAD Laboratories, Inc., Hercules, CA, USA). The density of each protein band was analyzed using an imaging software (Fuji Image Gauge, Fuji, Tokyo, Japan v 4.0 or Image Lab, BIO-RAD Laboratories, Inc., Hercules, CA, USA) and normalized to the optical density of the corresponding β-actin band or by the total protein concentration.

### Data and statistical analyses

Data were analyzed using GraphPad Prism v. 5.03 (GraphPad Software, San Diego, CA, USA) or STATISTICA v. 13.3 (TIBCO Software Inc, Palo Alto, CA, USA). No statistical method was used to predetermine the sample size. Data were screened for outliers using the Grubbs’s test (https://www.graphpad.com/quickcalcs/Grubbs1.cfm). The outliers were not used in the analyses. Data were analyzed for normality of residual errors using skewness and kurtosis. The degree of departure from normality was not significant, thus allowing the use of a general linear model. Acute seizure tests and grip-strength tests in Swiss Albino or C57BL/6/Tar × CBA/Tar mice were analyzed using unpaired Student’s *t*-test or one-way analysis of variance (ANOVA) and Dunnett’s multiple comparison test. Kindling in Swiss mice was analyzed by two-way ANOVA and Dunnett’s multiple comparison test. Kindling in C57BL/6/Tar × CBA/Tar mice was analyzed by three-way ANOVA and Bonferroni’s multiple comparison test. The Fisher's exact probability test (https://www.graphpad.com/quickcalcs/contingency2) or the Chi-square test was employed to analyze the chimney test or the percentage of animals displaying consecutive stage 5 seizures (fully kindled mice in the PTZ-kindling model) or stage 7 seizures (in the acute KA-seizure model). Data obtained in zebrafish larvae were analyzed by two-way ANOVA and Bonferroni’s multiple comparison test. For Western blot, ZP-1 staining, ICP-OES, or LA-ICP-MS, each sample was run at least twice. These experiments were analyzed by two-way ANOVA and Bonferroni’s multiple comparison test. The results are presented as the mean ± SEM. p < 0.05 was considered statistically significant with 95% confidence.

## Results

### TC-G 1008 is brain penetrant

Penetration into the brain is a prerequisite for a potential drug targeting the CNS. To ensure that TC-G 1008 penetrates into the brain tissue, we measured the concentration versus time profiles of TC-G 1008 in the serum and brain of Swiss Albino mice following i.p. administration of a single dose of TC-G 1008 (20 mg/kg) (Fig. 2). The mean concentration of TC-G 1008 in the brain was 36.32 ng/g 15 min after administration and 28.48 ng/g 30 min after dosing (Fig. 2b). The molecular weight of TC-G 1008 is 418.9 Da. The estimated EC_50_ values are 0.4 and 0.8 nM for rat and human receptors, respectively [28], corresponding to 0.168 ng/mL and 0.335 ng/mL. These data show that the concentration of TC-G 1008 attained in the brain after i.p. administration of 20 mg/kg is sufficient to occupy the GPR39 receptor. The mean concentrations of TC-G 1008 in serum were 2930 ng/mL and 2135 ng/mL after 15 and 30 min, respectively (Fig. 2a). The serum levels of TC-G 1008 were close to those obtained following oral administration in male C57/Bl6 mice [28]. For example, the dose-normalized concentration at 60 min was 0.071 in this study and 0.06 with an oral dose of 10 mg/kg [28]. These data indicate that the bioavailability of TC-G 1008 is comparable for both routes of administration.

The pharmacokinetic parameters of TC-G 1008 after a dose of 20 mg/kg i.p. in Swiss Albino mice estimated using the non-compartmental approach are shown in Table S1. TC-G 1008 was slowly eliminated from serum and brain as the terminal half-life was about 50 min and the mean residence time values were over 70 min. The volume of distribution was high and significantly exceeded mouse whole body water, thus showing an extensive distribution of TC-G 1008 in organs and tissues. However, the brain tissue penetration was limited as the brain-to-serum AUC ratio was 0.014. Nevertheless, the TC-G 1008 concentration in the brain was sufficient to exert pharmacological effects at GPR39, which prompted us to examine its behavioral effects.

### TC-G 1008 and ZnCl_2_ exert divergent effects in the MEST and 6-Hz seizure threshold tests

#### TC-G 1008 and ZnCl_2_, unlike VPA, decrease the seizure threshold in the MEST test

We began our research by checking whether the pharmacological activation of GPR39 influences behavioral seizures in mice. We examined the effects of single doses of GPR39 agonists (TC-G 1008 and ZnCl_2_) in the MEST and 6-Hz-seizure threshold tests (Fig. 3). Both tests are screening paradigms routinely used to characterize investigational compounds with potential anti-seizure properties [52]. Simultaneously, we compared the effects of TC-G 1008 and ZnCl_2_ to the effects of a standard anti-seizure drug, VPA (Fig. 3). Because anti-seizure drugs may impact motor coordination and neuromuscular strength, we examined the acute effects of the compounds in the chimney and grip strength tests, respectively. Because TC-G 1008 was detected in brain tissue 30 min after i.p. administration in Swiss Albino mice at a concentration sufficient to occupy the GPR39 receptor (Fig. 2b), we examined the effects of the compounds on seizure threshold 30 min after their administration (Fig. 3a).

A standard anti-seizure drug, VPA (150 mg/kg), significantly increased the threshold for tonic hindlimb extension in the MEST test [t(17) = 5.037, p = 0.0001, Student’s t-test] (Fig. 3b). One-way ANOVA showed significant effects of TC-G 1008 [F(4,44) = 7.202, p = 0.0001] and ZnCl_2_ [F(4,43) = 4.080, p = 0.0069] in this test. In contrast to VPA (150 mg/kg), TC-G 1008 at doses of 5, 10, and 20 mg/kg (Fig. 3d) and ZnCl_2_ at doses of 8 and 16 mg Zn/kg (Fig. 3f) decreased the threshold for seizures in this test. These data showed a higher risk of experiencing MES after the administration of TC-G 1008 or ZnCl_2_.

#### TC-G 1008, ZnCl_2_, and VPA, increase seizure threshold in the 6-Hz seizure threshold test

In the 6-Hz seizure threshold test, VPA (50 mg/kg) significantly increased the threshold for focal seizures [t(18) = 4.336, p = 0.0004, Student’s t-test] (Fig. 3c). One-way ANOVA demonstrated significant effects of TC-G 1008 [F(3,35) = 9.292, p = 0.0001] and ZnCl_2_ [F(3,31) = 38.98, p < 0.0001] in this test. Like VPA (50 mg/kg), TC-G 1008 at doses of 20 and 40 mg/kg (Fig. 3e), as well as ZnCl_2_ at doses of 4 and 8 mg Zn/kg (Fig. 3g), increased the seizure threshold. These data indicated a lower risk of experiencing 6-Hz induced seizures after administering TC-G 1008 or ZnCl_2_, thus revealing divergent effects of the GPR39 agonists (TC-G 1008 and ZnCl_2_) in the MEST and 6-Hz threshold tests.

The effects of single doses of the examined compounds on motor coordination and neuromuscular strength are shown in Table S2. None of the compounds significantly impaired motor coordination of mice in the chimney test. In addition, single doses of VPA had no effects on the neuromuscular strength in the grip-strength test, while TC-G 1008 at 10, 20, and 40 mg/kg significantly increased the neuromuscular strength of mice. Similarly, a single dose of ZnCl_2_ (16 mg Zn/kg) increased the neuromuscular strength of mice.

### ZnCl_2_ increases maximal seizure severity and the incidence of SE in response to kainic acid

**Fig. 4:**
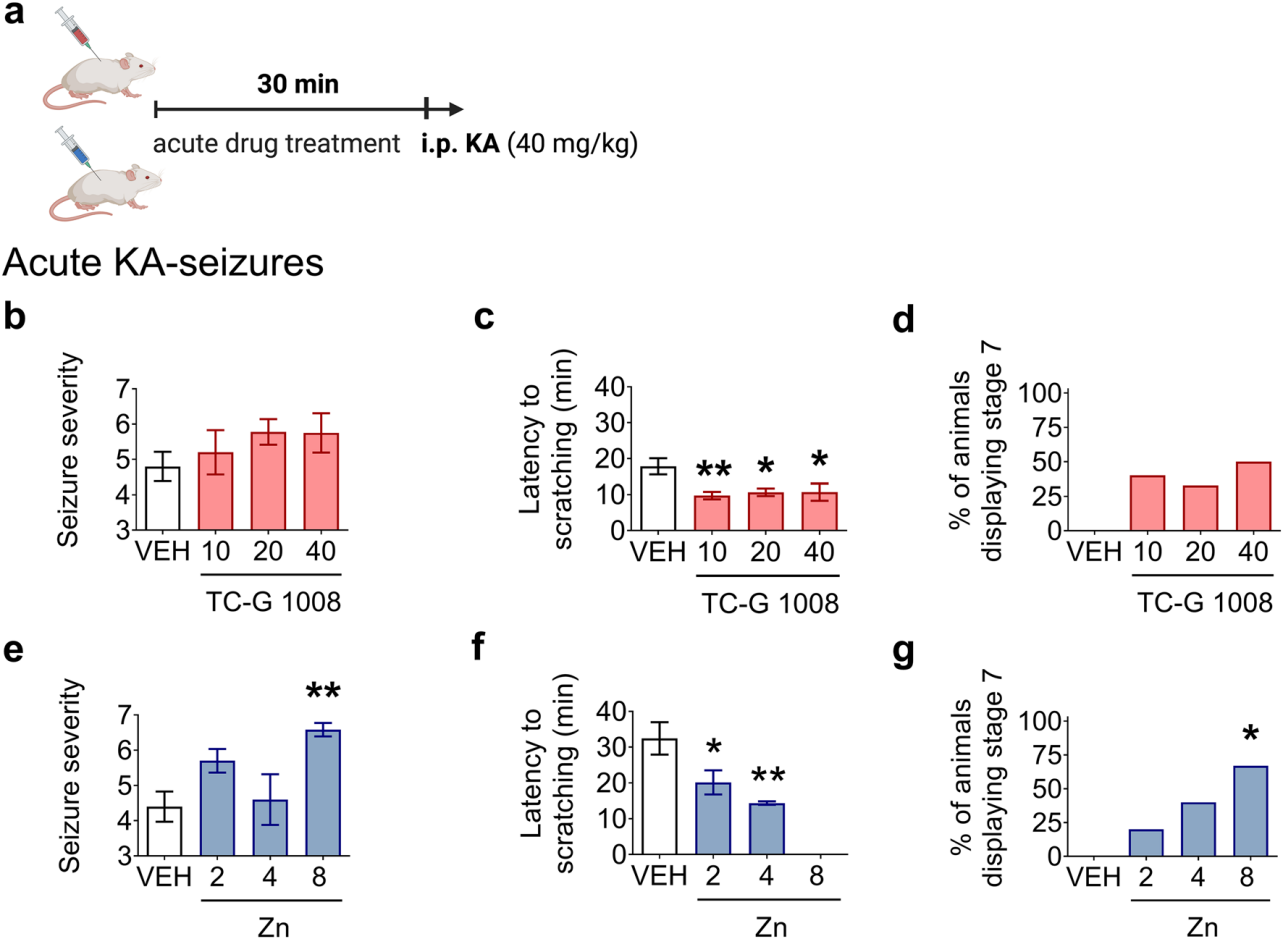
The effects of single doses of TC-G 1008 (**b**–**d**) or ZnCl_2_ (**e**–**g**) on acute seizures induced by kainic acid (KA). **a** Drugs or VEH were administered i.p. in Swiss Albino mice. 30 min later, KA (40 mg/kg, i.p.) was administered. Immediately after KA administration, the mice were subjected to an evaluation of behavioral seizures for 2 h. **b**–**g** The doses of drugs are shown on x-axes in mg/kg. Data are expressed as means ± SEM of seizure severity or latency to scratching and the percentage of animals displaying stage 7 characteristics (SE/death). Latency to scratching was impossible to measure in mice that received 8 mg Zn/kg because they proceeded immediately to advanced seizure stages. **b**–**d** n = 10 VEH, n = 10 TC-G 1008 10, n = 9 TC-G 1008 20, n = 8 TC-G 1008 40. **e**–**g** n = 10 VEH, n = 10 Zn 2, n = 10 Zn 4, n = 12 Zn 8. p values were determined by one-way ANOVA and Bonferroni’s multiple comparison test or the Chi-square test. *p < 0.05, **p < 0.01

Because GPR39 KO mice were previously characterized by increased susceptibility to acute seizures induced by the chemoconvulsant KA [21], our next step was to investigate whether single doses of the GPR39 agonists (TC-G 1008 and ZnCl_2_) influence behavioral seizures induced by acute KA (40 mg/kg, i.p.) administration in mice (Fig. 4a). A one-way ANOVA showed no effect of TC-G 1008 [F(3,36) = 0.8683, p = 0.4673] on the maximal seizure score in response to KA (40 mg/kg) (Fig. 4b) but a significant effect of ZnCl_2_ [F(3,38) = 5.549, p = 0.0029] on this parameter was observed. ZnCl_2_ (8 mg Zn/kg) significantly increased the maximal seizure score (Fig. 4e). Furthermore, one-way ANOVA demonstrated a significant effect of both TC-G 1008 [F(3,24) = 5.549, p = 0.0029] and ZnCl_2_ [F(2,20) = 7.01, p = 0.0049] on the latency to scratching (stage 2 seizures). TC-G 1008 at doses of 10, 20, and 40 mg/kg (Fig. 4c) and ZnCl_2_ at doses of 2 and 4 mg Zn/kg (Fig. 3f) significantly decreased the latency to scratching. This parameter was impossible to measure after administration of the 8 mg Zn/kg dose because mice proceeded immediately to advanced seizure stages. The incidence of SE/death (stage 7 seizures) was 40%, 33%, and 50% after TC-G 1008 administration at doses of 10, 20, and 40 mg/kg and KA (40 mg/kg), respectively (Chi-square test, p = 0.0951) (Fig. 4d). In the case of ZnCl_2_, the incidence of SE/death was 20%, 40%, and 67% following 2, 4, and 8 mg Zn/kg and KA (40 mg/kg), respectively (Chi-square test, p = 0.0074) (Fig. 4g). Thus, ZnCl_2_ significantly increased the incidence of SE/death in response to KA. None of the control mice injected with KA (40 mg/kg) exhibited SE/death. Overall, these data showed worsening of acute KA-induced seizures after administering GPR39 agonists (TC-G 1008 or ZnCl_2_).

### TC-G 1008 increases the mean duration of epileptiform-like events in zebrafish larvae

Then, we assessed whether single doses of GPR39 agonists (TC-G 1008 and ZnCl_2_) influence behavioral seizures induced by the chemoconvulsant PTZ. The effects of TC-G 1008 and ZnCl_2_ on the seizure threshold induced by PTZ in mice are shown in the Supplementary file (Fig. S1). Except the high dose of ZnCl_2_ (32 mg Zn/kg) that decreased the threshold for forelimb tonus, TC-G 1008 or ZnCl_2_ did not significantly affect the behavioral parameters measured in the i.v. PTZ-seizure threshold test.

**Fig. 5:**
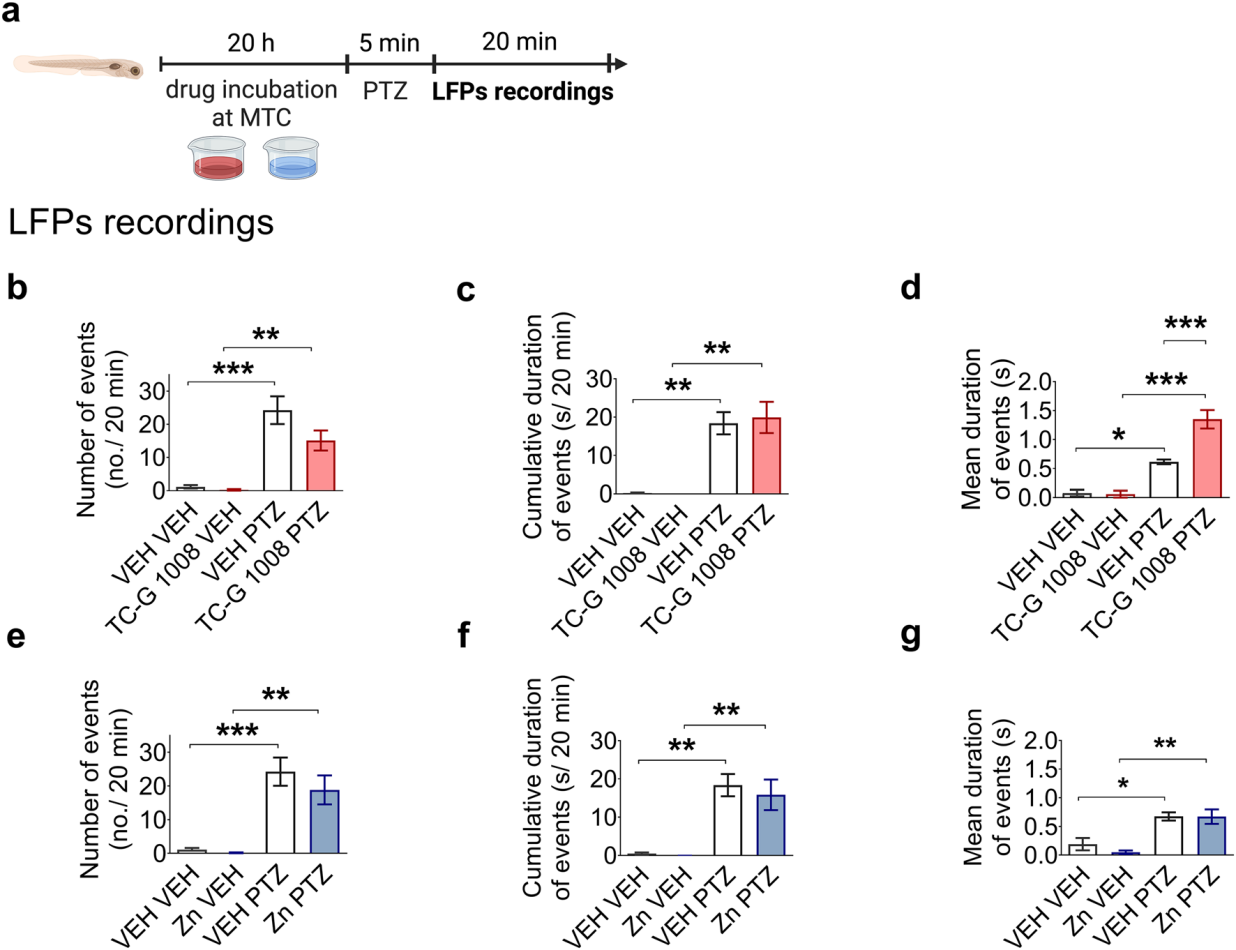
Local field potential (LFP) recordings from the optic tectum of zebrafish larvae exposed to TC-G 1008 (**b**–**d**) or ZnCl_2_ (**e**–**g**) and pentylenetetrazole (PTZ). **a** Zebrafish larvae were incubated with TC-G 1008 (70 µM), ZnCl_2_ (65 µM) (at maximally tolerated concentrations, MTCs) or VEH for 20 h and subsequently exposed to PTZ (20 mM) or VEH for 5 min. The LFP recordings began 5 min after removing the larva from VEH/PTZ solution and lasted 20 min. **b**–**g** Results are expressed as means ± SEM of the number of epileptiform-like events, the cumulative duration of epileptiform-like events, and the mean duration of epileptiform-like events. **b**–**d** n = 10 VEH VEH, n = 8 VEH PTZ, n = 5 TC-G 1008 VEH, n = 14 TC-G 1008 PTZ. **e–g** n = 10 VEH VEH, n = 8 VEH PTZ, n = 7 Zn VEH, n = 17 Zn PTZ. p values were determined by one-way ANOVA and Bonferroni’s multiple comparison test. *p < 0.05, **p < 0.01, ***p < 0.001

However, some of the approved anti-seizure drugs are inactive in acute behavioral tests, but their activity can be detected by electrographic methods such as EEG or LFP [48, 49]. Therefore, we examined the effects of the compounds under study on electrographic seizures induced by PTZ. To characterize the effects of GPR39 agonists (TC-G 1008 and ZnCl_2_) on electrographic seizures, we utilized the model of acute seizures induced in zebrafish larvae by PTZ (Fig. 5a). This model is a drug screening platform that allows one to monitor the brain’s electrical activity by measuring LFPs. Application of PTZ produces ictal-like electrographic discharges on LFP recordings, which can be suppressed by anti-seizure drugs [48, 49].

A two-way ANOVA showed a significant effect of TC-G 1008 on the mean duration of epileptiform-like events [F(1,30) = 5.385, p = 0.0273], a significant effect of PTZ [F(1,30) = 34.79, p < 0.0001] and a significant TC-G 1008 × PTZ interaction [F(1,30) = 5.965, p = 0.0207] (Fig. 5d). There was also a significant effect of PTZ [F(1,33) = 35.88, p < 0.0001], no effect of TC-G 1008 [F(1,33) = 2.522, p = 0.1218] and no PTZ × TC-G 1008 interaction [F(1,33) = 1.702, p = 0.2011] on the number of epileptiform-like events (Fig. 5b). Similarly, there was a significant effect of PTZ [F(1,31) = 22.88, p < 0.0001], no effect of TC-G 1008 [F(1,31) = 0.02596, p = 0.8264] and no PTZ × TC-G 1008 interaction [F(1,31) = 0.04892, p = 0.8264] on the cumulative duration of epileptiform-like events (Fig. 5c). Zebrafish larvae exposed to PTZ (20 mM) displayed significantly increased number of epileptiform-like events, the cumulative duration of epileptiform-like events and the mean duration of epileptiform-like events, compared to VEH (Fig. 5b–g), thus showing that the chemoconvulsant induced electrographic discharges on LFPs and the model was executed successfully. Administration of TC-G 1008 (70 µM) did not affect the parameters examined in larvae treated with VEH instead of PTZ (Fig. 5b–d), thus demonstrating that the compound per se did not produce electrographic discharges. TC-G 1008 did not affect the number of events (Fig. 5b) or the cumulative duration of events (Fig. 5c) in larvae exposed to PTZ but increased the mean duration of epileptiform-like events (Fig. 5d). These data indicated that TC-G 1008 worsened electrographic seizures induced by PTZ in zebrafish larvae.

Furthermore, a two-way ANOVA demonstrated a significant effect of PTZ [F(1,38) = 25.81, p < 0.0001], no effect of ZnCl_2_ [F(1,38) = 0.6019, p = 0.4427] and no PTZ × ZnCl_2_ interaction [F(1,38) = 0.2952, p = 0.5901] on the number of epileptiform-like events (Fig. 5e). There was also a significant effect of PTZ [F(1,37) = 20, p < 0.0001], no effect of ZnCl_2_ [F(1,37) = 0.156, p = 0.6951] and PTZ × ZnCl_2_ interaction [F(1,37) = 0.07757, p = 0.7822] on the cumulative duration of events (Fig. 5f). Finally, there was a significant effect of PTZ [F(1,38) = 20, p < 0.0001], no effect of ZnCl_2_ [F(1,38) = 0.3433, p = 0.5614] and no PTZ × ZnCl_2_ interaction [F(1,38) = 0.3154, p = 0.5777] on the mean duration of epileptiform-like events (Fig. 5g). Administration of ZnCl_2_ (65 µM) in larvae exposed to PTZ did not affect the number of epileptiform-like events (Fig. 5e), the cumulative duration of events (Fig. 5f), or the mean duration of such events (Fig. 5g), compared to VEH. These results showed that ZnCl_2_ did not affect electrographic seizures induced by PTZ in zebrafish larvae.

### TC-G 1008 facilitates the development of PTZ-induced epileptogenesis in mice

**Fig. 6:**
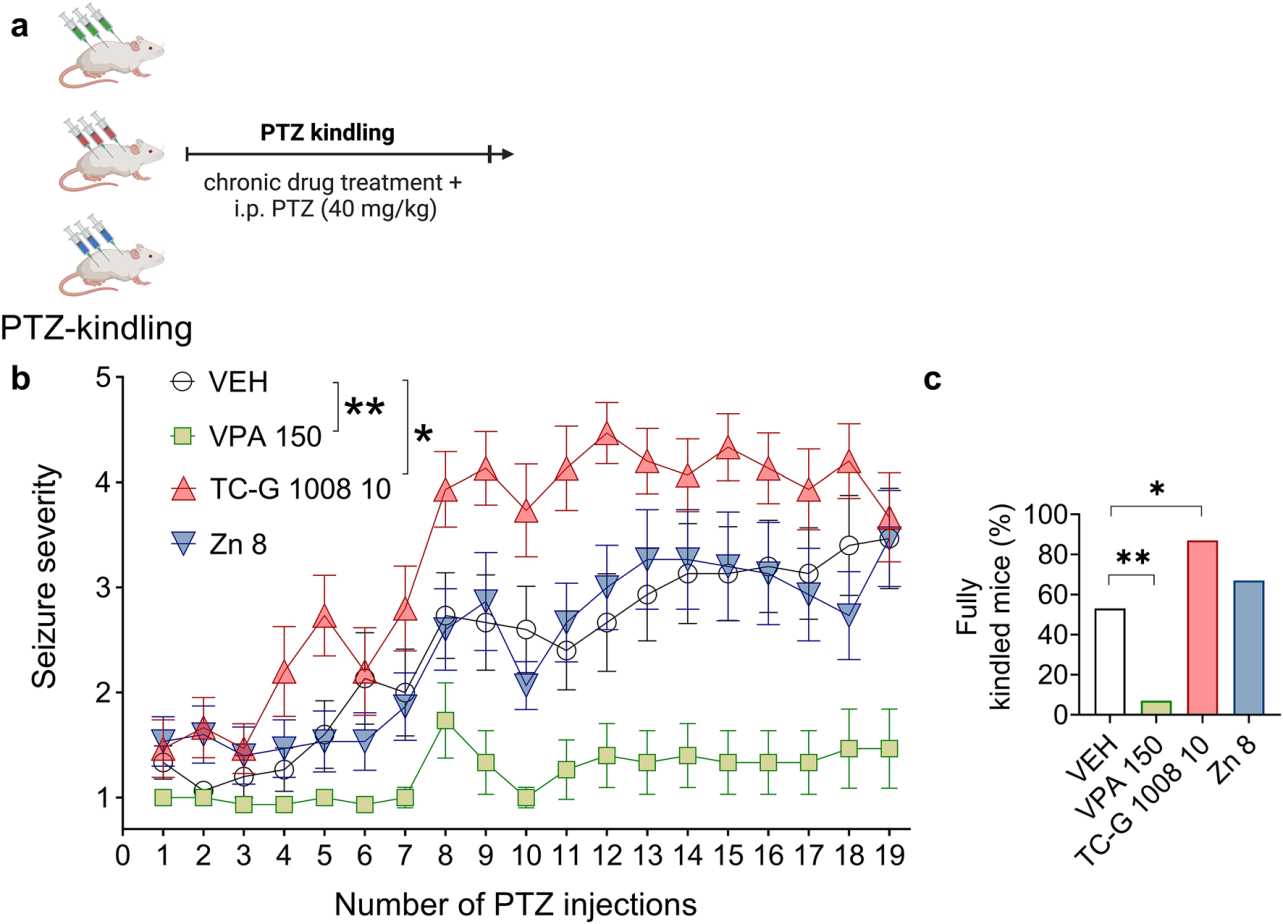
The effects of chronic treatment with VPA, TC-G 1008, or ZnCl_2_ on PTZ-induced epileptogenesis in Swiss Albino mice. **a** VPA (150 mg/kg), TC-G 1008 (10 mg/kg), ZnCl_2_ (8 mg Zn/kg), or VEH were injected i.p. once daily on alternate days during weekdays. 30 min later, PTZ (40 mg/kg) was injected i.p. Immediately after each PTZ injection, the mice were subjected to an evaluation of behavioral seizures, which lasted 30 min. The total number of PTZ injections was 19. Data are expressed as means ± SEM of seizure severity after each injection (**b**) and the percentage of mice displaying consecutive stage 5 seizures (fully kindled mice) after the last PTZ injection (**c**). n = 15 in each group. p values were determined by two-way repeated measures ANOVA and Dunnett’s multiple comparison test or Fisher’s exact test. *p < 0.05, **p < 0.01

Because very often the effects observed after one-time administration of compounds differ from the effects induced by repeated administration [53], we next examined the effects of chronic treatment with GPR39 agonists (TC-G 1008 and ZnCl_2_) on the process of epileptogenesis. To test the effects on epileptogenesis, we utilized the PTZ model of chemical kindling in mice (Fig. 6a). A two-way repeated measures ANOVA showed a significant effect of time [F(3.291, 184.3) = 25.80; p < 0.0001], a significant effect of drug [F(3,56) = 13.38, p < 0.0001], and a significant drug × time interaction [F(54,1008) = 2.546; p < 0.0001] on seizure severity. VPA (150 mg/kg) decreased the maximal seizure severity compared to VEH (p = 0.002, Dunnett’s multiple comparison test). In contrast, TC-G 1008 (10 mg/kg) increased the maximal seizure severity compared to VEH (p = 0.02, Dunnett’s multiple comparison test), while ZnCl_2_ did not significantly affect this parameter (Fig. 6b). After 19 injections of PTZ (40 mg/kg), the percentage of fully kindled Swiss albino mice, experiencing consecutive generalized clonic seizures with loss of righting reflex (stage 5 seizures), was 6.7% of mice treated with VPA (150 mg/kg), 53% of mice treated with VEH, 67% of mice treated with ZnCl_2_ (8 mg Zn/kg) and 87% of mice treated with TC-G 1008 (10 mg/kg) (Fisher’s exact test p = 0.01 in the case of VPA 150, p = 0.71 in the case of Zn 8, p = 0.04 in the case of TC-G 1008 10). Hence, TC-G 1008 significantly increased the percentage of fully kindled mice, while VPA produced the opposite effect on this parameter (Fig. 6c). To reduce possible mortality in mice treated with TC-G 1008 (10 mg/kg), exhibiting consecutive stage 5 seizures, kindling was terminated after 19 injections of PTZ (40 mg/kg). Overall, chronic treatment with the three drugs produced distinct effects in the model: VPA suppressed epileptogenesis, TC-G 1008 facilitated epileptogenesis, and ZnCl_2_ had no effect on epileptogenesis.

The effects of chronic administration of the examined compounds in kindled and non-kindled mice on neuromuscular strength and motor coordination are shown in Table S3. After 19 injections, none of the drugs significantly affected the neuromuscular strength or motor coordination in non-kindled mice. Similarly, after 19 injections, none of the compounds significantly affected the outcomes of these tests in mice kindled with PTZ (40 mg/kg).

In summary, the effects of the small molecule agonist at the GPR39 receptor, TC-G 1008, in acute and chronic models of seizures and epilepsy, examined via behavioral and electrographic approaches, are the following: the molecule decreased the seizure threshold in the MEST test, worsened behavioral seizures in response to KA, and worsened electrographic seizures in response to PTZ in zebrafish larvae as well as aggravated PTZ-epileptogenesis.

### GPR39 KO mice do not differ from WT mice in terms of the seizure threshold in the MEST test

**Fig. 7:**
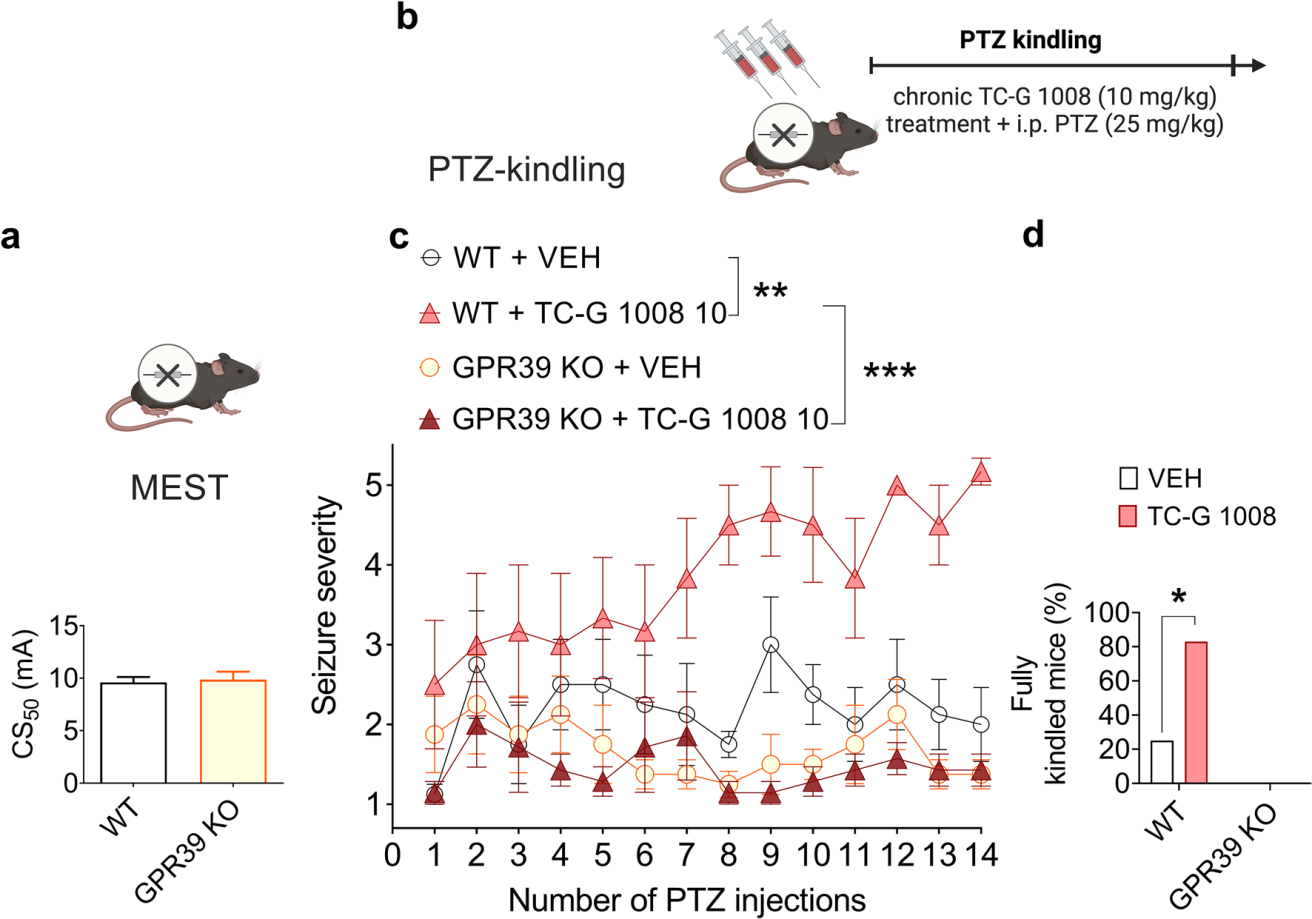
The effects of GPR39 KO in C57BL/6/Tar × CBA/Tar mice on seizure threshold in the MEST test (**a**) and on PTZ-induced epileptogenesis (**b**–**d**). **a** WT and GPR39 KO mice were subjected to the MEST test. Data are expressed as CS_50_ (in mA) with upper 95% confidence limits. n = 20 in each group. P value was determined by the Student’s t-test (**b**) WT and GPR39 KO mice were injected i.p. once daily with TC-G 1008 (10 mg/kg) or VEH on every alternate day during weekdays. 30 min later, the mice were injected i.p. with PTZ (25 mg/kg). Immediately after each PTZ injection, the mice were subjected to an evaluation of behavioral seizures, which lasted 30 min. The total number of PTZ injections was 14. Data are expressed as means ± SEM of seizure severity after each injection (**c**) and the percentage of mice displaying consecutive stage 5 seizures after the last PTZ injection (**d**). One outlier was excluded in the WT TC-G 1008 group leading to n** = **8 WT VEH, n = 6 WT TC-G 1008, n = 8 KO VEH, n = 7 KO TC-G 1008. p values were determined by three-way repeated measures ANOVA and Bonferroni’s multiple comparison test or Fisher’s exact test. *p < 0.05, **p < 0.01, ***p < 0.001

Our next aim was to explore the role of the GPR39 receptor in seizure behavior and epileptogenesis using the KO mice model. The GPR39 KO mice model was generated in a mixed genetic background (C57BL/6/Tar × CBA/Tar). GPR39 KO and WT mice were subjected to the MEST test. The seizure threshold did not differ between the GPR39 KO and WT mice in this test (t(18) = 1.19, p = 0.2505, Student’s t-test) (Fig. 7a). Also, the neuromuscular strength or motor coordination did not differ significantly between GPR39 KO and WT mice (Table S4).

### TC-G 1008 facilitates the development of PTZ-induced epileptogenesis in WT but not GPR39 KO mice

To examine the role of the GPR39 receptor in epileptogenesis, GPR39 KO, and WT mice were subjected to the PTZ-induced kindling model (Fig. 7b). A three-way repeated measures ANOVA showed a significant effect of genotype [F(1,25) = 24.6011, p = 0.0000]; a significant effect of drug [F(1,25) = 6.1898, p = 0.0199]; a significant genotype × drug interaction [F(1,25) = 10.2347, p = 0.0037]; a significant effect of time [F(13,325) = 1.7759, p = 0.0457], and a significant time × genotype interaction [F(13,325) = 2.6197, p = 0.0018] on seizure severity. The maximal seizure severity did not differ between the GPR39 KO and WT mice treated with VEH. However, chronic administration of TC-G 1008 (10 mg/kg) increased the maximal seizure score in WT mice (p = 0.0035, Bonferroni’s multiple comparison test). This effect was not observed in GPR39 KO mice (Fig. 7c). These data showed that TC-G 1008 facilitated the development of PTZ-epileptogenesis by acting selectively at the GPR39 receptor. After 14 injections of PTZ (25 mg/kg), the percentage of fully kindled mice was 25% in the WT VEH group, 83.3% in the WT TC-G 1008 group, 0% in the KO VEH, and 0% in the KO TC-G 1008 group. Fisher’s exact test p = 0.02 regarding WT TC-G 1008 group indicated that TC-G 1008 significantly increased the percentage of fully kindled WT mice (Fig. 7d). Furthermore, the percentage of fully kindled WT (C57BL/6/Tar × CBA/Tar) mice that were administered TC-G 1008 (10 mg/kg) was similar (> 80%) to that of fully kindled Swiss Albino mice (Fig. 6c) subjected to treatment with this compound at the end of both kindling procedures. Thus, TC-G 1008 enhanced epileptogenesis in two strains of mice (Swiss Albino and C57BL/6/Tar × CBA/Tar). To reduce possible mortality in C57BL/6/Tar × CBA/Tar mice treated with TC-G 1008 (10 mg/kg), exhibiting consecutive stage 5 seizures, kindling was terminated after 14 injections of PTZ (25 mg/kg). Surprisingly, after 14 injections of TC-G 1008, neuromuscular strength increased in GPR39 KO mice subjected to PTZ-induced (25 mg/kg) kindling but not in WT mice (Table S5).

### GPR39 KO mice subjected to the PTZ-kindling model display decreased total serum zinc concentration but no significant alterations in total zinc in the hippocampus

**Fig. 8:**
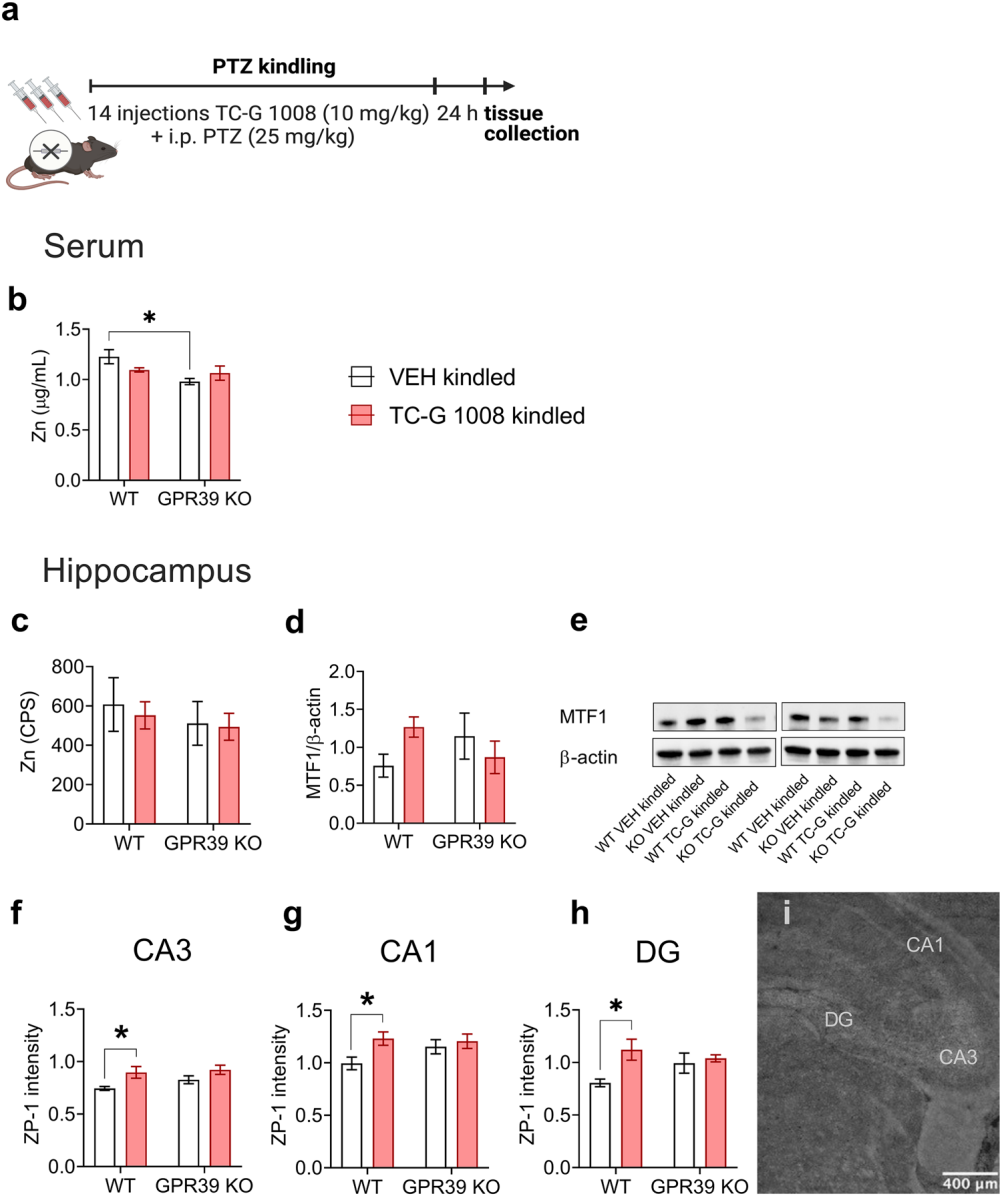
The effects of PTZ-kindling in GPR39 KO or WT mice and chronic treatment with TC-G 1008 (10 mg/kg) on zinc levels in serum and hippocampus. **a** The PTZ kindling in GPR39 KO or WT (C57BL/6/Tar × CBA/Tar) mice consisted of 14 injections of PTZ (25 mg/kg). The biochemical analyses were performed 24 h after the last PTZ injection. **b** Total zinc (protein bound and [Zn^2+^]_I_) was measured in serum by ICP-OES. Data are expressed as means ± SEM. n** = **8 WT VEH, n = 6 WT TC-G 1008, n = 7 KO VEH, n = 7 KO TC-G 1008. **c** Total zinc was analyzed semi-quantitatively in coronal hippocampal sections by LA-ICP-MS. Data are expressed as means ± SEM of counts per second (CPS). n = 4 in each group. **d** The protein expression level of the putative [Zn^2+^]_I_ sensor, metal regulatory transcription factor 1 (MTF1), was analyzed in the hippocampus. The results (means ± SEM) are presented as the MTF1/β-actin ratio. n = 7 in each group. **e** Representative blots of MTF1. The observed band (~ 130 kDa). **f–h** [Zn^2+^]_I_ was examined in coronal hippocampal sections by a cell-membrane permeable probe, Zinpyr-1 (ZP-1). The mean ZP-1 grey values ratio between mouse sections in the CA3, CA1, or dentate gyrus (DG) regions of the hippocampus is shown. n = 6 WT VEH, n = 6 WT TC-G 1008, n = 5 KO VEH, n = 6 KO TC-G 1008. **i** A greyscale image of the hippocampal section after staining with ZP-1. p values were determined by two-way ANOVA and Bonferroni’s multiple comparison test. *p < 0.05

We were interested in the possible molecular mechanisms involved in the observed behavioral effects. Because GPR39 is the presumed zinc receptor, we initially focused on the biochemical analyses of serum and hippocampal zinc. We focused on the hippocampus because there is a strong link between the hippocampus and epilepsy [54], and it contains the highest zinc levels [55]. The available methods for assessing zinc concentration include the analysis of total elemental zinc (ions bound to proteins and free zinc ions) and free ions not bound to proteins [56]. Total and free zinc pools have been suggested to play a role in the pathophysiology of epilepsy [57, 58]. We analyzed the total zinc levels in sera and hippocampi of GPR39 KO and WT C57BL/6/Tar × CBA/Tar mice subjected to the PTZ-kindling model and treatment with TC-G 1008 (Fig. 8a). 24 h after the completion of the kindling paradigm, a two-way ANOVA showed a significant effect of genotype [F(1,24) = 6.00, p = 0.0220], a borderline interaction between genotype × drug [F(1,24) = 3.56, p = 0.0713] and no effect of drug [F(1,24) = 0.17, p = 0.6813]. Total serum zinc concentration was lower in VEH-treated GPR39 KO mice subjected to PTZ-kindling compared with VEH-treated WT mice. Chronic administration of TC-G 1008 did not affect total serum zinc concentration in either GPR39 KO or WT mice subjected to this paradigm (Fig. 8b).

Semi-quantitative analysis using LA-ICP-MS applied to hippocampal slices did not reveal statistically significant differences in total zinc in the hippocampus. There was no genotype effect [F(1,12) = 0.5914, p = 0.4567], no drug effect [F(1,12) = 0.1320, p = 0.7227], or genotype × drug interaction [F(1,12) = 0.03578, p = 0.8531] (Fig. 8c). Hence, the changes in total zinc concentration observed in the sera of GPR39 KO mice subjected to PTZ-kindling were not accompanied by changes in total zinc in hippocampal tissue.

### Chronic treatment with TC-G 1008 increases [Zn^2+^]_I_ in the hippocampus of WT mice subjected to the PTZ-kindling model

Free zinc ions, found both in the extracellular and intracellular space ([Zn^2+^]_I_), are more easily exchangeable than total zinc and participate in cellular signaling [56]. In serum, free zinc concentration did not correlate with total concentration measured by ICP-MS [59]. Therefore, we were interested whether there were alterations in [Zn^2+^]_I_ in the hippocampus at a time point when total zinc did not change. [Zn^2+^]_I_ can be detected by fluorescent probes [56] and activates the transcription factor MTF1. The latter is a candidate zinc sensor [60] that supposedly mediates changes in [Zn^2+^]_I_ in the hippocampus of patients with treatment-resistant epilepsy [57]. 24 h after the completion of the kindling paradigm, there was a borderline interaction between genotype × drug [F(1,24) = 3.738, p = 0.0651], no effect of genotype [F(1,24) = 0.0037, p = 0.9519] and no effect of drug [F(1,24) = 0.2655, p = 0.6111] on the expression of MTF1 protein. VEH-treated GPR39 KO mice did not differ from VEH-treated WT mice in MTF1 expression. WT mice treated with TC-G 1008 during the kindling paradigm displayed increased (by 86%) expression of MTF1 protein in the hippocampus (Fig. 8d). The latter result was not statistically significant but indicative of increased [Zn^2+^]_I_ in these mice.

To further analyze [Zn^2+^]_I_, we applied a cell-membrane permeable probe, ZP-1, to hippocampal slices. 24 h after the completion of the kindling paradigm, there was a significant effect of the drug on ZP-1 intensity in all analyzed regions of the hippocampus, i.e., CA3 [F(1,19) = 8.88, p = 0.0077], CA1 [F(1,19) = 4.91, p = 0.0392] and DG [F(1,19) = 6.41, p = 0.0203]. ZP-1 intensity did not differ significantly between hippocampal sections from VEH-treated GPR39 KO and WT mice in the CA3, CA1, or DG regions (Fig. 8f–h). However, chronic treatment with TC-G 1008 increased ZP-1 intensity in the CA3 (Fig. 8f), CA1 (Fig. 8g), and DG (Fig. 8h) regions of the hippocampus of WT mice subjected to the PTZ-kindling model of epilepsy. Altogether, the analysis of total and free zinc revealed that GPR39 KO mice displayed decreased total serum zinc but no alterations in total zinc the hippocampus in this model. WT mice treated with TC-G 1008 during the kindling paradigm displayed increased [Zn^2+^]_I_ in the hippocampus.

The effects of acute and chronic treatments with TC-G 1008, ZnCl_2_, or VPA and models of acute seizures (MES, 6-Hz) or the chronic PTZ-kindling model on total serum zinc and hippocampal [Zn^2+^]_I_ in genetically unmodified (Swiss Albino mice) are shown in the Supplementary file (Figs. S2–S3, S5–S6, S8–S9).

### Chronic treatment with TC-G 1008 markedly increases activation of CREB in the hippocampus of GPR39 KO mice subjected to the PTZ-kindling model

Next, we examined the expression of GPR39 signaling pathway proteins. The G_q_ and G_s_ pathways activated by TC-G 1008 [28, 29] converge on the transcription factor CREB. CREB increases the transcription of the neurotrophin BDNF, which signals via the TrkB receptor [61]. The mouse hippocampal cell line (HT-22) treated with TC-G 1008 displayed increased expression of CREB and BDNF proteins [62], while a single dose of TC-G 1008 increased the expression of BDNF protein level in the hippocampus [63]. BDNF signaling through its high-affinity TrkB receptor is involved in epileptogenesis [64]. Based on these data, we analyzed CREB and TrkB activation and BDNF protein levels in the hippocampi of GPR39 KO and WT C57BL/6/Tar × CBA/Tar mice subjected to the PTZ-kindling model and treatment with TC-G 1008.

**Fig. 9:**
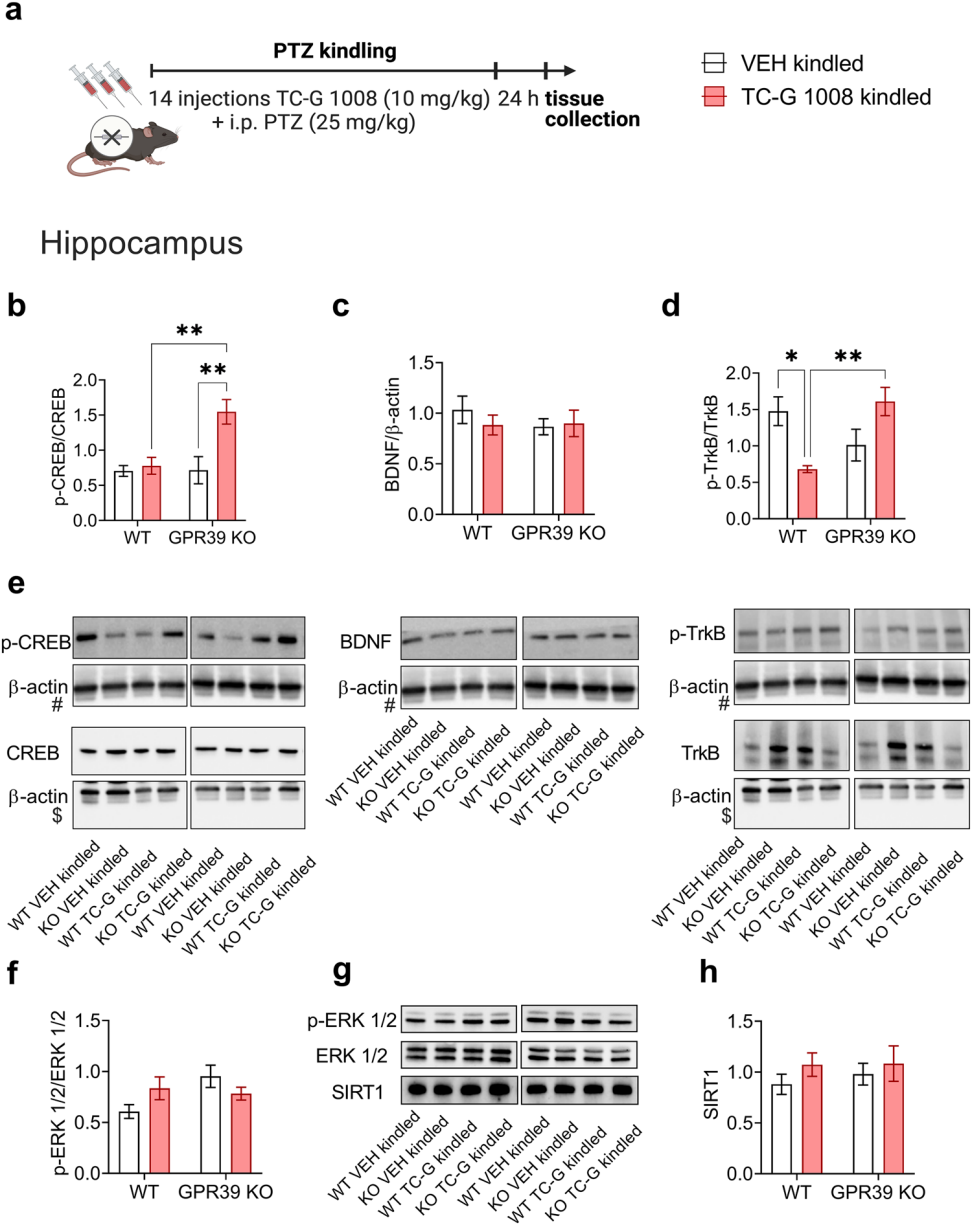
The effects of PTZ-kindling in GPR39 KO or WT (C57BL/6/Tar × CBA/Tar) mice and chronic treatment with TC-G 1008 (10 mg/kg) on the expression of proteins in the GPR39 signaling pathway in the hippocampus. **a** The PTZ kindling in GPR39 KO or WT (C57BL/6/Tar × CBA/Tar) mice consisted of 14 injections of PTZ (25 mg/kg). The biochemical analyses were performed 24 h after the last PTZ injection. **b**–**d** The expression of p-CREB, CREB, BDNF, p-TrkB, and TrkB was normalized to β-actin. The results (means ± SEM) are shown as the p-CREB/CREB, BDNF/β-actin, or p-TrkB/TrkB. **b** n = 7 in each group. **c**–**d** n = 6 in each group. **e** Representative blots of p-CREB, CREB (~ 46 kDa), BDNF (~ 14 kDa), p-TrkB, TrkB (~ 140 kDa), and β-actin (~ 42 kDa). ^#^p-CREB, BDNF, and p-TrkB come from the same blot; ^$^CREB and TrkB come from the same blot, thus sharing the corresponding β-actin band. **f**, **h** The expression p-ERK1/2, ERK1/2, and SIRT1 was normalized to the total protein concentration. **f** The results (means ± SEM) are shown as p-ERK1/2/ERK1/2. n = 6 WT VEH, n = 6 WT TC-G 1008, n = 7 KO VEH, n = 7 KO TC-G 1008. **h** n = 7 WT VEH, n = 6 WT TC-G 1008, n = 5 KO VEH, n = 6 KO TC-G 100. **g** Representative blots of p-ERK1/2, ERK1/2 (~ 44, 42 kDa) and SIRT1 (the observed band ~ 130 kDa). p values were determined by two-way ANOVA and Bonferroni’s multiple comparison test. *p < 0.05, **p < 0.01

24 h after the completion of the kindling paradigm, there was a significant effect of genotype [F(1,24) = 6.87, p = 0.015], a significant effect of drug [F(1,24) = 6.87, p = 0.015] and a significant genotype × drug interaction [F(1,24) = 6.518, p = 0.0175] on CREB activation in the hippocampus, measured as the ratio of p-CREB/total CREB protein expression (Fig. 9b). VEH-treated GPR39 KO and WT mice subjected to the PTZ-kindling model did not differ significantly in p-CREB/CREB. Surprisingly, chronic administration of TC-G 1008 markedly and significantly increased the expression of the p-CREB protein (by 137%) and the p-CREB/CREB ratio (by 115%) in the hippocampus of GPR39 KO mice but not in WT mice (Fig. 9b). These data suggest the non-selective activity of TC-G 1008 on CREB upon activation of the GPR39 receptor.

There was no effect of genotype [F(1,20) = 0.44, p = 0.5166], no effect of drug [F(1,20) = 0.26, p = 0.6131] or genotype × drug interaction [F(1,20) = 0.65, p = 0.4288] on the expression of BDNF protein. VEH-treated GPR39 KO and WT mice subjected to the PTZ-kindling model did not differ significantly in BDNF protein levels in the hippocampus. Chronic treatment with TC-G 1008 did not significantly affect BDNF protein levels in the hippocampus of either WT or KO mice (Fig. 9c).

There was a significant interaction between genotype × drug [F(1,20) = 15.35, p = 0.0009] but no effect of genotype [F(1,20) = 1.698, p = 0.2074] or drug [F(1,20) = 0.3079, p = 0.5851] on TrkB activation in the hippocampus, measured as the ratio of p-TrkB/total TrkB protein expression. VEH-treated GPR39 KO and WT mice subjected to the PTZ-kindling model did not differ significantly in p-TrkB/TrkB in the hippocampus. The p-TrkB/TrkB ratio increased by 59% in the hippocampus of GPR39 KO treated with TC-G 1008 (the result was insignificant). Furthermore, chronic treatment with TC-G 1008 significantly decreased the p-TrkB/TrkB ratio (by 54%) in the hippocampus of WT mice (Fig. 9d). Altogether, the effects of TC-G 1008 on CREB and TrkB activation in GPR39 KO mice indicate the non-selective effects of this small molecule agonist. They also suggest the occurrence of mechanisms preventing such activation in WT mice in this model.

The effects of acute and chronic treatments with TC-G 1008, ZnCl_2_, or VPA and models of acute seizures (MES, 6-Hz) or the chronic PTZ-kindling model on the expression of p-CREB/CREB, BDNF, and p-TrkB/TrkB in the hippocampus in genetically unmodified (Swiss Albino mice) are shown in the Supplementary file (Figs. S4, S7, and S10).

### Chronic treatment with TC-G 1008 does not significantly affect the activation of ERK1/2 or SIRT1 in the hippocampus of GPR39 KO or WT mice subjected to the PTZ-kindling model

We also assessed ERK1/2 kinase activation in the hippocampi of GPR39 KO and WT C57BL/6/Tar × CBA/Tar mice subjected to the PTZ-kindling model and treatment with TC-G 1008. The metabotropic effects of zinc are followed by the phosphorylation of ERK1/2 in the CA3 region of the hippocampus [65]. In the keratinocyte cell line (HaCaT), TC-G 1008 induced a marked increase in ERK1/2 kinase activation, measured by the ratio of p-EKR1/2 to ERK1/2, which was independent of G_q_-phospholipase C (PLC), G_s_-protein kinase A (PKA) and β-arrestin [66]. 24 h after the completion of the kindling paradigm, there was a significant interaction between genotype × drug [F(1,22) = 4.778, p = 0.0398], no effect of genotype [F(1,22) = 2.604, p = 0.1209] and no effect of drug [F(1,22) = 0.1025, P = 0.7519] on EKR1/2 activation in the hippocampus. The ratio of p-EKR1/2 to ERK1/2 increased (by 58%) in the hippocampus of VEH-treated GPR39 KO mice subjected to PTZ-kindling, compared with VEH-treated WT mice (the result was insignificant)). Chronic administration of TC-G 1008 did not significantly affect the ratio of p-EKR1/2 to ERK1/2 in the hippocampus of either WT or GPR39 KO mice that underwent PTZ-kindling. (Fig. 9f). These results suggest that ERK1/2 is not involved in either the selective or non-selective effects of TC-G 1008 on GPR39 in this model.

Finally, we measured SIRT1 expression in the hippocampi of GPR39 KO and WT C57BL/6/Tar × CBA/Tar mice subjected to the PTZ-kindling model and treatment with TC-G 1008. TC-G 1008 up-regulated the expression of SIRT1 in the brain of rat pup in a model of brain injury induced by hypoxia–ischemia [19]. Here, 24 h after the completion of the kindling paradigm, there was no effect of genotype [F(1,20) = 0.1832; p = 0.6732], no effect of drug [F(1,20) = 1.333, p = 0.2619] and no interaction between genotype and drug [F(1,20) = 1.333, p = 0.2619] on the expression of SIRT1 protein in the hippocampus. These results suggest that SIRT1 is not involved in either the selective or non-selective effects of TC-G 1008 on GPR39 in the PTZ-kindling model.

## Discussion and conclusions

Two main findings arose from this study. First, TC-G 1008, a small molecule agonist at the GPR39 receptor, aggravated epileptogenesis in an animal model of epilepsy by acting selectively at GPR39. Second, TC-G 1008 exhibited non-selective activity by activating CREB in the hippocampus.

As one of the first pharmacological tool compounds for GPR39, TC-G 1008 has been used extensively to characterize the function of the receptor [34], but no study to date has demonstrated its in vivo mediation of TC-G 1008 effects. TC-G 1008 was initially described as selective for the GPR39 receptor [28], but another study showed that it might bind to the serotonin 5-HT_1A_ receptor [29]. Although the possibility of TC-G 1008 acting at another target has been suggested [29, 67], conclusions inferred from these investigations focused heavily on the effects of GPR39 activation and its therapeutic potential. Our study demonstrated the selective and non-selective effects of TC-G 1008, evidenced by its impact on behavioral seizures in the PTZ-kindling model and the activation of hippocampal CREB, respectively. Therefore, there is a need to determine if some specific effects of TC-G 1008 are GPR39-dependent.

We found no impact of the GPR39 gene on either acute seizures induced by an electrical stimulus (MES) or epileptogenesis. However, the effects of TC-G 1008 in the chronic, PTZ-induced kindling model of epilepsy in WT (C57BL/6/Tar × CBA/Tar) mice were similar to those observed in Swiss Albino mice, while no effects were seen in GPR39 KO (C57BL/6/Tar × CBA/Tar) mice. We were thus able to demonstrate that TC-G 1008 facilitated PTZ-induced epileptogenesis via the GPR39 receptor. Our data showed that activation of GPR39 aggravated epileptogenesis. The data argue against GPR39 activation being a potential therapeutic strategy for treating epilepsy, and hence contrary to the initially proposed hypothesis. We conjectured the anti-seizure/antiepileptogenic effects of GPR39 receptor activation based on a previous in vivo study by Gilad et al. [21] in which GPR39 KO mice displayed higher susceptibility to acute seizures from a single i.p. dose of KA. Also, in vitro data have suggested therapeutic effects of GPR39 activation on seizures based on the observation that activation of GPR39 by extracellular zinc up-regulated the potassium-chloride co-transporter 2 (KCC2) [20, 21], which is necessary for the inhibitory function of the GABA_A_ receptor [68] and inhibited the release of the excitatory neurotransmitter, glutamate [69].

The activation of GPR39 and the concomitant facilitation of epileptogenesis may have important therapeutic implications. Drug discovery efforts are focused on GPR39 [70–73], and studies suggest a novel pharmacological strategy of agonism [34]. It is noteworthy that gandotinib (LY2784544) and GSK2636771, a potent, selective inhibitor of PI3Kβ used in clinical trials for cancer, are agonists [29]. Furthermore, a screen of repurposed drugs found gandotinib as a potential contraceptive [74]. On the other hand, antagonism was recently suggested as beneficial in the treatment of myocardial infarction [14] or cardiac hypertrophy [15]. Enhanced expression of GPR39 has been documented in the aging human brain [17] and cancers [75]. The current data point to the complex role of the GPR39 receptor as a target for pharmacological intervention. Careful consideration whether agonists or antagonists are drug leads is necessary. Worsening cases of epilepsies may be related to the side effects of newer drugs, which are GPR39 agonists.

The study by Gilad et al. [21] on the susceptibility to acute seizures induced by KA utilized GPR39 KO mice bred on a C57BL/6 genetic background. In our study, our GPR39 KO model was established in a mixed genetic background (C57BL/6/Tar × CBA/Tar). The genetic background may account for seizure susceptibility [76]. The dose of KA (10 mg/kg) used in the Gilad et al. study [21] produced stage 5 seizures (loss of posture or SE) in 27% of the WT mice. Because we hypothesized that the GPR39 agonist would protect mice from seizures induced by KA, we examined the effects of TC-G 1008 and ZnCl_2_ on seizures induced by a single dose of KA (40 mg/kg i.p.), which produced severe seizures in all the mice examined in a previous study [41]. ZnCl_2_ increased the maximal seizure severity in response to KA (40 mg/kg), and both ZnCl_2_ and TC-G 1008 decreased the latency to scratching. Hence, aggravation of acute KA-induced seizures was observed in GPR39 KO [21], and TC-G 1008-treated mice. Two possible explanations exist for these findings. First, in the acute KA-seizure model (as well as in MEST, 6-Hz- or i.v. PTZ-seizure-threshold tests), we cannot exclude the involvement of other targets such as the 5-HT_1A_ receptor [29] in the action of TC-G 1008 or ZnCl_2._ Second, it is plausible that the activation of GPR39 by an endogenous agonist or constitutive activity [27] is protective against seizures, while activation by a synthetic agonist with a lower EC_50_ is detrimental. For example, the EC_50_ of the presumed endogenous agonist, zinc, is in the micromolar range (7–52 µM, depending on the signaling pathway measured) [4], while that for TC-G 1008 is below 1 nM in the Gq pathway and 60 nM in the Gs pathway [28].

The behavioral effects of TC-G 1008 on PTZ-induced epileptogenesis were linked to the GPR39 receptor. However, analysis of the downstream effects of TC-G 1008 revealed a pronounced increase in the p-CREB/CREB ratio in the hippocampus of kindled GPR39 KO mice, suggesting the non-selectivity of TC-G 1008. In a previous study, silencing of GPR39 in cells and subsequent treatment with TC-G 1008 increased the expression of CREB, compared to the siRNA-treated group, thus showing that siRNA silencing did not abolish the effects of TC-G 1008 on CREB expression [62]. This observation is in line with our data. It corroborates the non-selective nature of TC-G 1008 activity, making it imperative to verify whether the effects of TC-G 1008 in certain experimental contexts depend solely on GPR39 activation. Resolving this issue may be crucial to understanding the exact role of TC-G 1008 as a ligand of GPR39, as some of the effects may have been induced by off-target(s).

Increased activation of CREB in the hippocampus of GPR39 KO mice may be related to TC-G 1008-induced 5-HT_1A_ receptor-activity [29]. TC-G 1008 and ZnCl_2_ bind to this receptor subtype [29, 33]. The postsynaptic 5-HT_1A_ heteroreceptor is highly expressed in the hippocampus. Depending on the engagement of G protein subunits, 5-HT_1A_ activation may increase cAMP levels and subsequently increase the phosphorylation of CREB, or it may produce the reverse effect. Activation of adenylate cyclase, the biosynthetic enzyme for cAMP, was found following 5-HT_1A_ receptor activation in hippocampal cells. In addition, 5-HT_1A_ is part of the β-arrestin signaling pathway [31]. TC-G 1008 binds to 5-HT_1A_ [29] as shown by the PRESTO-Tango GPCR-ome assay, which is based on the measurement of β-arrestin.

Furthermore, 5-HT_1A_ and GPR39 receptors may oligomerize and form a trimer with the galanin type 1 receptor (GalR_1_) [77], which may explain the increased expression of CREB in KO but not WT mice. In the absence of GPR39 (GPR39 KO mice), 5-HT_1A_ monomers and 5-HT_1A_-GalR_1_ dimers abound to the exclusion of the trimer, 5-HT_1A_-GPR39-GalR_1_ and the dimer, 5-HT_1A_-GPR39. TC-G 1008, under these conditions, may act via 5-HT_1A_ to increase CREB activation. In the presence of GPR39 (WT mice), 5-HT_1A_-GPR39-GalR_1_ can exist. The characteristics of the signaling pathway involving the trimer were obtained using a GPR39 agonist (ZnCl_2_), 5-HT_1A_ agonist (8-OH-DPAT), and the serum response element (SRE) as an effector. Interestingly, the effects of ZnCl_2_ or 8-OH-DPAT on SRE were abolished [78]. The presence of the trimer in WT mice may block the effects of TC-G 1008 on other transcription factors, i.e., CREB.

Taken together, the data imply that TC-G 1008 may bind to 5-HT_1A_ or other off-target(s) and effect the phosphorylation of CREB. A limitation to this conclusion is that all the samples tested were from mice subjected to a model of epilepsy. However, the GPR39 KO mice treated with VEH did not differ from the TC-G 1008 treated GPR39 KO mice in terms of the seizure scores in this model, suggesting that TC-G 1008, and not PTZ-kindling, affected CREB activation in GPR39 KO mice. Another limitation is that we did not perform perfusion before obtaining tissues for biochemical analyses. Therefore, the results might be influenced by blood-associated mechanisms.

The effects of acute and chronic treatments with the various compounds under study and models of acute seizures or the chronic PTZ-kindling model of epilepsy on CREB activation, BDNF, and TrkB activation in the hippocampus of genetically unmodified (Swiss Albino) mice are shown in the Supplementary file. We chose the doses of compounds used in MES or 6-Hz seizure models based on the outcome of the respective seizure-threshold tests. To prevent mortality, we terminated PTZ-kindling based on the seizure score for the group with the highest seizure score. Consequently, the doses of compounds used for biochemical analysis varied between MES and the 6-Hz seizure model. The total number of PTZ injections also differed between kindling in genetically unmodified and GPR39 KO mice as well as the dose of PTZ: 40 mg/kg vs. 25 mg/kg, respectively. The difference in the doses of PTZ in Swiss Albino and C57BL/6/Tar × CBA/Tar mice was due to differences in responsiveness to this chemoconvulsant. There are strain differences in responsiveness to PTZ; therefore, a subthreshold dose that induces the kindling phenomenon has to be adjusted prior to experimentation [44]. In contrast to Swiss Albino mice that displayed stage 1 or 2 seizures after the first injection of PTZ (40 mg/kg), most C57BL/6/Tar × CBA/Tar mice exposed to PTZ (40 mg/kg) displayed stage 5 seizures after the first PTZ injection (preliminary experiment, data not shown). Therefore, lowering the PTZ dose was necessary.

Neurotrophic factors such as BDNF may be the mediators of epileptogenesis while also producing therapeutic effects due to their trophic properties [64]. p-CREB expression increased 3 min after acute seizures following i.p. administration of a single dose of PTZ (55 mg/kg) in the hippocampus and cortex [79]. Here, PTZ-induced (40 mg/kg) epileptogenesis resulted in a trend toward increased CREB and TrkB activation, measured 24 h after the last dose of PTZ (Fig. S10). At the same time TC-G 1008, ZnCl_2_, and VPA decreased the p-CREB/CREB and p-TrkB/TrkB ratios in the hippocampus of kindled mice. Hence, the changes in expression of these proteins did not correspond to the behavioral outcomes as TC-G 1008, ZnCl_2_, and VPA produced distinct effects on behavioral seizures during epileptogenesis (Fig. 5). Nevertheless, the greater number of PTZ injections and higher dose contributed to the trend toward increased CREB and TrkB activation in kindled mice, while drug treatment prevented such activation (Fig. S10). In turn, in kindled C57BL/6/Tar × CBA/Tar WT mice, the lower number of PTZ injections and a lower dose might have led to unsignificant differences in CREB and TrkB activation between animals administered VEH and TC-G 1008.

Regarding the biochemical effects of acute or chronic administration of the studied compounds in sham/non-kindled (control) animals, we found a trend toward activation of CREB and TrkB in the hippocampus after acute but not chronic treatment (Figs. S4, S7, S10). In a previous study, a single dose of TC-G 1008 in mice exposed to the forced swim test (FST) (a test used for screening for antidepressant drugs) increased BDNF protein expression 24 h after treatment [63]. In acute experiments, with BDNF protein levels analyzed 30 min after administration of TC-G 1008, we did not find significant changes in either sham mice or mice exposed to MES/6-Hz. Similarly, 30 min after administration of TC-G 1008 in mice exposed to FST there was only a trend towards increased BDNF protein expression [80]. Furthermore, in the same study, chronic administration of TC-G 1008 for 14 days did not alter the expression of BDNF 24 h after the FST [80]. Similarly, we did not observe significant changes in BDNF protein levels in either kindled or non-kindled mice after chronic administration of TC-G 1008 24 h after the last dose in the PTZ model of epileptogenesis (Fig. S10E). Taken together, the activation of the CREB-BDNF-TrkB pathway by TC-G 1008, a potential neurotrophic strategy, maybe time-dependent and transient.

We were particularly interested in the mechanisms underlying the distinct effects of TC-G 1008 and ZnCl_2_ in the MEST and 6-Hz seizure threshold test. The biochemical analyses in the Supplementary file did not allow a firm conclusion on the mechanism(s) behind the contrasting effects of GPR39 agonists in the two acute-seizure threshold tests. However, we observed that the administration of a single, low dose of TC-G 1008, which was ineffective in the MEST test in mice subjected to MES seizures, was accompanied by decreased [Zn^2+^]_I_ in the CA1 and CA3 regions of the hippocampus (Fig. S3). Moreover, administration of single doses of TC-G 1008 or ZnCl_2,_ which were effective in the 6-Hz-threshold test in mice subjected to 6-Hz seizures, was accompanied by decreased [Zn^2+^]_I_ in the CA1 and DG regions (Fig. S6). Increased [Zn^2+^]_I_ inhibited the activity of KCC2 [81]. Presumably, under decreased [Zn^2+^]_I,_ KCC2 works to maintain low intracellular chloride concentration to facilitate the inhibitory function of the GABA_A_ receptor [68]. Thus, the contrasting acute effects of TC-G 1008 in the MEST and 6-Hz seizure threshold tests might be dependent on hippocampal [Zn^2+^]_I_.

Other studies using fluorescent, cell membrane permeable probes demonstrated the association between hippocampal [Zn^2+^]_I_ and SE. Staining of hippocampal slices from pilocarpine-treated rats with TFL-Zn showed increased [Zn^2+^]_I_ staining in the CA1 region, which appeared one day after SE and declined 2–4 days afterward [57]. Moreover, staining of hippocampal sections with TFL-Zn revealed that zinc accumulated in CA1 and CA3 neurons after KA-induced seizures [41]. Furthermore, an association between increased [Zn^2+^]_I_ and its most putative sensor, MTF1 [60], was depicted in an animal model of SE and human hippocampi surgically resected from patients with pharmacoresistant epilepsy [57]. We found a trend towards increased MTF1 in the hippocampus of WT (C57BL/6/Tar × CBA/Tar) mice subjected to PTZ-kindling that experienced enhanced epileptogenesis, which was accompanied by a small but significant increase in [Zn^2+^]_I_. However, we examined [Zn^2+^]_I_ and MTF1 once (24 h after the last injection of PTZ). A study by van Loo et al. showed increased expression of MTF1 6 and 12 h after pilocarpine-induced SE, which returned to basal values thereafter. The lack of robust differences in either [Zn^2+^]_I_ or MTF1 may be due to sampling time or a trait of the selected epilepsy model. As the differences in [Zn^2+^]_I_ were not robust, the lack of significant differences in [Zn^2+^]_I_ in PTZ-kindled Swiss Albino mice that received TC-G 1008 (Fig. S9) may be explained by the dose of PTZ and the number of injections and/or smaller number of analyzed brains compared to those used in C57BL/6/Tar × CBA/Tar mice.

Serum zinc level has been suggested as epilepsy biomarker [58]. GPR39 KO mice subjected to PTZ-kindling had decreased total serum zinc concentration compared to WT mice. However, the changes in serum of GPR39 KO mice were not accompanied by changes in total zinc in the hippocampus, as shown by semi-quantitative LA-ICP-MS, possibly because of homeostatic zinc control mechanisms in the brain. Chronic treatment with TC-G 1008 did not affect total serum zinc in either KO or WT (C57BL/6/Tar × CBA/Tar) mice or Swiss Albino mice (Figs. S2, S5, S8). Equally, a previous study showed no changes in total serum zinc concentration after a 2-week administration of TC-G 1008 [80]. However, the changes in [Zn^2+^]_I_ which is more easily exchangeable than total zinc were found and are discussed above.

**Fig. 10:**
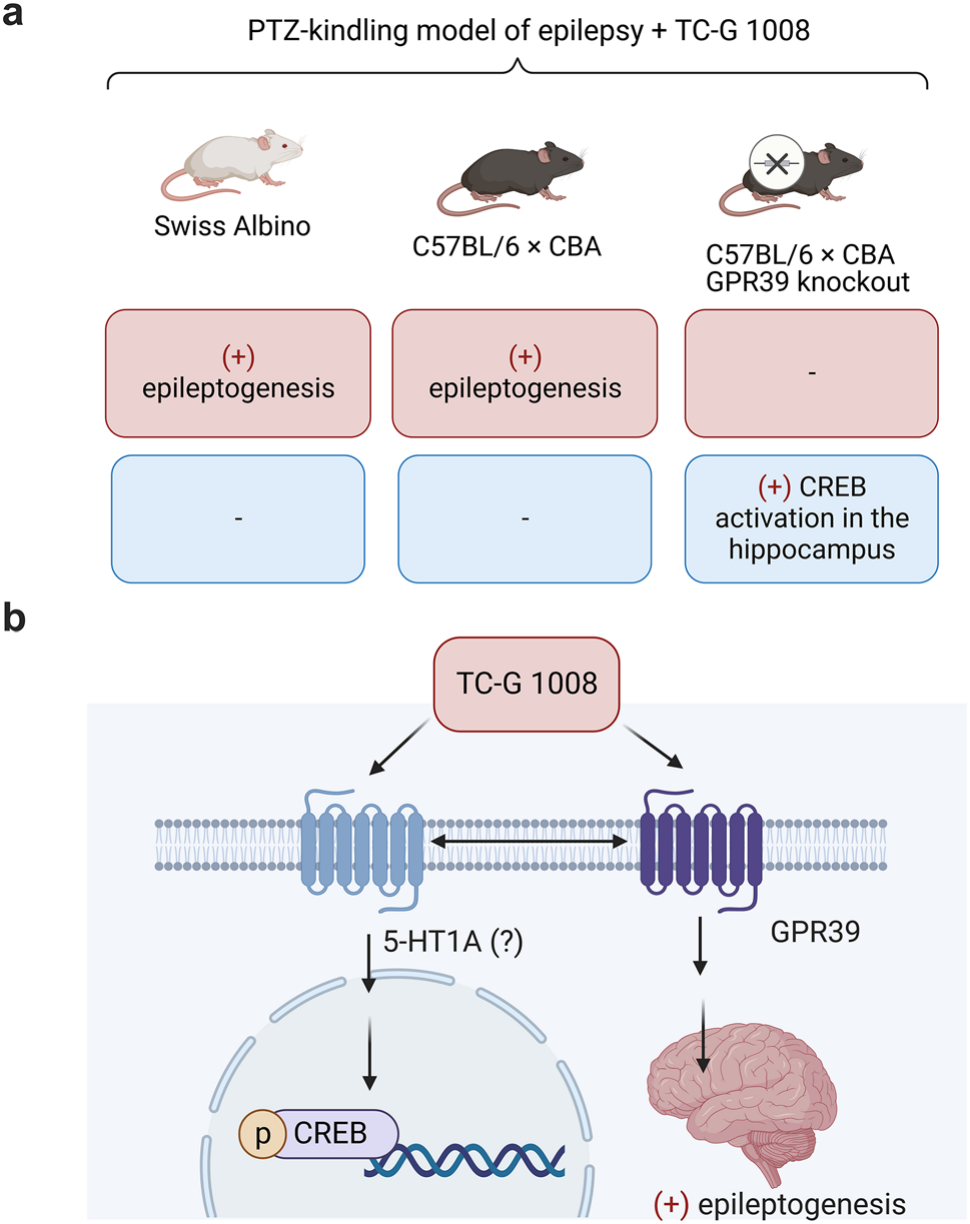
Summary of findings and the proposed mechanism. TC-G 1008 is a small molecule agonist at GPR39 [28]. GPR39 interacts physically with the serotonin 5-HT_1A_ receptor [78]. TC-G 1008 also binds to the 5-HT_1A_ receptor [29]. **a** We found that TC-G 1008 facilitated PTZ-epileptogenesis in vivo by acting selectively at GPR39*.* However, it increased the activation of CREB in the hippocampus of kindled GPR39 KO mice. **b** Our data showed that TC-G 1008 acts at GPR39 and other targets, presumably the 5-HT_1A_ receptor. (+) enhancement

In summary, we showed that the behavioral effects of TC-G 1008 on epileptogenesis induced by PTZ are mediated selectively by the GPR39 receptor. However, we also demonstrated that the effects on CREB activation in the hippocampus are induced by other targets, thereby showing for the first time the non-selective activity of TC-G 1008 in animal brain tissue. Given the in vitro data on this small molecule agonist [29], we postulate that the serotonin 5-HT_1A_ receptor may account for the marked increase in CREB activation (Fig. 10). Increased CREB activation in KO mice may have been also induced by TC-G 1008 acting at other, so far unknown molecular targets. Therefore, we suggest a critical reassessment of the existing literature, as some of the effects previously attributed to TC-G 1008 action at GPR39 may have arisen from different mechanisms. The use of other GPR39 agonists may be necessary if any progress is to be made in validating GPR39 as a drug target. Nonetheless, our data consistently showed that activation of the GPR39 receptor aggravated in vivo epileptogenesis, thus arguing against the hypothesis that GPR39 activation is a potential therapeutic strategy for treating epilepsy. The worsening of neurological diseases/epilepsies may be a side-effect of new drugs that are GPR39 agonists.

## Supplementary Information

Below is the link to the electronic supplementary material.

Supplementary file1 (DOCX 31449 KB)

## Data Availability

The data that support the findings of this study are available from the corresponding author upon reasonable request.
