# Supplementary material for: TC-G 1008 facilitates epileptogenesis by acting selectively at the GPR39 receptor but non-selectively activates CREB in the hippocampus of pentylenetetrazole-kindled mice
Source: Cell Mol Life Sci. 2023 Apr 25;80(5):133. doi: 10.1007/s00018-023-04766-z (PMC10130118; doi:10.1007/s00018-023-04766-z)
Supplement: Supplementary file 1 — Supplementary file1 (DOCX 31449 KB) [file 18_2023_4766_MOESM1_ESM.docx]

*Supplementary file*

**TC-G 1008 facilitates epileptogenesis by acting selectively at the GPR39 receptor but non-selectively activates CREB in the hippocampus of pentylenetetrazole-kindled mice**

Urszula Doboszewska^1*^, Katarzyna Socała^1^, Mateusz Pieróg^1^, Dorota Nieoczym^1^, Jan Sawicki^2^, Małgorzata Szafarz^3^, Kinga Gaweł^4^, Anna Rafało-Ulińska^5^, Adam Sajnóg^6^, Elżbieta Wyska^3^, Camila V. Esguerra^7^, Bernadeta Szewczyk^5^, Marzena Maćkowiak^8^, Danuta Barałkiewicz^6^, Katarzyna Młyniec^9^, Gabriel Nowak^9^, Ireneusz Sowa^2^, Piotr Wlaź^1^

^1^Department of Animal Physiology and Pharmacology, Institute of Biological Sciences, Maria Curie-Skłodowska University, Akademicka 19, PL 20-033 Lublin, Poland

^2^Department of Analytical Chemistry, Medical University of Lublin, Chodzki 4A, PL 20-093 Lublin, Poland

^3^Department of Pharmacokinetics and Physical Pharmacy, Jagiellonian University Medical College, Medyczna 9, PL 30-688 Kraków, Poland

^4^Department of Experimental and Clinical Pharmacology, Medical University of Lublin, Jaczewskiego 8b, 20-090 Lublin, Poland

^5^Department of Neurobiology, Maj Institute of Pharmacology, Polish Academy of Sciences, Smetna 12, PL 31-343 Krakow, Poland

^6^Department of Trace Analysis, Adam Mickiewicz University, Uniwersytetu Poznanskiego 8,
61-614 Poznan, Poland

^7^Chemical Neuroscience Group, Centre for Molecular Medicine Norway, University of Oslo, Gaustadalléen 21, Forskningsparken, 0349 Oslo, Norway

^8^Laboratory of Pharmacology and Brain Biostructure, Department of Pharmacology, Maj Institute of Pharmacology, Polish Academy of Sciences, Smetna 12, PL 31-343 Krakow, Poland

^9^Department of Pharmacobiology, Jagiellonian University Medical College, Medyczna 9, PL 30-688 Kraków, Poland

**The file contains:**

- **Supplementary Tables (S1- S5)**
- **Supplementary Figures (S1 – S12)**
- **Statistical details to Supplementary Figures (S1 – S10)**

**Table S1.** ***Pharmacokinetic parameters of TC-G 1008 in mice***

| Parameter | Serum | Brain |
| --- | --- | --- |
| t_max_ (min) | 15 | 15 |
| C_max_ (ng/mL) | 2930 | 36.32 |
| λ_z_ (min^-1^) | 0.015 | 0.014 |
| t_0.5 λz_ (min) | 47.77 | 50.18 |
| V_z_/F (L/kg) | 6.47 | - |
| CL/F (L/min/kg) | 0.97 | - |
| AUC_0-t_ (ng⋅min/mL) | 172860 | 2309 |
| AUC_0-∞_ (ng⋅min/mL) | 213045.4 | 2911.6 |
| MRT (min) | 73.18 | 77.50 |

Pharmacokinetic parameters of TC-G 1008 in mice were estimated using the non-compartmental analysis following administration of a single dose of TC-G 1008 (20 mg/kg, i.p.).

C_max_ – maximum serum concentration, t_max_ – time to reach maximum concentration, λ_z_ – terminal slope, t_0.5λz_ – terminal half-life, AUC_0-t_ – area under the concentration-time curve from the time of dosing to the last measured point, AUC_0-∞_ – area under the concentration-time curve from the time of dosing to infinity, V_z_ – volume of distribution based on the terminal phase, CL – clearance, F – fraction absorbed, MRT – mean residence time.

**Table S2. *The effects of* i.p. *administration of single doses of VPA, TC-G 1008 or ZnCl_2_ on motor coordination and neuromuscular strength***

| Drug (mg/kg) | | Impairment of motor performance (%) | Neuromuscular strength  (mN/g) |
| --- | --- | --- | --- |
|  | VEH | 0 | 25.52 ± 1,05  1,05 |
|  | VPA 50 | 0 | 28.11 ± 1,112 |
|  | VPA 150 | 0 | 28.14 ± 1,02 |
|  | TC-G 1008 2.5 | 0 | 27.4 ± 0,7912 |
|  | TC-G 1008 5 | 8 | 25.89 ± 1,255 |
|  | TC-G 1008 10 | 0 | 29.12 ± 0,709* |
|  | TC-G 1008 20 | 0 | 29.77 ± 0,5941** |
|  | TC-G 1008 40 | 8 | 29.08 ± 0,9143* |
|  | Zn 1 | 0 | 28.45 ± 1,946 |
|  | Zn 2 | 0 | 27.45 ± 1,103 |
|  | Zn 4 | 0 | 27.56 ± 0,751 |
|  | Zn 8 | 8 | 27.15 ± 1,105 |
|  | Zn 16 | 17 | 31.24 ± 1,087*** |

Swiss Albino mice received VPA, TC-G 1008, or ZnCl_2_ and were subjected to the grip-strength and chimney tests. Control animals received vehicle VEH. Data from the grip-strength test are expressed as means ± SEM of the neuromuscular strength; data from the chimney test are expressed as % of animals that displayed motor coordination impairment. n=12 in each group, outlier excluded in TC-G 1008 2.5 group. F(2,31)=2.03, p=0.1485**,** One-way ANOVA (VPA); F(5,65)=3.924, p=0.0036, One-way ANOVA; p=0.5331 Chi-square test (TC-G 1008); F(5,66)=2.381, p=0.0478, One-way ANOVA; p=0.1990 Chi-square test (Zn). *P<0.05, **p<0.01, *** p<0.001 vs. VEH

**Table S3. *The effects of chronic treatment with VPA, TC-G 1008, or ZnCl_2,_ and PTZ-induced kindling model of epilepsy on neuromuscular strength and motor coordination***

| Drug (mg/kg) | | Impairment  of motor performance (%) | Neuromuscular strength  (mN/g) |
| --- | --- | --- | --- |
|  | Non-kindled VEH | 0 | 30.6 ± 1.14 |
|  | Non-kindled VPA 150 | 0 | 33.02 ± 0.89 |
|  | Non-kindled TC-G 1008 10 | 0 | 30.4 ± 1.33 |
|  | Non-kindled Zn 8 | 17 | 30.81 ± 1.61 |
|  | Kindled VEH | 0 | 28.65 ± 1.35 |
|  | Kindled VPA 150 | 0 | 29.21 ± 0.78 |
|  | Kindled TC-G 1008 10 | 0 | 28.61 ± 1.10 |
|  | Kindled Zn 8 | 0 | 30.55 ± 1.38 |

Swiss Albino mice received VPA (150 mg/kg), TC-G 1008 (10 mg/kg), ZnCl_2_ (8 mg Zn/kg), or VEH during PTZ-kindling. The kindling model consisted of 19 injections of PTZ (40 mg/kg). The non-kindled mice received the respective doses of drugs or VEH but received 0.9% NaCl instead of PTZ. Neuromuscular strength and motor coordination were assessed in kindled and non-kindled mice on the last day of PTZ-kindling. n=12 in each group. Interaction [F(3,88)=0.3337, p=0.801], drug [F(3,88)=0.3337, p=0.1042], PTZ [F(1,88)=2.37, p=0.1272], Two-way ANOVA; p>0.05 the Fisher’s exact test.

**Table S4**. ***The effects of GPR39 gene knockout (KO) on neuromuscular strength and on motor coordination***

| Genotype | | Impairment  of motor performance (%) | Neuromuscular strength  (mN/g) |
| --- | --- | --- | --- |
|  | WT | 15 | 42.75 ± 1.35 |
|  | GPR39 KO | 0 | 39.41 ± 1.73 |

Experimentally naïve GPR39 KO and wild-type (WT) (C57BL/6/Tar × CBA/Tar) mice were subjected to the grip strength and chimney tests. n=20 in each group. t(38)=1.521, p=0.1365, Student’s t-test; p=0.2308 the Fisher’s exact test.

**Table S5. *The effects of chronic treatment with TC-G 1008 and PTZ-induced kindling model of epilepsy in GPR39 KO or WT mice on neuromuscular strength and motor coordination***

| Genotype /treatment (mg/kg) | | Impairment  of motor performance (%) | Neuromuscular strength  (mN/g) |
| --- | --- | --- | --- |
|  | WT VEH kindled | 0 | 41.2 ± 1.16 |
|  | WT TC-G 1008 10 kindled | 0 | 37.87 ± 1.32 |
|  | GPR39 KO VEH kindled | 0 | 37.52 ± 1.36 |
|  | GPR39 KO TC-G 1008 10 kindled | 0 | 45.35 ± 1.85**^,##^ |

GPR39 KO or WT (C57BL/6/Tar × CBA/Tar) mice received TC-G 1008 (10 mg/kg) or VEH during PTZ-kindling. The kindling model consisted of 14 injections of PTZ (25 mg/kg). Neuromuscular strength and motor coordination were assessed on the last day of PTZ-kindling. n**=**8 WT VEH, n=7 WT TC-G 1008, n=8 KO VEH, n=7 KO TC-G 1008. Genotype x treatment [F(1,26)=15.3, p=0.0006], genotype [F(1,24)=1.773, p=0.1946], treatment [F(1,24)=2.474, p=0.1278], Two-way AVOVA. **p<0.01 vs. GPR39 KO VEH kindled, ^##^p<0.01 vs. WT TC-G 1008 10 kindled

**Fig S1**


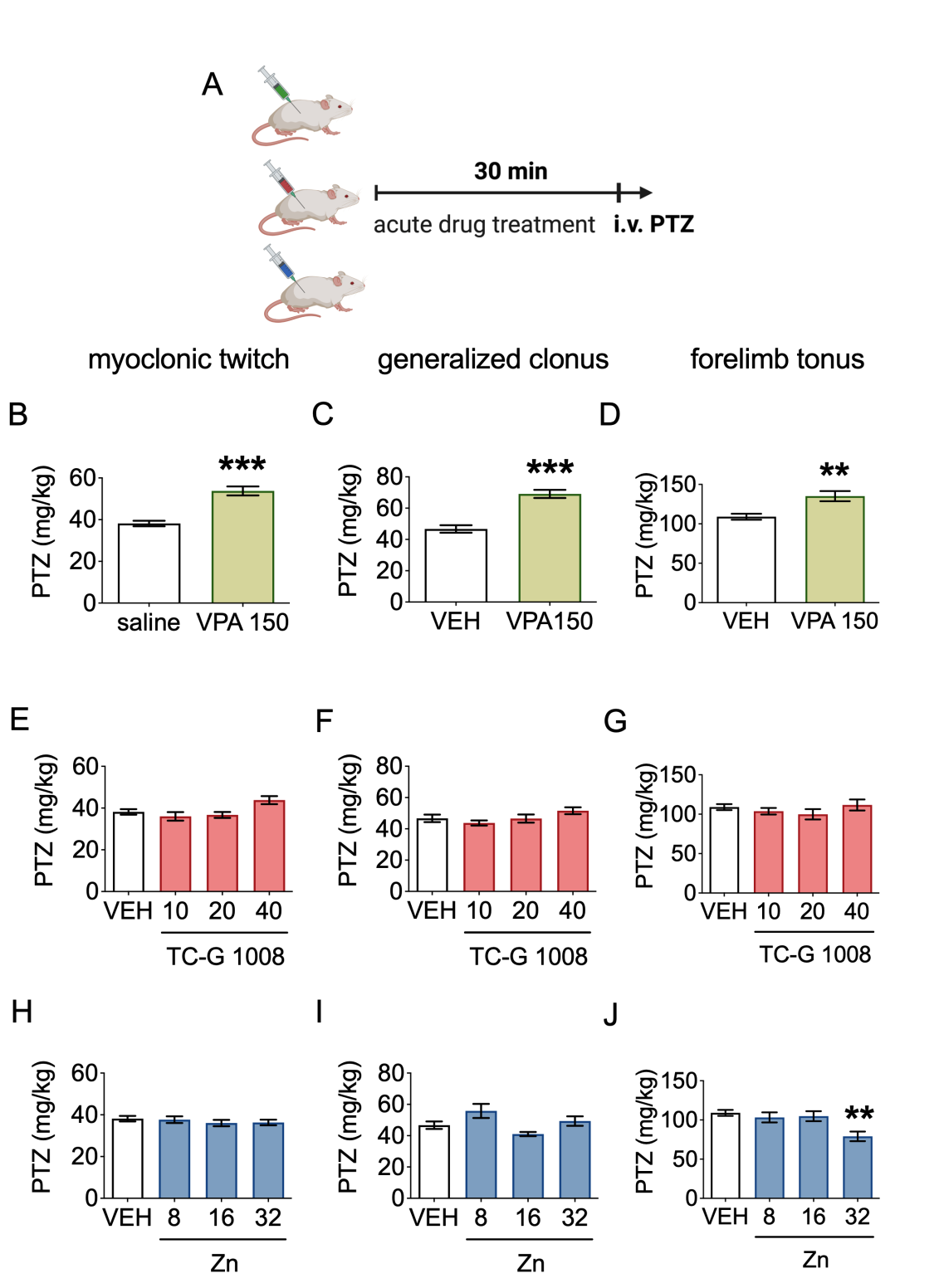


**Fig S1. *The effects of single doses of VPA, TC-G 1008, or ZnCl_2_ on the seizure threshold induced by* i.v. *infusion of pentylenetetrazole (PTZ).* (A)** Experimental paradigm*.* Drugs were administered i.p., 30 min before the i.v. infusion of PTZ. **(B-J)** The doses of drugs are shown on abscissas in mg/kg. Data are presented as the dose of PTZ (in mg/kg) ± SEM needed to produce the first apparent sign of each endpoint. P values were determined by the Student’s t-test or one-way ANOVA and Dunnett’s multiple comparison test. ***P<0.001, **p<0.01.

VPA (150 mg/kg) significantly increased the seizure threshold for myoclonic twitch (**Fig S1 B**), generalized clonus (**Fig S1 C**), and forelimb tonus (**S1 D**) in the i.v. PTZ-seizure threshold test. TC-G 1008 (10, 20 and 40 mg/kg) (**Fig S1 E**) or ZnCl_2_ (8, 16, and 32 mg Zn/kg) (**Fig S1 H**) did not significantly affect the threshold for myoclonic twitch. Also, these compounds did not significantly affect generalized clonus (**Fig S1 F, S1 I**, respectively). TC-G 1008 (10, 20, and 40 mg/kg) (**Fig S1 G**) and ZnCl_2_ (8 and 16 mg Zn/kg) did not significantly affect the threshold for forelimb tonus, while ZnCl_2_ at a high dose of 32 mg Zn/kg significantly decreased the threshold for this endpoint (**Fig S1 J**).

**Fig S2**

**
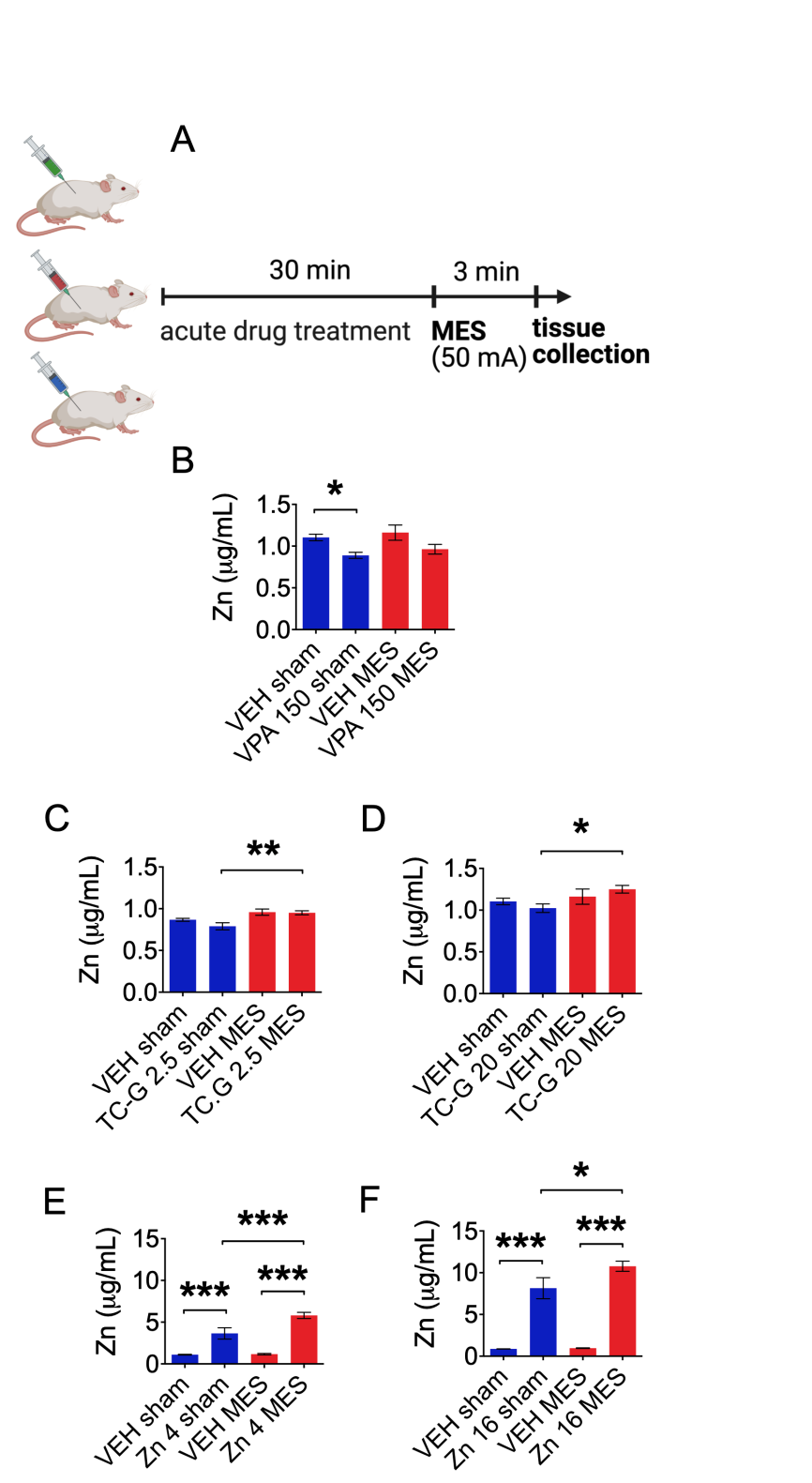
**

**Fig S2. *The effects of single doses of VPA, TC-G 1008, or ZnCl_2_ and acute maximal electroshock seizures (MES) on total serum zinc concentration.*** **(A)** Experimental paradigm*.* Drugs: VPA (150 mg/kg), TC-G 1008 (2.5 or 20 mg/kg), ZnCl_2_ (4 or 16 mg Zn/kg), or VEH were administered *i.p*., 30 min before MES. The seizures were generated by a supramaximal current intensity of 50 mA. Non-stimulated (sham) mice received the respective doses of drugs or VEH but did not receive electrical stimulus. Tissues for biochemical analyses were obtained ca. 3 min after MES. **(B-F)** Total serum zinc concentration was measured by Inductively Coupled Plasma Optical Emission Spectrometry (ICP-OES). Data are expressed as means ± SEM. P values were determined by two-way ANOVA and Bonferroni’s multiple comparison test. *P<0.05, **p<0.01, ***p<0.001.

MES did not significantly affect total serum zinc concentration. Serum zinc concentration markedly and significantly increased after the administration of single doses of ZnCl_2_ of 4 mg Zn/kg (**Fig S2 E**) or 16 mg Zn/kg (**Fig S2 F**) in sham mice and mice that received MES. On the other hand, serum zinc concentration decreased after the administration of a single dose of VPA (150 mg/kg) in sham mice (**Fig S2 B**). However, it increased in mice that received TC-G 1008 at a dose of 2.5 (**Fig S2 C**) or 20 (**Fig S2 D**) mg/kg or ZnCl_2_ at a dose of 4 mg Zn/kg (**Fig S2 E**) or 16 mg Zn/kg (**Fig S2 F**) and MES_,_ compared to sham mice that received the respective doses of drugs.

**Fig S3.**

**~~
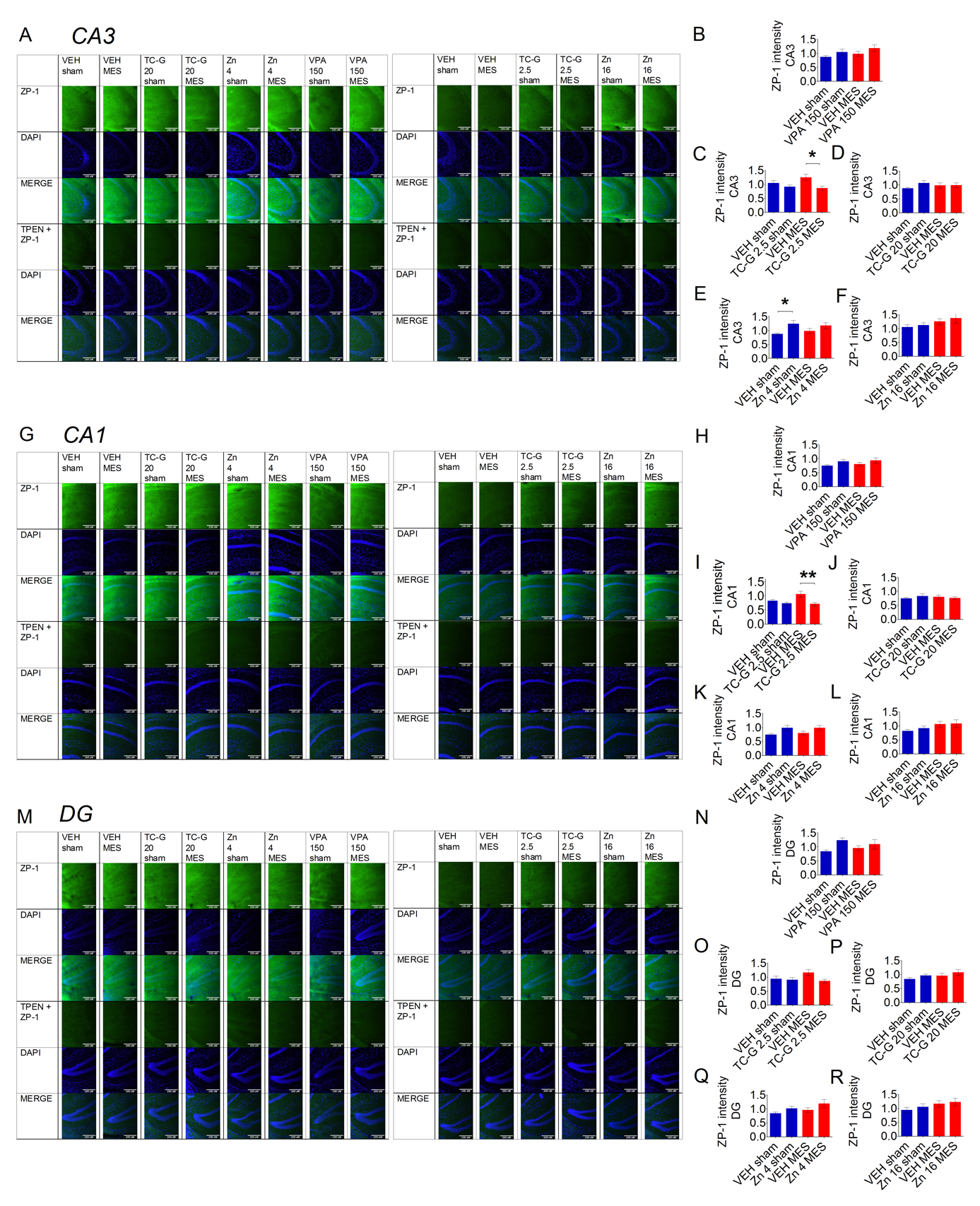
~~**

**Fig S3. *The effects of single doses of VPA, TC-G 1008, or ZnCl_2_ and acute MES seizures on intracellular Zn^2+^ ([Zn^2+^]_I_) in the CA3, CA1, and dentate gyrus (DG) regions of the hippocampus.*** Hippocampal sections were analyzed ca. 3 min after MES. Sections were double stained with Zinpyr-1 (ZP-1) for fluorescent detection of [Zn^2+^]_I_ and with DAPI to show cell nuclei. (**A, G, M**) Magnifications of ZP-1 (green), DAPI (blue), and TPEN / Zinpyr-1 in the CA3, CA1, and DG regions are shown. Merged images include ZP-1 (green), TPEN / ZP-1, and DAPI (blue). ZP-1 fluorescence is primarily absent in the TPEN-treated section, thus revealing the high specificity of Zn2+-staining in brain sections. Scale bar=200 μm. (**B-F, H-L, N-R**) The ratio of mean ZP-1 grey values between mouse sections belonging to treatment groups: VPA (150 mg/kg), TC-G 1008 (2.5 or 20 mg/kg), ZnCl_2_ (4 or 16 mg Zn/kg) is shown. Data are expressed as means ± SEM. P values were determined by two-way ANOVA and Bonferroni’s multiple comparison test. *P<0.05, **p<0.01.

***A non-effective dose of TC-G 1008 in the MEST test decreased [Zn^2+^]_I_ in the hippocampus***

MES did not significantly affect [Zn^2+^]_I_ in the three analyzed regions of the hippocampus. [Zn^2+^]_I_ was decreased in the CA3 (**Fig S3 C**) and CA1 (**Fig S3 I**) regions in mice that received a single dose of TC-G 1008 (2.5 mg/kg) and MES, compared to mice that received VEH and MES. There was a trend towards decreased [Zn^2+^]_I_ in the DG of those mice (**Fig S3 O**). The 2.5 mg/kg dose of TC-G 1008 was ineffective in the MEST test (**Fig 3D**). There were no significant differences in [Zn^2+^]_I_ in the CA3 (**Fig S3 D**), CA1 (**Fig S3 J**), or DG (**Fig S3 P**) regions between mice that received 20 mg/kg, TC-G 1008 compared to mice that received VEH and MES. TC-G 1008 (20 mg/kg) decreased the threshold for seizures in the MEST test (**Fig 3D**). In addition, a single dose of ZnCl_2_ (4 mg Zn//kg) increased [Zn^2+^]_I_ in the CA3 region in sham mice (**Fig S3 E**).

**Fig S4**


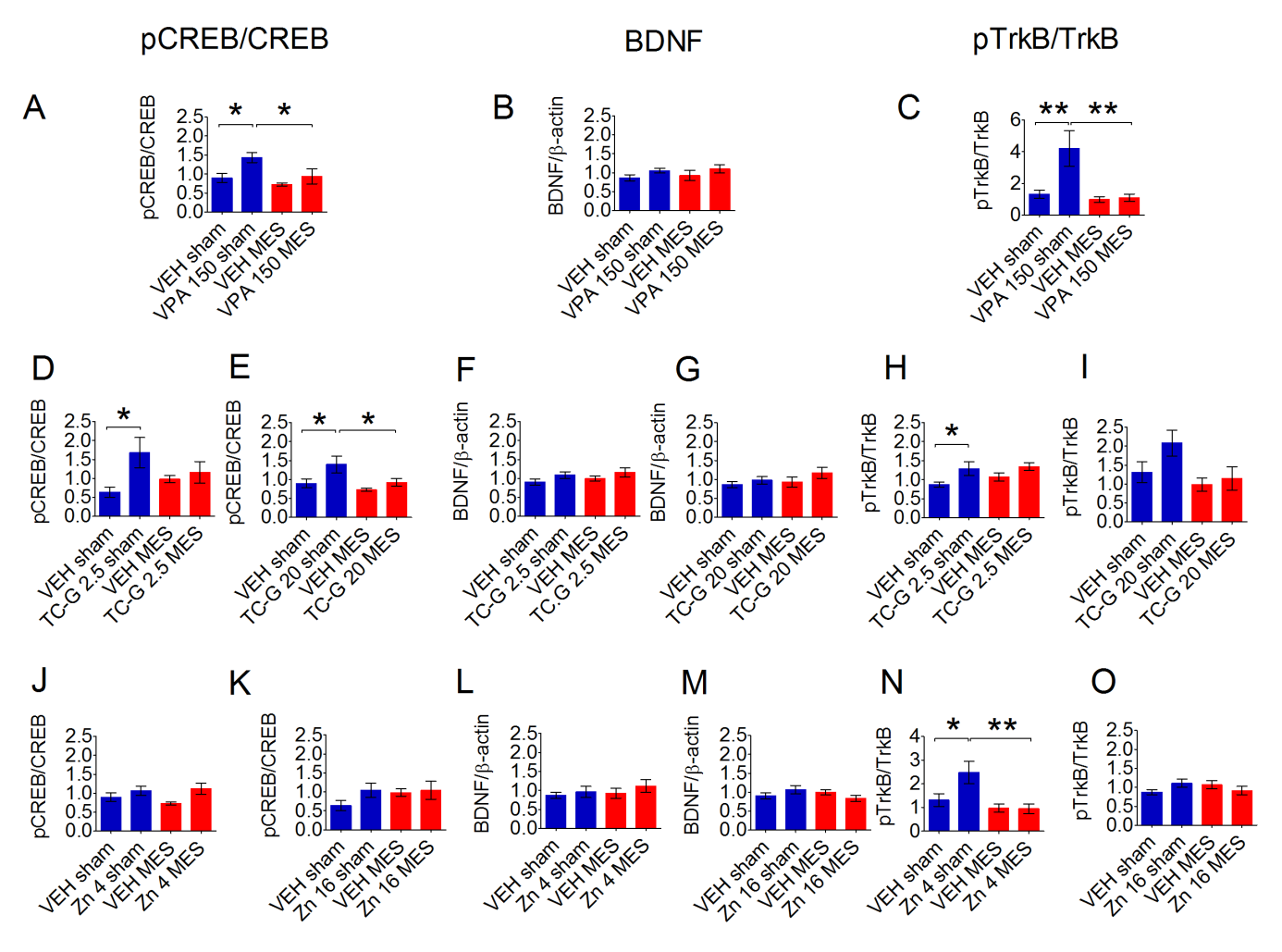


**
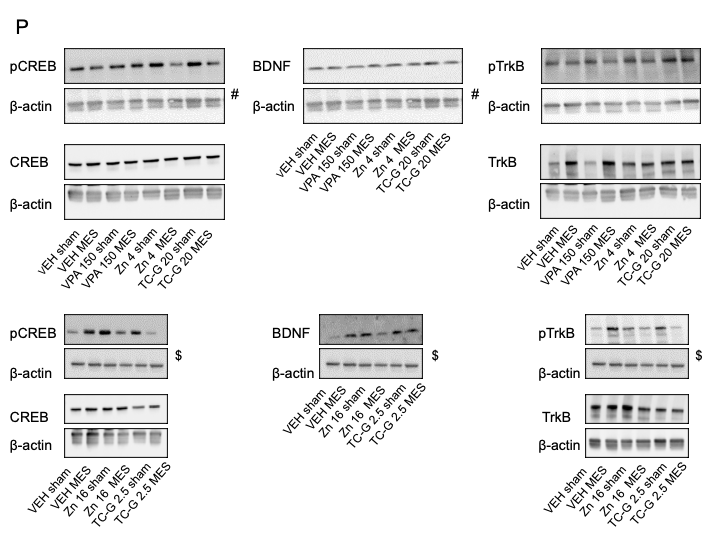
**

**Fig S4**. ***The effects of single doses of VPA, TC-G 1008, or ZnCl_2_ and acute MES seizures on the expression of various proteins (phosphorylated CREB at Ser 133 (p-CREB), total CREB, BDNF, phosphorylated TrkB at Tyr 816 (p-TrkB) and TrkB) in the hippocampus.*** Hippocampal samples were analyzed ca. 3 min after MES. (**A-O**) The results (mean ± SEM) are shown as p-CREB/CREB, BDNF/β-actin, or p-TrkB/TrkB ratios. P values were determined by two-way ANOVA and Bonferroni’s multiple comparison test. *P<0.05, **p<0.01. (**P**) Representative blots of p-CREB, CREB (~46 kDa), BDNF (~14 kDa), p-TrkB, TrkB (~140 kDa), and β-actin (~42 kDa) in the hippocampi of mice. ^#^p-CREB and BDNF are from the same blot; ^$^p-CREB, BDNF and p-TrkB are from the same blot, thus sharing corresponding β-actin bands.

MES did not significantly affect the activation of CREB or TrkB in the hippocampus, measured as p-CREB/CREB or p-TrkB/TrkB ratios, respectively.

A single dose of VPA (150 mg/kg) increased p-CREB/CREB (**Fig S4 A**) and p-TrkB/TrkB (**Fig S4 C**) ratios in the hippocampus of sham mice. Moreover, mice that received VPA and MES displayed decreased p-CREB/CREB (**Fig S4 A**) and p-TrkB/TrkB (**Fig S4 C**) ratios compared to sham mice that received the respective compounds.

Also, single doses of 2.5 mg/kg TC-G 1008 (**Fig S4 D**) and 20 mg/kg (**Fig S4 E**) increased the p-CREB/CREB ratio in sham mice. In addition, 2.5 mg/kg TC-G 1008 increased the p-TrkB/TrkB ratio in sham mice (**Fig S4 H**). Furthermore, there was a trend towards increased p-TrkB/TrkB after administration of 20 mg/kg TC-G 1008 in sham mice (**Fig S4 I**). Mice that received 2.5 mg/kg TC-G 1008 and MES had decreased p-CREB/CREB in the hippocampus compared to sham mice (**Fig S4 D**). Mice that received 20 mg/kg TC-G 1008 and MES displayed a trend towards decreased p-CREB/CREB in the hippocampus compared to sham mice (**Fig S4 E**).

Furthermore, a single dose of ZnCl_2_ (4 mg Zn/kg) increased the p-TrkB/TrkB ratio in sham mice. The p-TrkB/TrkB ratio was lower in mice that received 4 mg Zn/kg and MES compared to sham mice (**Fig S4 N**). Neither the acutely administered compounds nor MES significantly affected the BDNF protein level.

**Fig S5**

**
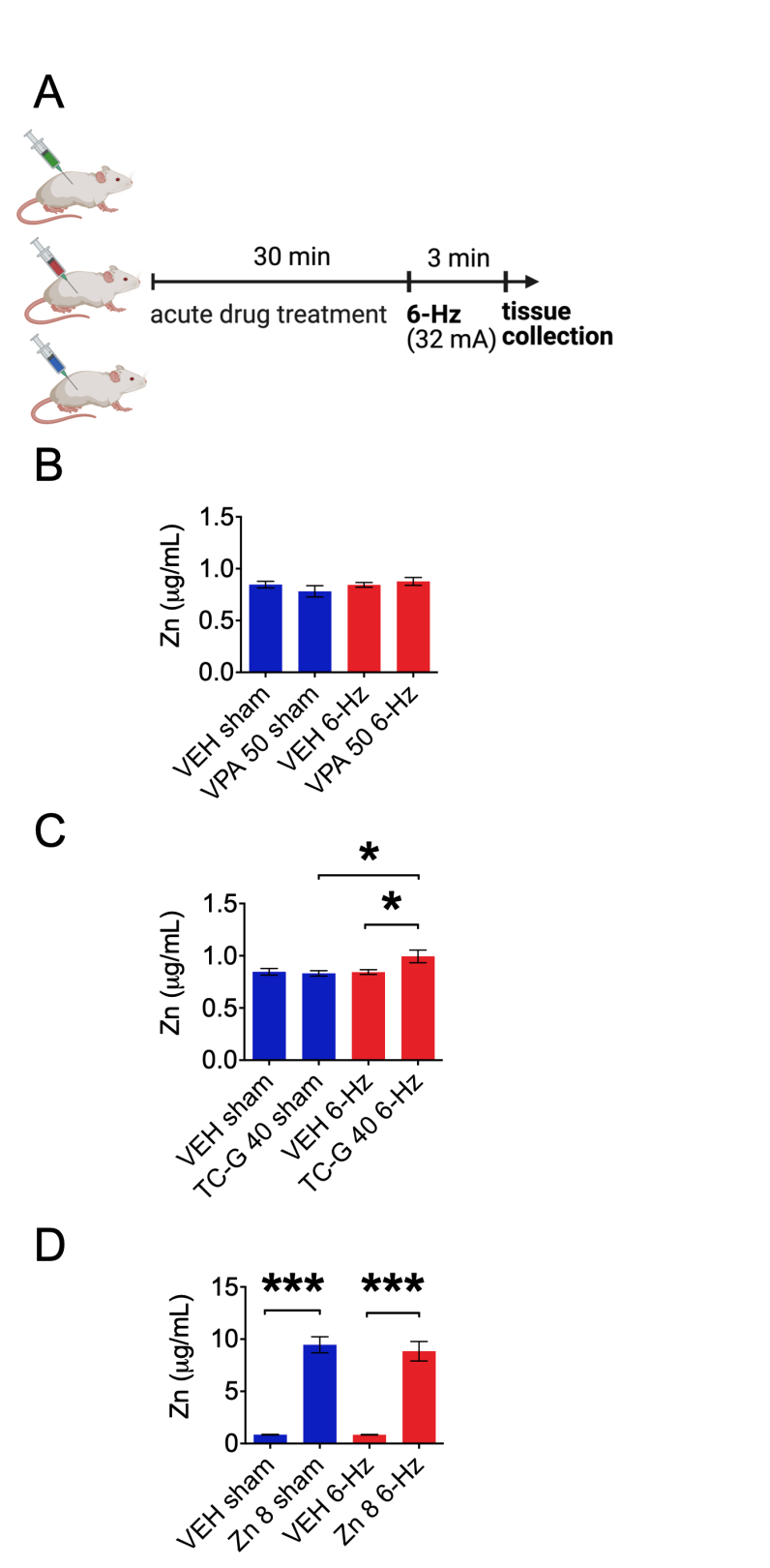
**

**Fig S5. *The effects of single doses of VPA, TC-G 1008, or ZnCl_2_ and acute 6-Hz seizures*** ***on total serum zinc concentration****.* **(A)** Experimental paradigm. Drugs: VPA (50 mg/kg), TC-G 1008 (40 mg/kg), ZnCl_2_ (8 mg/kg), or VEH were administered *i.p*. 30 min before 6-Hz seizures. The seizures were generated by a supramaximal current intensity of 32 mA. Non-stimulated (sham) mice received the respective doses of drugs or VEH but not electrical stimulus. Tissues for biochemical analyses were obtained ca. 3 min after 6-Hz seizures. **(B-D)** Total serum zinc concentration was measured by ICP-OES. Data are expressed as means ± SEM. P values were determined by two-way ANOVA and Bonferroni’s multiple comparison test. *P<0.05, ***p<0.001.

6-Hz seizures did not significantly affect total serum zinc concentration. Serum zinc concentration markedly and significantly increased after administration of a single dose of ZnCl_2_ (8 mg Zn/kg) in both sham mice and mice that received 6-Hz seizures (**Fig S5 D**). Moreover, serum zinc concentration increased in mice that received TC-G 1008 (40 mg/kg) and 6-Hz seizures, compared to sham mice that received this drug (**Fig S5 C**). Serum zinc concentration also increased in mice that received TC-G 1008 (40 mg/kg) and 6-Hz seizures, compared to mice that received VEH and electrical stimulus (**Fig S5 C**). This effect was not observed in either sham mice or mice that received 6-Hz seizures and VPA (**Fig S5 B**).

**Fig S6**

**
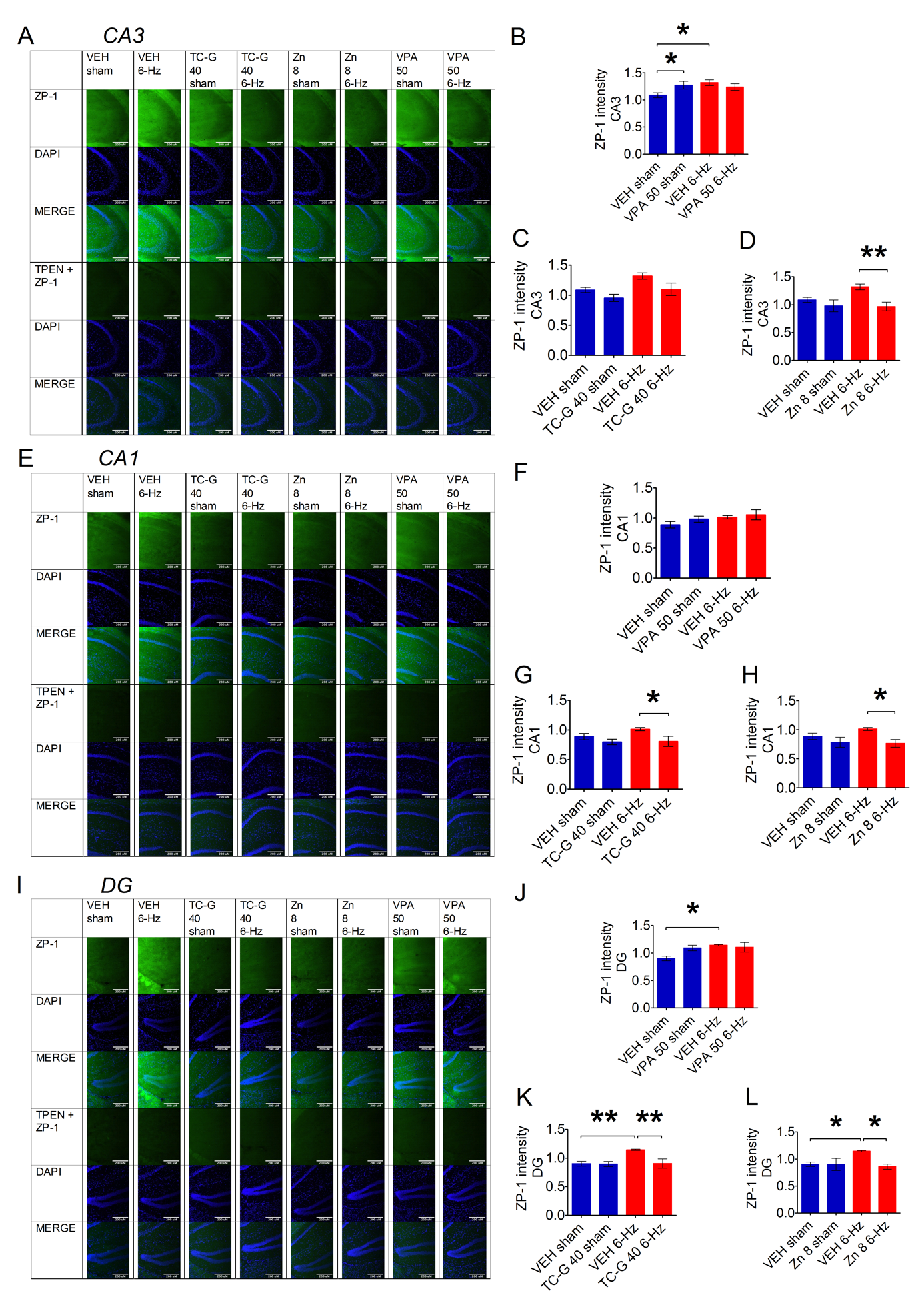
**

***Fig S6. The effects of single doses of VPA, TC-G 1008, or ZnCl_2_ and acute 6-Hz seizures*** ***on [Zn^2+^]_I_ in the* *CA3, CA1 and DG regions of the hippocampus.*** Hippocampal sections were analyzed ca. 3 min after 6-Hz seizures. (**A, E, I**) Magnifications of ZP-1 (green), DAPI (blue), and TPEN / ZP-1 in the CA3, CA1, and DG regions are shown. Merged images include ZP-1 (green), TPEN / ZP-1, and DAPI (blue). ZP-1 fluorescence is primarily absent in the TPEN-treated section. Scale bar=200 μm. (**B-D, F-H, J-L**) The ratio of mean ZP-1 grey values between mouse sections belonging to treatment groups: VPA (50 mg/kg), TC-G 1008 (40 mg/kg), and ZnCl_2_ (8 mg/kg) are shown. Data are expressed as means ± SEM. P values were determined by two-way ANOVA and Bonferroni’s multiple comparison test. *P<0.05, **p<0.01.

***The TC-G 1008 dose that increased the seizure threshold in the 6-Hz seizure threshold test also decreases [Zn^2+^]_I_ in the hippocampus***

6-Hz seizures increased [Zn^2+^]_I_ in the DG region of the hippocampus (**Fig S6 J-L**). There was also a trend towards increased [Zn^2+^]_I_ in the CA3 region after 6-Hz seizures (**Fig S6 B-D**). A single dose of VPA (50 mg/kg) increased [Zn^2+^]_I_ in the CA3 region of the hippocampus of sham mice (**Fig S6 B**). Administration of TC-G 1008 (40 mg/kg) in mice subjected to 6-Hz seizures decreased [Zn^2+^]_I_ in the CA1 (**Fig S6 G**) and DG (**Fig S6 K**) regions, compared to mice that received VEH and electrical stimulus. Similarly, administration of ZnCl_2_ (8 mg Zn/kg) and 6-Hz seizures decreased [Zn^2+^]_I_ in the CA3 (**Fig S6 D**), CA1 (**Fig S6 H**), and DG (**Fig S6 L**) regions of the hippocampus, compared to mice that received VEH and electrical stimulus. TC-G 1008 (40 mg/kg) and ZnCl_2_ (8 mg Zn/kg) increased the seizure threshold in the 6-Hz seizure threshold test, respectively (**Fig 3E, G** ).

**Fig S7**


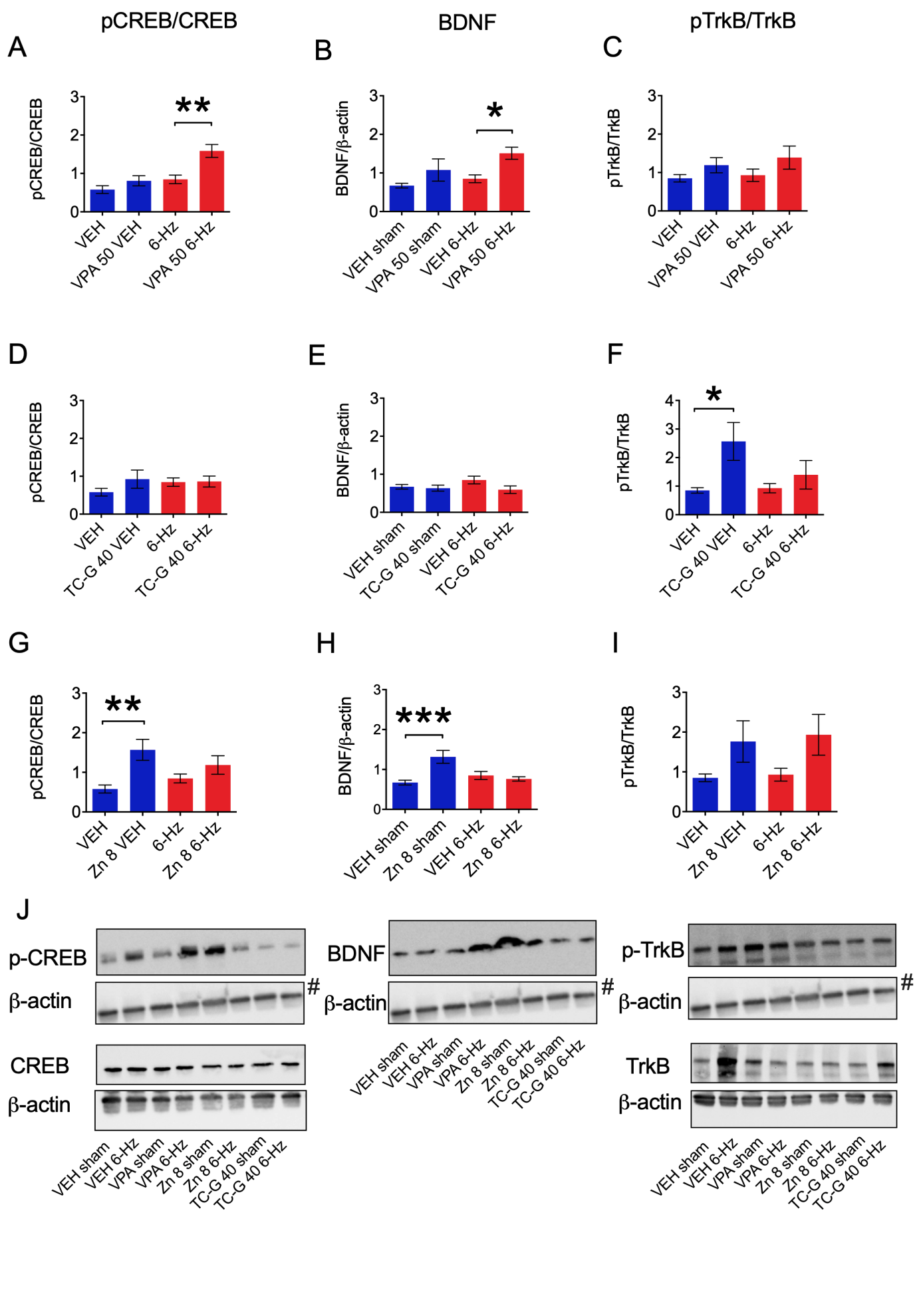


**Fig S7. *The effects of single doses of VPA, TC-G 1008, or ZnCl_2_ and acute 6-Hz seizures on the expression of various proteins (phosphorylated CREB at Ser 133 (p-CREB), total CREB, BDNF, phosphorylated TrkB at Tyr 816 (p-TrkB) and TrkB) in the hippocampus.*** Hippocampal samples were analyzed at ca. 3 min after 6-Hz seizures, which were induced 30 min after drug administration. (**A-I**) The results (mean ± SEM) are shown as the p-CREB/CREB or BDNF/β-actin or p-TrkB/TrkB ratios. P values were determined by two-way ANOVA and Bonferroni’s multiple comparison test. *P<0.05, **p<0.01, ***p<0.001. (**J**) Representative blots of p-CREB, CREB (~46 kDa), BDNF (~14 kDa), p-TrkB, TrkB (~140 kDa), and β-actin (~42 kDa) in the hippocampi of Swiss Albino mice (**J**). ^#^p-CREB, BDNF, and TrkB come from the same blot, thus sharing the corresponding β-actin band.

6-Hz seizures did not significantly affect the activation of CREB or TrkB in the hippocampus or BDNF protein expression. A single dose of ZnCl_2_ (8 mg Zn/kg) increased the p-CREB/CREB ratio (**Fig S7 G**) and BDNF protein level (**Fig S7 H**) in the hippocampus of sham mice. A single dose of TC-G 1008 (40 mg/kg) increased the p-TrkB/TrkB ratio in the hippocampus of sham mice (**Fig S7 F**). In addition, a single dose of VPA (50 mg/kg) increased the p-CREB/CREB ratio (**Fig S7 A**) and BDNF protein level (**Fig S7 B**) in the hippocampus of mice that received 6-Hz seizures, compared to VEH.

**Fig S8**

**
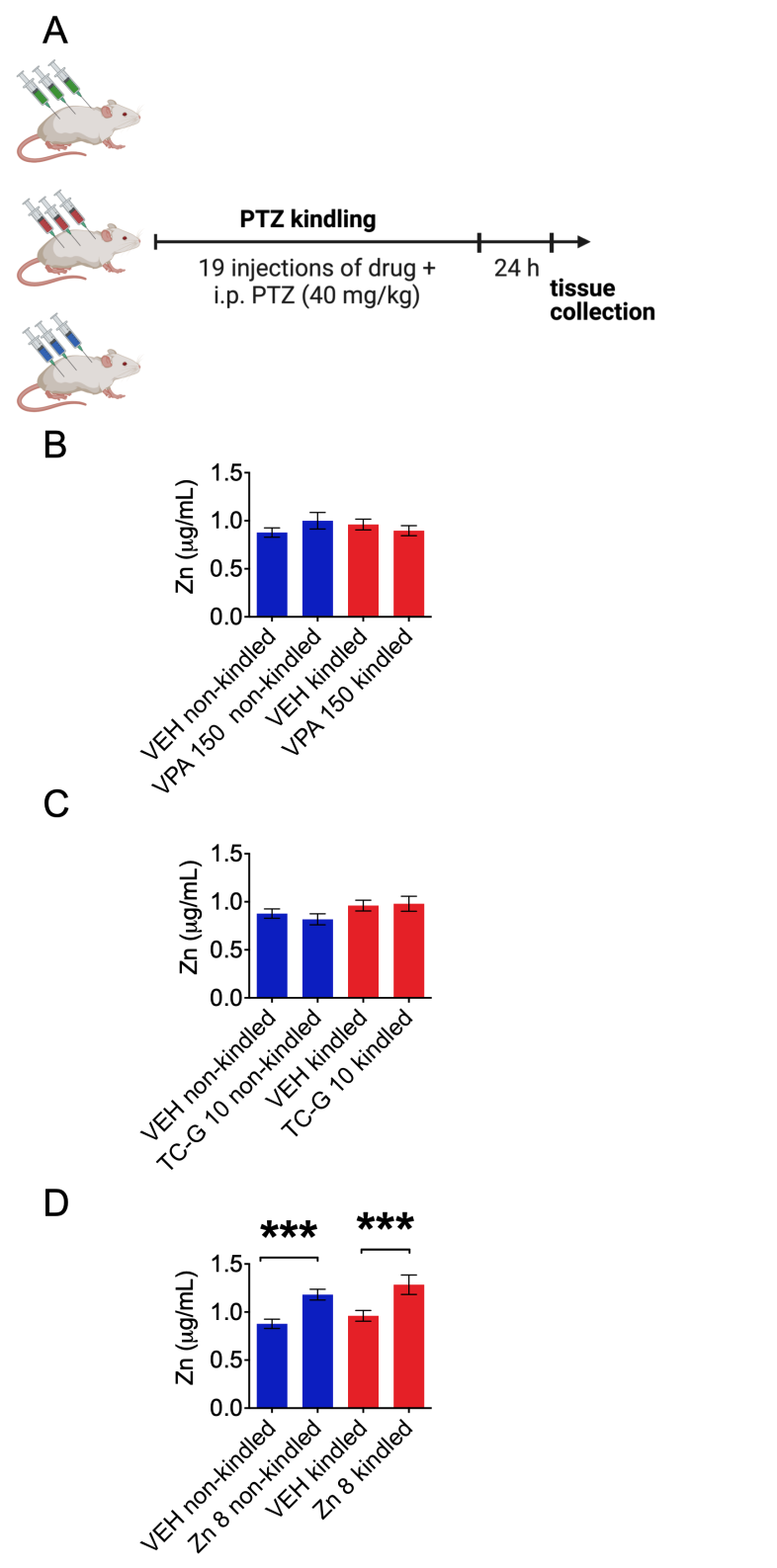
**

**Fig S8. *The effects of chronic treatment with VPA, TC-G 1008, or ZnCl_2_ and the PTZ-induced kindling model of epilepsy on total serum zinc concentration. (A)*** *Experimental paradigm****.*** Drugs: VPA (150 mg/kg), TC-G 1008 (10 mg/kg), ZnCl_2_ (8 mg Zn/kg), or VEH were injected i.p. once daily on every alternate day during weekdays. 30 min later, PTZ (40 mg/kg) was injected i.p. Non-kindled mice received the respective doses of drugs or VEH but received physiological saline instead of PTZ. Kindling consisted of 19 injections of PTZ (40 mg/kg). Tissues for biochemical analyses were obtained 24 h after the completion of the kindling paradigm. **(B-D)** Total serum zinc concentration was measured by ICP-OES. Data are expressed as means ± SEM. P values were determined by two-way ANOVA and Bonferroni’s multiple comparison test. ***P<0.01.

The chronic PTZ-kindling model of epilepsy did not significantly affect total serum zinc concentration (**Fig S8 B-D**). Serum zinc concentration increased after chronic treatment with ZnCl_2_ (8 mg Zn/kg) in both kindled and non-kindled mice (**Fig S8 D**). This effect was not observed in either kindled or non-kindled mice after treatment with TC-G 1008 (**Fig S8 C**) or VPA (**Fig S8 B**).

**Fig S9**

**
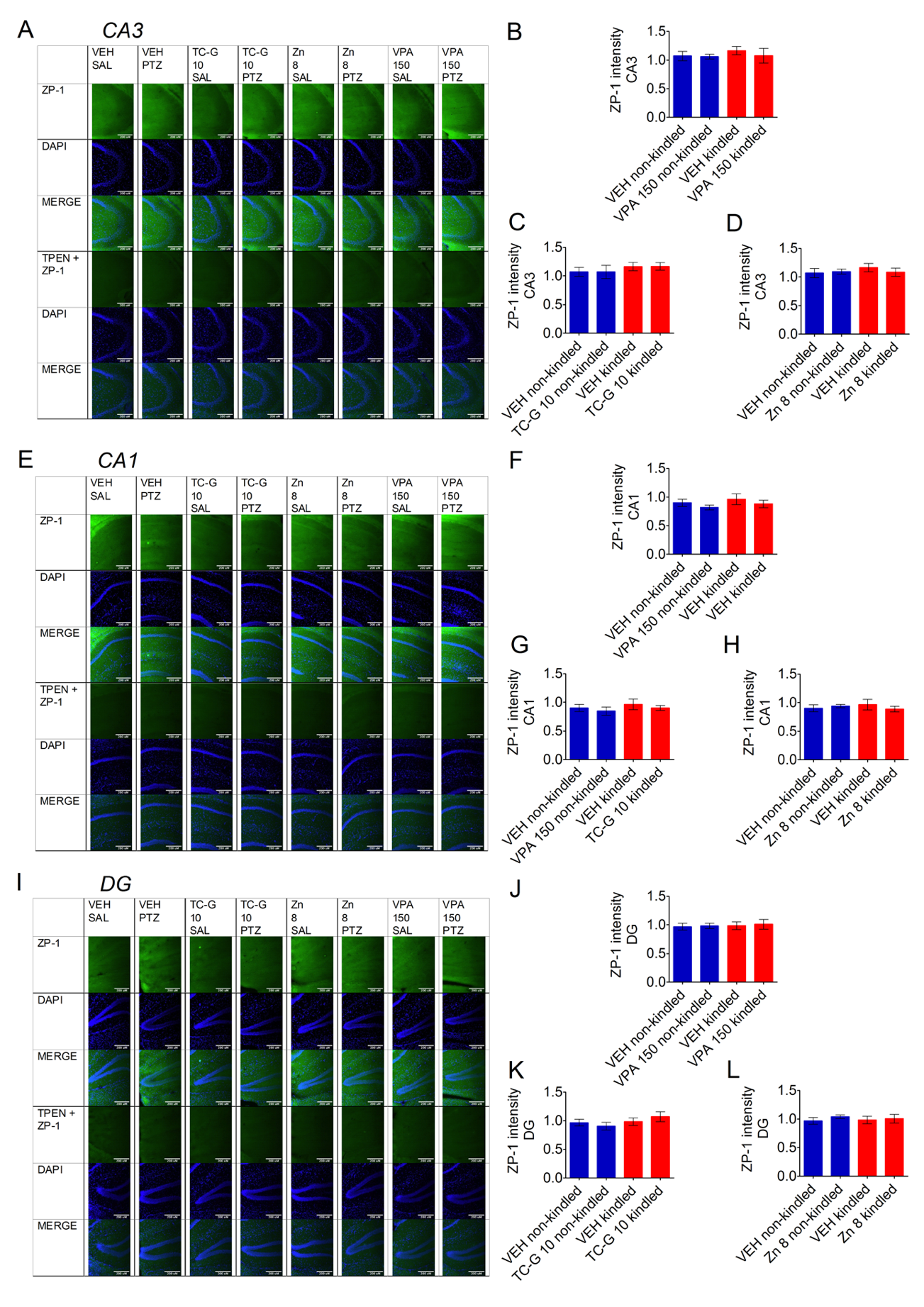
**

**Fig S9. *The effects of chronic treatment with VPA, TC-G 1008, or ZnCl_2_ and the PTZ kindling model of epilepsy on [Zn^2+^]_I_ in the CA3, CA1, and DG regions of the hippocampus.*** Hippocampal sections from mice were obtained 24 h after the completion of the kindling paradigm. The ratio of mean ZP-1 grey values between mouse sections belonging to treatment groups: VPA (**B, F, J**), TC-G 1008 (**C, G, K**), or ZnCl_2_ (**D, H, L**) is shown. Data were analyzed by two-way ANOVA. Magnifications of ZP-1 (green), DAPI (blue), and TPEN / ZP-1 in the CA3 (**A**), CA1 (**E**), and DG (**I**) regions are shown. Merged images include ZP-1 (green), TPEN / ZP-1, and DAPI (blue). ZP-1 fluorescence is primarily absent in TPEN-treated sections. Scale bar=200 μm.

***Hippocampal [Zn^2+^]_I_ was not significantly different after chronic treatment with TC-G 1008 in the PTZ-kindling model of epilepsy***

At 24 h after the last injection of PTZ in the chronic, PTZ-induced kindling model of epilepsy, none of the applied doses of administered compounds nor PTZ-kindling significantly affected hippocampal [Zn^2+^]_I_ (**Fig S9**).

**Fig S10.**

**
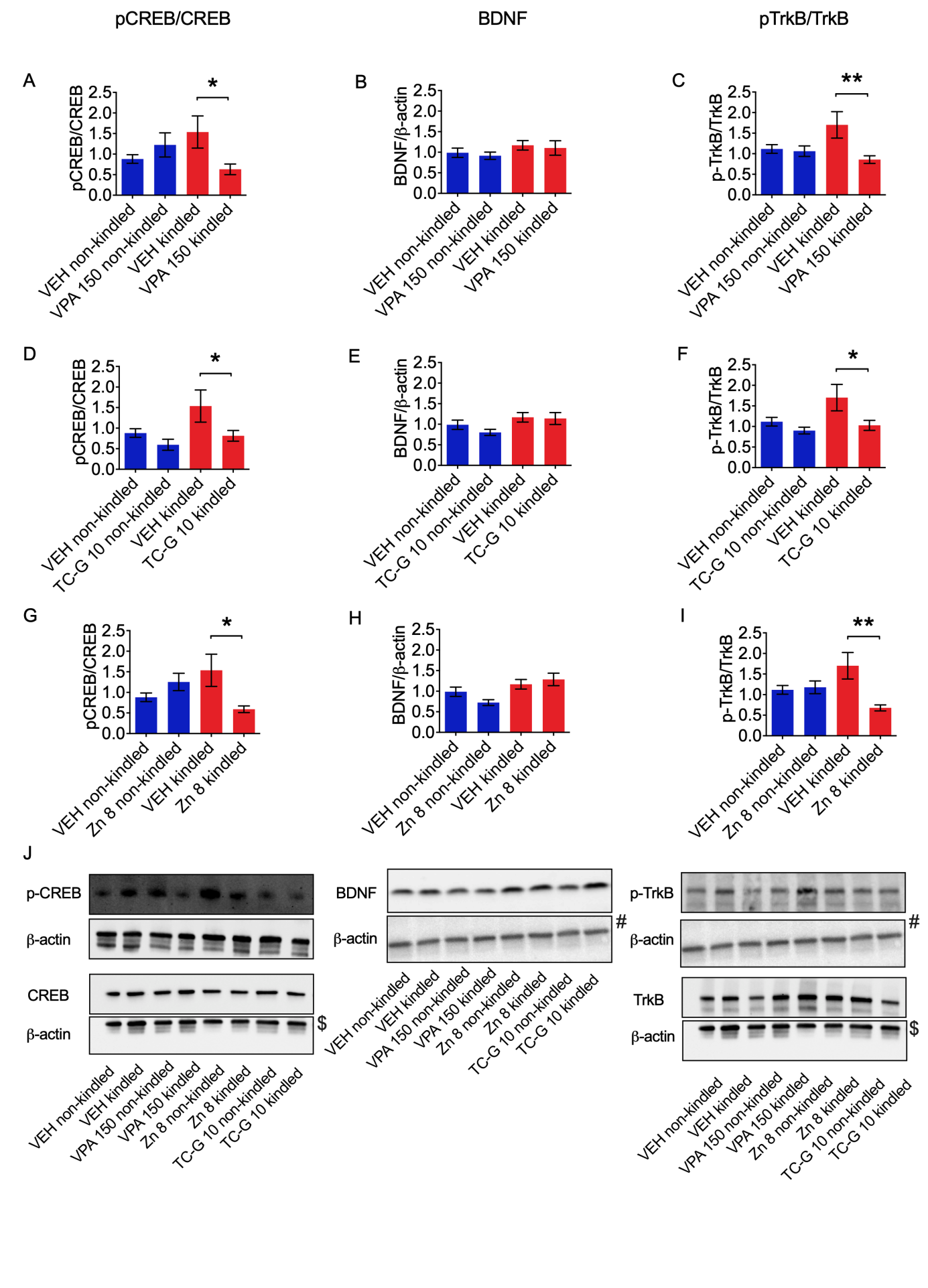
**

**Fig *S10. The effects of chronic treatment with VPA, TC-G 1008, or ZnCl_2_ and the PTZ kindling model of epilepsy on the expression of various proteins (phosphorylated CREB at Ser 133 (p-CREB), total CREB, BDNF, phosphorylated TrkB at Tyr 816 (p-TrkB) or total TrkB) in the hippocampus.*** Hippocampal samples were obtained 24 h after the completion of the kindling paradigm. The results (mean ± SEM) are presented as the p-CREB/CREB or BDNF/β-actin or p-TrkB/TrkB ratio. P values were determined by two-way ANOVA and Bonferroni’s multiple comparison test (**A-I**). *P<0.05, **p<0.01. Representative blots of p-CREB, CREB (~46 kDa), BDNF (~14 kDa), p-TrkB, TrkB (~140 kDa), and β-actin (~42 kDa) in the hippocampi of mice (**J**). ^#^BDNF and p-TrkB come from the same blot; ^$^CREB and TrkB come from the same blot, thus sharing the corresponding β-actin band.

The chronic PTZ-kindling model of epilepsy consisted of 19 injections of PTZ (40 mg/kg). After treatment, there was a trend towards increased activation of CREB and TrkB in the hippocampus of mice 24 h after the last injection of PTZ. Chronic treatment with VPA (150 mg/kg) decreased the p-CREB/CREB ratio (**Fig S10 A**) and p-TrkB/TrkB ratios (**Fig S10 C**) in the hippocampus of kindled mice. Similarly, chronic administration of TC-G 1008 (10 mg/kg) decreased the p-CREB/CREB (**Fig S10 D**) and p-TrkB/TrkB (**Fig S10 F**) ratios in the hippocampus of kindled mice. Also, administration of ZnCl_2_ (8 mg Zn/kg) decreased the p-CREB/CREB (**Fig S10 G**) and p-TrkB/TrkB (**Fig S10 I**) ratios in the hippocampus of kindled mice. Neither the administered compounds nor the PTZ-kindling model significantly affected BDNF protein level (**Fig S10 B, E, H**).

**Fig S11**

**
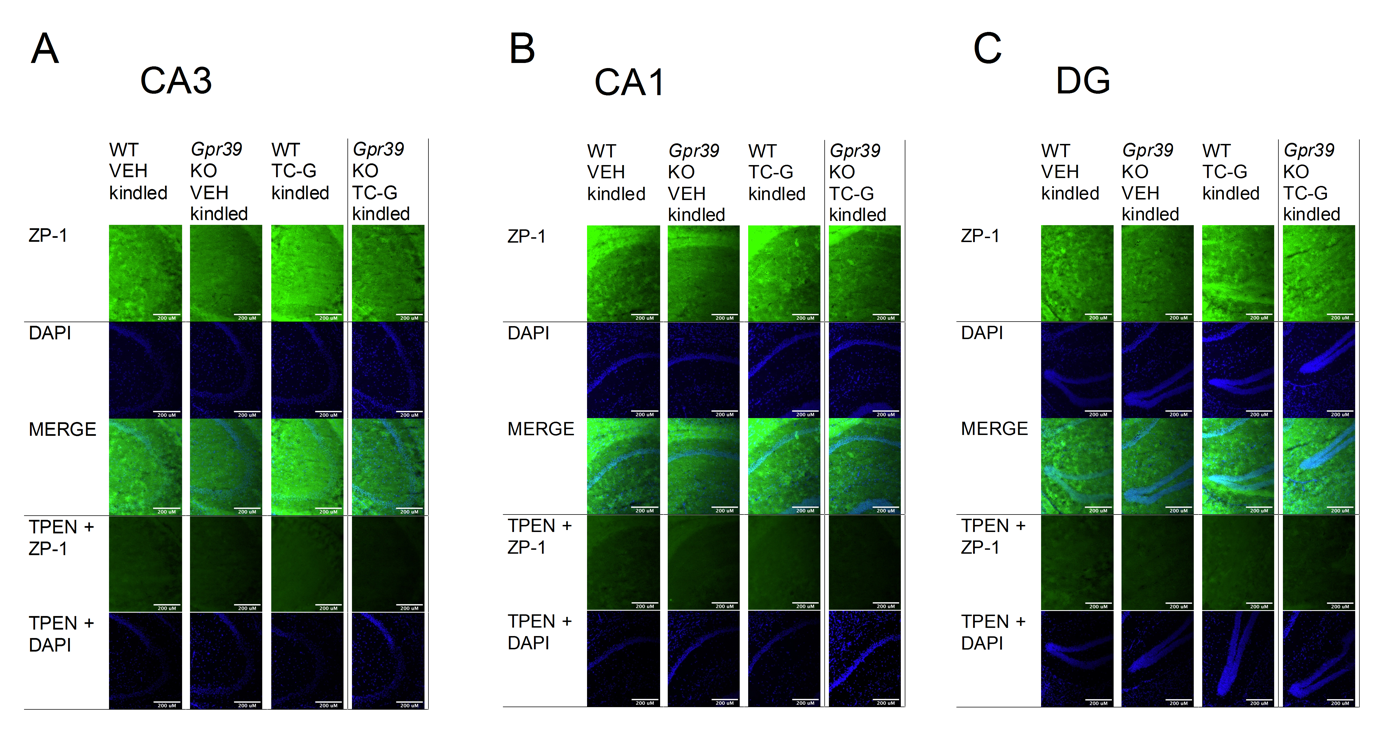
**

**Fig S11.** Magnification of ZP-1 (green), DAPI (blue), and TPEN / ZP-1 in the CA3 (**A**), CA1 (**B**), and DG (**C**) of GPR39 KO or WT (C57BL/6/Tar × CBA/Tar) mice subjected to the PTZ-kindling model of epilepsy and chronic treatment with TC-G 1008. Merged images include ZP-1 (green), TPEN / ZP-1, and DAPI (blue). ZP-1 fluorescence is primarily absent in the TPEN-treated sections, thus revealing the high specificity of the Zn2+-staining in brain sections. Scale bar=200 μm.

**Fig S12**

**
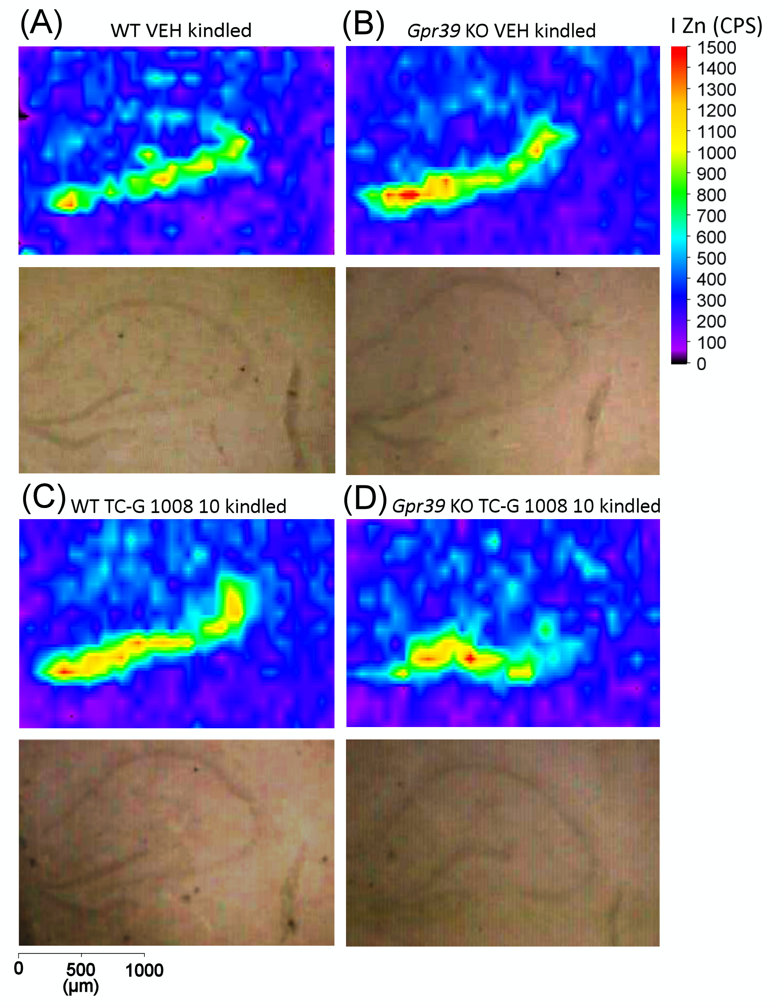
**

**Fig S12. Upper panel:** Examples of images of total zinc taken by laser ablation inductively coupled plasma mass spectrometry in coronal sections from GPR39 KO or WT (C57BL/6/Tar × CBA/Tar) mice subjected to the PTZ-kindling model of epilepsy and chronic treatment with TC-G 1008**. Lower panel:** images of the corresponding hippocampal samples taken prior to ablation. CPS - counts per second.

**Statistical details**

**Fig S1**

**(B)** t(20)=6.395, p<0.0001; n=12 VEH, n=10 VPA 150

**(C)** t(19)=6.235, p<0.0001; n=12 VEH, n=9 VPA 150

**(D)** t(19)=3.689, p=0.0016); n=12 VEH, n=9 VPA 150

(**B-D** Student’s t-test)

**(E)** F(3, 37)=4.022, p=0.0142; n=12 VEH, n=10 TC-G 1008 10, n=10 TC-G 1008 20, n=9 TC-G 1008 40

**(F)** F(3, 35)=2.008, p=0.1308; n=12 VEH, n=10 TC-G 1008 10, n=9 TC-G 1008 20, n=9 TC-G 1008 40, outlier excluded in TC-G 20 group, thus the analysis was based on n=12 VEH, n=10 TC-G 1008 10, n=8 TC-G 1008 20, n=9 TC-G 1008 40

**(G)** F(3, 34)=0.9246, p=0.4394; n=12 VEH, n=9 TC-G 1008 10, n=8 TC-G 1008 20, n=9 TC-G 1008 40

**(H)** F(3, 40)=0.5076, p=0.5076; n=12 VEH, n=11 Zn 8, n=11 Zn 16, n=10 Zn 32

**(I)** F(3, 37)=3.787, p=0.0182; n=12 VEH, n=10 Zn 8, n=9 Zn 16, n=11 Zn 32

**(J)** F(3, 38)=5.253, p=0.0039; n=12 VEH, n=10 Zn 8, n=11 Zn 16, n=9 Zn 32

(**E-J** one-way ANOVA)

**Fig S2**

**(B)** interaction [F(1,35)=0.01585, p=0.9005], VPA 150 [F(1,35)=11.487, p=0.0018], MES [F(1,35)=1.154, p=0.29]; n=10 in each group, outlier excluded in VEH sham group, thus the analysis was based on n=9 VEH sham, n=10 VPA 150 sham, n=10 VEH MES, n=10 VPA 150 MES

**(C)** interaction [F(1,29)=1.161, p=0.2902], TC-G 1008 2.5 [F(1,29)=1.743, p=0.1971], MES [F(1,29)=15.04, p=0.0006]; n=10 VEH sham, n=8 VEH MES, n=10 TC-G 1008 2.5 sham, n=7 TC-G 1008 2.5 MES, outliers excluded in VEH sham and TC-G 1008 2.5 sham groups, thus the analysis was based on n=9 VEH sham, n=8 VEH MES, n=9 TC-G 1008 2.5 sham, n=7 TC-G 1008 2.5 MES

**(D)** interaction [F(1,35)=1.88, p=0.1791], TC-G 1008 20 [F(1,35)=0.0042637, p=0.9483], MES [F(1,35)=5.372, p=0.0264]; n=10 in each group, outlier excluded in VEH sham group, thus the analysis was based on n=9 VEH sham, n=10 VEH MES, n=10 TC-G 1008 2.5 sham, n=10 TC-G 1008 2.5 MES

**(E)** interaction [F(1,35)=6.822, p=0.0132], Zn 4 [F(1,35)=80.05, p<0.0001], MES [F(1,35)=7.596, p=0.29]; n=10 in each group, outlier excluded in VEH sham group, thus the analysis was based on n=9 VEH sham, n=10 VEH MES, n=10 Zn 4 sham, n=10 Zn 4 MES

**(F)** interaction [F(1,26)=2.696, p=0.1104], Zn 16 [F(1,35)=122.5, p<0.0001], MES [F(1,35)=3.097, p=0.088]; n=10 VEH sham, n=8 VEH MES, n=10 Zn 16 sham, n=9 Zn 16 MES, outlier excluded in VEH sham group, thus the analysis was based on n=9 VEH sham, n=8 VEH MES, n=10 Zn 4 sham, n=9 Zn 16 MES

(**B-F** one-way ANOVA)

**Fig S3**

**(B)** interaction [F(1,16)=0.03263, p=0.8589], VPA 150 [F(1,16)= 4.264, p=0.0555], MES [F(1,16)= 1.791, p=0.1995]; n=5 in each group

**(C)** interaction [F(1,16)=2.056, p=0.1708], TC-G 1008 2.5 [F(1,16)=9.034, p=0.0084], MES [F(1,16)= 0.7403, p=0.4023]; n=5 in each group

**(D)** interaction [F(1,16)=1.357, p=0.2612], TC-G 1008 20 [F(1,16)=1.668, p=0.2149], MES [F(1,16)=0.03546, p=0.853]; n=5 in each group

**(E)** interaction [F(1,15)=0.7942, p=0.3869], Zn 4 [F(1,15)=8.5, p=0.0107], MES [F(1,15)= 0.04685, p=0.8316]; n=5 in each group, sample loss in Zn 4 MES group, thus the analysis was based on n=5 VEH sham, n=5 Zn 4 sham, n=5 VEH MES, n=4 Zn 4 MES

**(F)** interaction [F(1,16)=0.04982, p=0.8262], Zn 16 [F(1,16)=0.5478, p=0.4699], MES [F(1,16)= 3.203, p=0.0925]; n=5 in each group

**(H)** interaction [F(1,16)=0.01224, p=0.9133], VPA 150 [F(1,16)=3.693, p=0.0726], MES [F(1,16)=0.4103, p=0.5309]; n=5 in each group

**(I)** interaction [F(1,16)=3.377, p=0.0848], TC-G 1008 2.5 [F(1,16)=9.198, p=0.0079], MES [F(1,16)=2.163, p=0.1607]; n=5 in each group

**(J)** interaction [F(1,16)=0.8636, p=0.3665], TC-G 1008 20 [F(1,16)=0.1068, p=0.748], MES [F(1,16)=0.005671, p=0.9409]; n=5 in each group

**(K)** interaction [F(1,15)=0.1167, p=0.7373], Zn 4 [F(1,15)=8.268, p=0.0116], MES [F(1,15)=0.1663, p=0.6892]; n=5 in each group, sample loss in Zn 4 MES group, thus the analysis was based on n=5 VEH sham, n=5 Zn 4 sham, n=5 VEH MES, n=4 Zn 4 MES

**(L)** interaction [F(1,16)=0.1492, p=0.7044], Zn 16 [F(1,16)=0.3807, p=0.5459], MES [F(1,16)=4.087, p=0.0603]; n=5 in each group

**(N)** interaction [F(1,15)=1.386, p=0.2574], VPA 150 [F(1,15)=5.565, p= 0.0323], MES [F(1,15)=0.01234, p=0.913]; n=5 in each group, sample loss in VPA 150 sham group, thus the analysis was based on n=5 VEH sham, n=4 VPA 150 sham, n=5 VEH MES, n=5 VPA 150 MES

(**O**) interaction [F(1,16)=2.202, p=0.1573], TC-G 1008 2.5 [F(1,16)=3.424, p=0.0828], MES [F(1,16)=0.941, p=0.3465]; n=5 in each group

**(P)** interaction [F(1,16)=0.00189, p=0.9658], TC-G 1008 20 [F(1,16)=2.566, p=0.1288], MES [F(1,16)=2.295, p=0.1493]; n=5 in each group

**(Q)** interaction [F(1,15)=0.07936, p=0.782], Zn 4 [F(1,15)= 4.254, p=0.0569], MES [F(1,15)=2.217, p=0.1572]; n=5 in each group, sample loss in Zn 4 sham group, thus the analysis was based on n=5 VEH sham, n=4 Zn 4 sham, n=5 VEH MES, n=5 Zn 4 MES

**(R)** interaction [F(1,16)=0.04564, p=0.8335], Zn 16 [F(1,16)=0.5955, p=0.4515], MES [F(1,16)= 2.996, p=0.1027; n=5 in each group

**(B-F, H-L, N-R** Two-way ANOVA)

**Fig S4**

**(A)** interaction [F(1,20)=1.05, p=0.3178], VPA 150 [F(1,20)=5.56, p= 0.0287], MES [F(1,20)=4.414, p=0.0485]; n=7 in each group, samples damaged due to technical failure or outliers were excluded, thus the analysis was based on n=6 VEH sham, n=7 VPA 150 sham, n=4 VEH MES, n=7 VPA 150 MES

**(B)** interaction [F(1,24)=0.007895, p=0.9299], VPA 150 [F(1,24)=3.365, p= 0.079], MES [F(1,24)=0.2656, p=0.611]; n=7 in each group

**(C)** interaction [F(1,18)=6.457, p=0.0205], VPA 150 [F(1,18)=7.52, p=0.0134], MES [F(1,18)=9.908, p=0.0056]; n=7 in each group, samples damaged due to technical failure or outliers were excluded, thus the analysis was based on n=6 VEH sham, n=5 VPA 150 sham, n=6 VEH MES, n=6 VPA 150 MES

**(D)** interaction [F(1,28)= 2.823, p=0.1041], TC-G 1008 2.5 [F(1,28)=5.575, p=0.0254], MES [F(1,28)= 0.1217, p=0.7298]; n=8 in each group

**(E)** interaction [F(1,19)= 1.054, p=0.3174], TC-G 1008 20 [F(1,19)=5.572, p=0.0291], MES [F(1,19)= 4.773, p=0.0416]; n=7 in each group, samples damaged due to technical failure or outliers were excluded, thus the analysis was based on n=6 VEH sham, n=6 TC-G 1008 20 sham, n=4 VEH MES, n=7 TC-G 1008 20 MES

**(F)** interaction [F(1,28)=0.3767, p=0.5443], TC-G 1008 2.5 [F(1,28)=5.93, p=0.0215], MES [F(1,28)=2.102, p=0.1582]; n=8 in each group

**(G)** interaction [F(1,24)=0.3232, p=0.575], TC-G 1008 20 [F(1,24)=2.355, p=0.1379], MES [F(1,24)=1.165, p=0.2911]; n=7 in each group

(**H**) interaction [F(1,25)=0.332, p=0.5696], TC-G 1008 2.5 [F(1,25)= 8.33, p=0.0079], MES [F(1,25)= 1.085, p=0.3076]; n=8 in each group, samples damaged due to technical failure were excluded, thus the analysis was based on n=8 VEH sham, n=7 TC-G 1008 2.5 sham, n=8 VEH MES, n=7 TC-G 1008 2.5 MES

**(I)** interaction [F(1,20)=1.05, p= 0.3177], TC-G 1008 20 [F(1,20)= 2.589, p=0.1233], MES [F(1,20)= 4.716, p= 0.3177]; n=7 in each group, samples damaged due to technical failure were excluded, thus the analysis was based on n=6 VEH sham, n=6 TC-G 1008 20 sham, n=6 VEH MES, n=7 TC-G 1008 20 MES

**(J)** interaction [F(1,20)= 0.735, p= 0.4014], Zn 4 [F(1,20)= 4.755, p= 0.0413], MES [F(1,20)= 0.2141, p= 0.6486]; n=7 in each group, samples damaged due to technical failure or outliers were excluded, thus the analysis was based on n=6 VEH sham, n=7 Zn 4 sham, n=4 VEH MES, n=7 Zn 4 MES

**(K)** interaction [F(1,28)=0.9397, p=0.3406], Zn 16 [F(1,28)=1.727, p=0.1994], MES [F(1,28)= 0.9847, p= 0.3295]; n=8 in each group

**(L)** interaction [F(1,24)=0.2363, p=0.6313], Zn 4 [F(1,24)=0.8156, p=0.3754], MES [F(1,24)=0.4332, p=0.5167]; n=7 in each group

**(M)** interaction [F(1,28)=6.043, p=0.0204], Zn 16 [F(1,28)=0.2976, p=0.5897], MES [F(1,28)=0.06256, p=0.8043] n=8 in each group

**(N)** interaction [F(1,21)=3.435, p=0.0779], Zn 4 [F(1,21)=3.071, p=0.0943], MES [F(1,21)=8.298, p=0.0089]; n=7 in each group, samples damaged due to technical failure or outlier were excluded, thus the analysis was based on n=6 VEH sham, n=7 Zn 4 sham, n=6 VEH MES, n=7 Zn 4 MES

**(O)** interaction [F(1,26)=4.02, p=0.0555], Zn 16 [F(1,26)=0.1984, p= 0.6597], MES [F(1,26)= 0.000525, p=0.9819]; n=8 in each group, samples damaged due to technical failure were excluded, thus the analysis was based on n=8 VEH sham, n=7 Zn 16 sham, n=8 VEH MES, n=7 Zn 16 MES

**(A-O** Two-way ANOVA)

**Fig S5**

**(B)** interaction [F(1,28)=1.627, p=0.2125], VPA 50 [F(1,28)=0.1678, p=0.6852], 6-Hz [F(1,28)=1.459, p=0.2371]; n=8 in each group

**(C)** interaction [F(1,28)=4.684, p=0.0391], TC-G 1008 40 [F(1,28)=3.135, p=0.0875], 6-Hz [F(1,28)= 4.393, p=0.0452]; n=8 in each group

**(D)** interaction [F(1,28)=0.2557, p=0.617], Zn 8 [F(1,28)=189.2, p<0.0001], 6-Hz [F(1,28)= 0.2601, p=0.6141], n=8 in each group

**(B-D** Two-way ANOVA)

**Fig S6**

**(A)** interaction [F(1,24)=3.813, p=0.0626], VPA 50 [F(1,24)= 13.62, p=0.0011], 6-Hz [F(1,24)=15.74, p=0.0006]; n=7 in each group

**(B)** interaction [F(1,23)=0.5007, p=0.4863], VPA 50 [F(1,23)=8.737, p=0.0071], 6-Hz seizure [F(1,23)=2.859, p=0.1044]; n=7 in each group, outliers excluded, thus the analysis based on n=6 VEH sham, n=7 VPA 50 sham, n=7 VEH 6-Hz, n=7 VPA 50 6-Hz

**(C)** interaction [F(1,22)=0.07561, p=0.7859], VPA 50 [F(1,22)=3,342, p=0.0811], 6-Hz seizure [F(1,22)=0.4019, p=0.5326]; n=7 in each group, samples damaged due to technical failure were excluded, thus the analysis was based on n=5 VEH sham, n=7 TC-G 1008 40 sham, n=7 VEH 6-Hz, n=7 TC-G 1008 40 6-Hz

**(D)** interaction [F(1,24)= 1.058, p=0.3139], TC-G 1008 40 [F(1,24)= 1.288, p=0.2676], 6-Hz [F(1,24)= 0.404, p=0.531]; n=7 in each group

**(E)** interaction [F(1,23)=1.526, p=0.2292], TC-G 1008 40 [F(1,23)=2.708, p=0.1134], 6-Hz seizure [F(1,23)=0.5955, p=0.4482]; n=7 in each group, outliers excluded, thus the analysis was based on n=6 VEH sham, n=7 TC-G 1008 40 sham, n=7 VEH 6-Hz, n=7 TC-G 1008 40 6-Hz

**(F)** interaction [F(1,22)=1.803, p=0.1931], TC-G 1008 40 [F(1,22)=5.526, p=0.0281], 6-Hz seizure [F(1,22)=1.377, p=0.2532]; n=7 in each group, samples damaged due to technical failure were excluded, thus the analysis was based on n=5 VEH sham, n=7 TC-G 1008 40 sham, n=7 VEH 6-Hz, n=6 TC-G 1008 40 6-Hz

**(G)** interaction [F(1,23)=2.925, p=0.1007], Zn 8 [F(1,23)=12.29, p=0.0019], 6-Hz [F(1,23)= 0.09777, p=0.7573]; n=7 in each group

(**H**) interaction [F(1,23)=11,76, p=0.0023], Zn 8 [F(1,23)=6,87, p=0.0153], 6-Hz seizure [F(1,23)=3,113, p=0.0909]; n=7 in each group, outlier excluded, thus the analysis was based on n=6 VEH sham, n=7 Zn 8 sham, n=7 VEH 6-Hz, n=7 Zn 8 6-Hz

**(I)** interaction [F(1,22)=0.01181, p=0.9144], Zn 8 [F(1,22)= 5.422, p=0.0295], 6-Hz seizure [F(1,22)=0.08987, p=0.7672]; n=7 in each group, samples damaged due to technical failure were excluded, thus the analysis was based on n=5 VEH sham, n=7 TC-G 1008 40 sham, n=7 VEH 6-Hz, n=7 TC-G 1008 40 6-Hz

**(A-I** Two-way ANOVA)

**Fig S7**

**(A)** interaction [F(1,24)=3.813, p=0.0626], VPA 50 [F(1,24)= 13.62, p=0.0011], 6-Hz [F(1,24)=15.74, p=0.0006]; n=7 in each group

**(B)** interaction [F(1,23)=0.5007, p=0.4863], VPA 50 [F(1,23)=8.737, p=0.0071], 6-Hz seizure [F(1,23)=2.859, p=0.1044]; n=7 in each group, outliers excluded, thus the analysis was based on n=6 VEH sham, n=7 VPA 50 sham, n=7 VEH 6-Hz, n=7 VPA 50 6-Hz

**(C)** interaction [F(1,22)=0.07561, p=0.7859], VPA 50 [F(1,22)=3,342, p=0.0811], 6-Hz seizure [F(1,22)=0.4019, p=0.5326]; n=7 in each group, samples damaged due to technical failure were excluded, thus the analysis was based on n=5 VEH sham, n=7 TC-G 1008 40 sham, n=7 VEH 6-Hz, n=7 TC-G 1008 40 6-Hz

**(D)** interaction [F(1,24)= 1.058, p=0.3139], TC-G 1008 40 [F(1,24)= 1.288, p=0.2676], 6-Hz [F(1,24)= 0.404, p=0.531]; n=7 in each group

**(E)** interaction [F(1,23)=1.526, p=0.2292], TC-G 1008 40 [F(1,23)=2.708, p=0.1134], 6-Hz seizure [F(1,23)=0.5955, p=0.4482]; n=7 in each group, outliers excluded, thus the analysis was based on n=6 VEH sham, n=7 TC-G 1008 40 sham, n=7 VEH 6-Hz, n=7 TC-G 1008 40 6-Hz

**(F)** interaction [F(1,22)=1.803, p=0.1931], TC-G 1008 40 [F(1,22)=5.526, p=0.0281], 6-Hz seizure [F(1,22)=1.377, p=0.2532]; n=7 in each group, samples damaged due to technical failure were excluded, thus the analysis was based on n=5 VEH sham, n=7 TC-G 1008 40 sham, n=7 VEH 6-Hz, n=6 TC-G 1008 40 6-Hz

**(G)** interaction [F(1,23)=2.925, p=0.1007], Zn 8 [F(1,23)=12.29, p=0.0019], 6-Hz [F(1,23)= 0.09777, p=0.7573]; n=7 in each group

(**H**) interaction [F(1,23)=11,76, p=0.0023], Zn 8 [F(1,23)=6,87, p=0.0153], 6-Hz seizure [F(1,23)=3,113, p=0.0909]; n=7 in each group, outlier excluded, thus the analysis was based on n=6 VEH sham, n=7 Zn 8 sham, n=7 VEH 6-Hz, n=7 Zn 8 6-Hz

**(I)** interaction [F(1,22)=0.01181, p=0.9144], Zn 8 [F(1,22)= 5.422, p=0.0295], 6-Hz seizure [F(1,22)=0.08987, p=0.7672]; n=7 in each group, samples damaged due to technical failure were excluded, thus the analysis based on n=5 VEH sham, n=7 TC-G 1008 40 sham, n=7 VEH 6-Hz, n=7 TC-G 1008 40 6-Hz

**(A-I** Two-way ANOVA)

**Fig S8**

**(B)** interaction [F(1,26)=3.134, p=0.0865], VPA 150 [F(1,26)=0.05726, p=0.8124], PTZ kindling [F(1,26)=3.134, p=0.0865]; n=10 in each group

**(C)** interaction [F(1,27)=6.882, p=0.0144], TC-G 1008 10 [F(1,27)=8.387, p=0.0076], PTZ kindling [F(1,27)=10.65, p=0.0031]; n=10 in each group

**(D)** interaction [F(1,27)=0.04264, p=0.8377], Zn 8 [F(1,27)=35.69, p<0.0001], PTZ kindling [F(1,27)=6.034, p=0.0195]; n=10 in each group

**(B-D** Two-way ANOVA)

**Fig S9**

**(B)** interaction [F (1, 16)=0.1947, p=0.6649], VPA 150 [F (1, 16)=0.3107, p=0.850], PTZ kindling [F (1, 16)=0.4079, p=0.5321]; n=5 in each group

**(C)** interaction [F (1, 16)=0.0006, p=0.9814], TC-G 1008 10 [F (1, 16)=0.0006, p=0.9814], PTZ kindling [F (1, 16)=1.265, p=0.2772]; n=5 in each group

**(D)** interaction [F (1, 16)=0.5715, p=0.4607], Zn 8 [F (1, 16)=0.1750, p=0.6813], PTZ kindling [F (1, 16)=0.3711, p=0.5510]; n=5 in each group

**(F)** interaction [F (1, 16)=0.000, p>0.9999], VPA 150 [F (1, 16)=1.378, p=0.2576], PTZ kindling [F (1, 16)=0.8626, p=0.3668]; n=5 in each group

**(G)** interaction [F (1, 16)=0.0051, p=0.9438], TC-G 1008 10 [F (1, 16)=0.6984, p=0.4156], PTZ kindling [F (1, 16)=0.6984, P=0.4156]; n=5 in each group

**(H)** interaction [F (1, 16)=0.0797, p=0.7814], Zn 8 [F (1, 16)=0.0078, p=0.9305], PTZ kindling [F (1, 16)=0.0006, p=0.9810]; n=5 in each group

**(J)** interaction [F (1, 16)=0.3412, p=0.5673], VPA 150 [F (1, 16)= .2439, p=0.6281], PTZ kindling [F (1, 16)=0.03236, p=0.8595]; n=5 in each group

**(K)** interaction [F (1, 16)=2.037, p=0.1727], TC-G 1008 10 [F (1, 16)=0.1308, p=0.7224], PTZ kindling [F (1, 16)=0.4940, p=0.4923]; n=5 in each group

**(L)** interaction [F (1, 16)=0.0289, p=0.8672], Zn 8 [F (1, 16)=0.8729, p=0.3640], PTZ kindling [F (1, 16)=0.4137, p=0.5292]; n=5 in each group

**(B-D, F-H, J-L** Two-way ANOVA).

**Fig S10**

**(A)** interaction [F(1,21)=6.552, p=0.0183], VPA 150 [F(1,21)=1.336, p= 0.2608], PTZ kindling [F(1,21)=0.01632, p=0.8996]; n=7 in each group, sample loss due to technical failure, thus the analysis was based on n=7 VEH VEH, n=7 VPA 150 VEH, n=5 VEH PTZ, n=6 VPA 150 PTZ

**(B)** interaction [F(1,23)=0.0631, p=0.8039], VPA 150 [F(1,23)=0.06271, p=0.8045], PTZ kindling [F(1,23)=2.994, p=0.097]; n=7 in each group

**(C)** interaction [F(1,24)=4.466, p=0.0452], VPA 150 [F(1,24)=5.799, p=0.0241], PTZ kindling [F(1,24)=1.042, p=0.3175]; n=7 in each group

**(D)** interaction [F(1,22)=1.346, p=0.2584], TC-G 1008 10 [F(1,22)=7.122, p=0.014], PTZ kindling [F(1,22)=5.333, p=0.0307]; n=7 in each group, sample loss due to technical failure, thus the analysis was based on n=7 VEH VEH, n=7 TC-G 1008 10 VEH, n=5 VEH PTZ, n=7 TC-G 1008 10 PTZ

**(E)** interaction [F(1,23)=0.1372, p=0.7145], TC-G 1008 10 [F(1,23)=0.3916, p=0.5376], PTZ kindling [F(1,23)=6.744, p=0.0161]; n=7 in each group

**(F)** interaction [F(1,24)=1.54, p=0.2266], TC-G 1008 10 [F(1,24)=5.815, p=0.0239], PTZ kindling [F(1,24)=3.722, p=0.0656]; n=7 in each group

**(G)** interaction [F(1,21)=10.01, p=0.0047], Zn 8 [F(1,21)=1.896, p=0.1831], PTZ kindling [F(1,21)=0.00035, p=0.9852]; n=7 in each group, sample loss due to technical failure, thus the analysis was based on n=7 VEH VEH, n=7 Zn 8 VEH, n=5 VEH PTZ, n=7 Zn 8 PTZ)

**(H)** interaction [F(1,22)=1.934, p=0.1783], Zn 8 [F(1,22)=0.1052, p=0.7487], PTZ kindling [F(1,22)=13.36, p=0.0014]; n=7 in each group, sample loss due to technical failure, thus the analysis was based on n=7 VEH VEH, n=7 Zn 8 VEH, n=7 VEH PTZ, n=6 Zn 8 PTZ

**(I)** interaction [F(1,24)=8.178, p=0.0086], Zn 8 [F(1,24)=6.442, p=0.0181], PTZ kindling [F(1,24)= 0.05032, p=0.8244]; n=7 in each group

**(A-I** Two-way ANOVA)
